# Enzymatic synthesis of *S*-adenosyl-l-homocysteine and its nucleoside analogs from racemic homocysteine thiolactone†

**DOI:** 10.1039/d4sc03801k

**Published:** 2024-09-06

**Authors:** Xiaojin Wen, Viviane Leopold, Florian P. Seebeck

**Affiliations:** a Department of Chemistry, University of Basel Mattenstrasse 22 Basel 4002 Switzerland florian.seebeck@unibas.ch; b Molecular Systems Engineering, National Competence Center in Research (NCCR) 4058 Basel Switzerland

## Abstract

*S*-Adenosyl methionine (SAM)-dependent methyltransferases hold significant potential as tools for the biocatalytic synthesis of complex molecules due to their ability to methylate or alkylate substrates with high regio-, chemo-, and stereoselectivity. Recent advancements in enzyme-catalyzed *S*-methylation and *S*-alkylation of *S*-adenosyl homocysteine (SAH) using synthetic alkylation agents have expanded the scope of methyltransferases in preparative biocatalysis. This development has transformed SAH from an unwanted byproduct into a crucial – and currently expensive – reagent. In this report, we present a simple and scalable one-pot synthesis of SAH, starting from racemic homocysteine thiolactone and adenosine. This process is catalyzed by recombinant α-amino-ε-caprolactam racemase, bleomycin hydrolase, and SAH hydrolase. The reaction proceeds to completion with near-stoichiometric mixtures of reactants, driven by the irreversible and stereoselective hydrolysis of thiolactone, followed by the thermodynamically favorable condensation of homocysteine with adenosine. We demonstrate that this method can be utilized to supplement preparative methylation reactions with SAH as a cofactor, as well as to synthesize and screen *S*-nucleosyl homocysteine derivatives in the search for stabilized SAM analogs.

## Abstract 2

One-pot enzyme-catalyzed synthesis of *S*-adenosyl homocysteine and derivatives thereof from racemic homocysteine thiolactone and adenosine or other nucleosides provides simple and scalable access to cofactors for methyltransferase biocatalysis.

## Graphical abstract

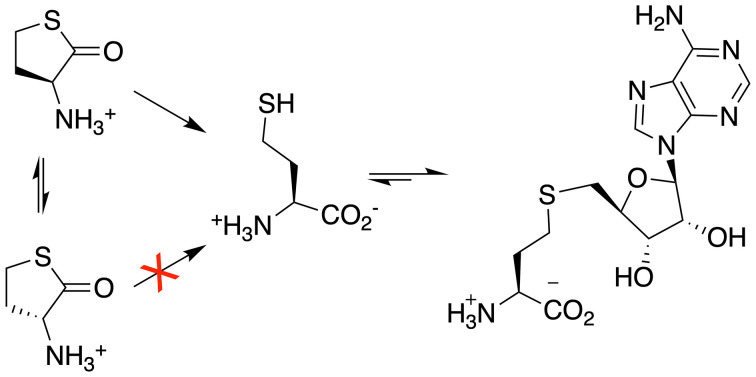

## Introduction

**Fig. 1:**
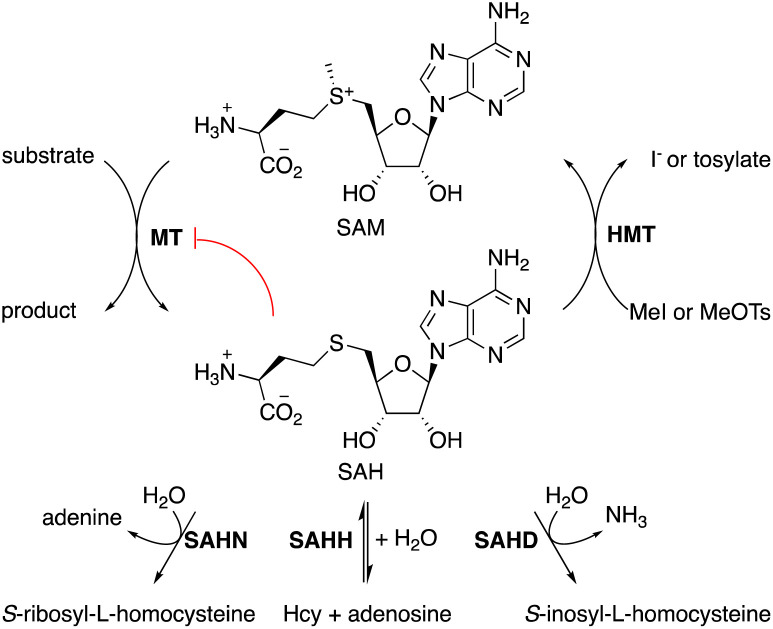
SAM-dependent methyltransferases are inhibited by accumulating SAH (red). The cellular metabolism can remove SAH by hydrolysis catalyzed by SAHN, SAHH, or SAHD, depending on the organism. To enable preparative methylation *in vitro* SAH may be remethylated by HMT using MeI or MeOTs as sacrificial methyl donors.

*S*-Adenosylmethionine (SAM)-dependent methyltransferases (MTs) catalyze the methylation of natural products,^1^ proteins, nucleic acids,^2,3^ and – less commonly – polysaccharides.^4^ Most MTs bind SAM in an active site that destabilizes the cationic charge on the sulfonium function to activate the methyl group for transfer. Transferring the methyl group to the substrate neutralizes the charge, thereby eliminating the associated destabilization. Consequently, the byproduct *S*-adenosyl homocysteine (SAH, Fig. 1) usually binds significantly stronger to MTs than SAM, with the result, that MT activity is generally prone to product inhibition. Indeed, the cellular SAM/SAH ratio is a critical parameter that affects methyltransferase-dependent signal transduction, transcriptional regulation, gene silencing, epigenetic control, and antibiotic resistance.^5–9^ To limit the accumulation of SAH, cells produce enzymes such as SAH hydrolase (SAHH, EC 3.13.2.1),^10^ SAH nucleosidase (SAHN, EC 3.2.2.9),^11^ or SAH deaminase (SAHD, EC 3.5.4.28).^12,13^ These enzymes catalyze initial hydrolytic steps in pathways that return the segregated components of SAH – homocysteine, ribose, and adenine or hypoxanthine – into the primary metabolism.

For applying MTs to *in vitro* biocatalysis, an alternative strategy for recycling SAH has proven more practical. Halide methyltransferases (HMTs) can methylate SAH to regenerate SAM using synthetic methyl donors such as methyl iodide (MeI) or methyl toluene sulfonate (MeOTs).^14,15^ This reaction, combined with a secondary MT that transfers methyl groups from SAM to a specific acceptor substrate amounts to a simple process for the preparative methylation of complex molecules (Fig. 1). In this process, SAH serves as a catalytic methyl transfer reagent.^14^ Subsequent work has demonstrated that HMTs and other MTs may also transfer larger and functionalized alkyl fragments to and from SAH, raising the possibility that MT biocatalysis may develop into a general strategy to accelerate selective alkylation reactions.^16–24^

Because of this development and the prospect of industrial applications of MT biocatalysis,^25–29^ a scalable supply of SAH may become an important asset. SAH has been prepared either by chemical synthesis,^30^ or by SAHH-catalyzed condensation of adenosine with l-homocysteine (l-Hcy).^31–34^ Both approaches suffer from the problem that l-Hcy is comparatively expensive and that reducing equivalents are required to suppress disulfide formation. To address this issue, we have developed a cost-effective, one-pot procedure to synthesize SAH from racemic Hcy thiolactone (at less than 1 CHF g^−1^). The reaction is catalyzed by three enzymes: a thiolactone racemizing enzyme (α-amino-ε-caprolactam racemase), a stereoselective l-Hcy-thiolactone hydrolase (bleomycin hydrolase), and SAHH. SAH formation is driven to completion by the irreversible hydrolysis of l-Hcy-thiolactone, followed by the thermodynamically favorable condensation of *in situ* generated l-Hcy with adenosine, using only stoichiometric amounts of both substrates. We demonstrate that this scalable SAH synthesis integrates seamlessly as a cofactor supply for preparative methyltransferase biocatalysis. Additionally, we utilize this method to synthesize nine *S*-nucleosyl homocysteines and investigate their potential as stabilized cofactors for methyltransferase biocatalysis.

## Results and discussion

### Strategy 1

**Fig. 2:**
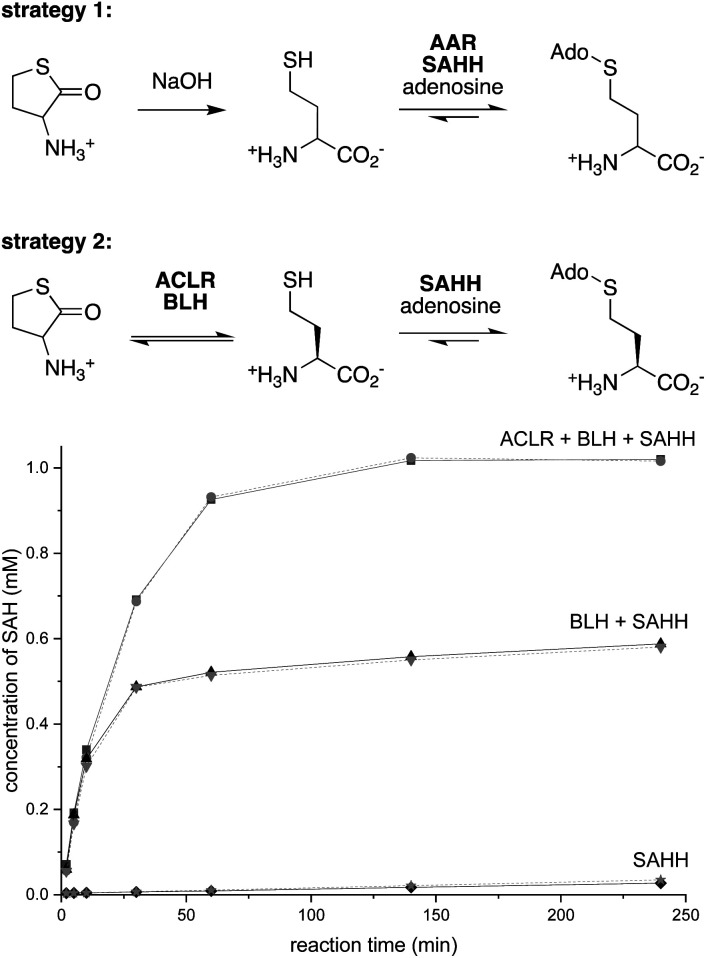
Top: strategies 1 and 2 for producing SAH from racemic Hcy thiolactone by dynamic kinetic resolution. Bottom: conversion of 1 mM racemic Hcy thiolactone and 1 mM adenosine to SAH by the combined activity of 1 μM *Ao*ACLR, 1 μM *Sc*BLH and 1 μM *Pa*SAHH (black) or *Mm*SAHH (gray) in phosphate buffer pH 8.0 at 25 °C.

Dynamic kinetic resolution is a powerful approach to convert racemic substrates into optically enriched products.^35,36^ Applying this concept to SAH production, we combined a pyridoxal-5′-phosphate (PLP)-dependent amino acid racemase (AAR) with SAHH, expecting that this cascade would convert racemic Hcy to SAH (Fig. 2). To enable this experiment, we produced the broad specificity amino acid racemase from *Vibrio cholerae* (*Vc*AAR, PDB code: 4BEU, EC 5.1.1.10)^37^ and SAHHs from *Pseudomonas aeruginosa* (*Pa*SAHH, PDB code: 6F3M)^38^ and from *Mus musculus* (*Mm*SAHH, PDB code: 5AXA) in *Escherichia coli*. The recombinant proteins were purified by Ni^II^-NTA affinity chromatography following standard protocols. The homogeneity of all proteins discussed in this report was assessed by SDS-PAGE (Fig. S1†).

Racemic Hcy was generated by hydrolysis of racemic Hcy thiolactone under basic conditions (Fig. S2†). The ability of *Vc*AAR and *Pa*SAHH or *Mm*SAHH to produce SAH in a phosphate buffer pH 8.0 at 25 °C was assessed by ion-exchange liquid chromatography (Fig. S3†). This analysis showed that the two-enzyme cascades convert all racemic Hcy thiolactone to SAH within 16 h. But there is a catch, however: ^1^H NMR characterization of the produced SAH revealed that *Vc*AAR not only racemizes Hcy but also epimerizes SAH (Fig. S5 and S6†), suggesting that optically pure SAH may not be obtained with this procedure. A second broad specificity racemase (AAR from *Pseudomonas putida*, *Pp*AAR, PDB code: 4DYJ)^39^ sharing 51% sequence identity with *Vc*AAR also epimerizes SAH, suggesting this to be a general flaw of this approach (Fig. S7 and S8†). Furthermore, after analyzing reactions containing l-, d- or racemic Hcy we found that SAHH also produces *S*-adenosyl-d-homocysteine (d-SAH) from d-Hcy (Fig. S9 and S10†). Although the initial production rates for d-SAH and l-SAH differ by 100-fold, this promiscuity is an additional source of stereochemical heterogeneity that may be difficult to suppress.

### Strategy 2

To avoid these problems, we envisioned an alternative approach in which racemization would precede stereoselective hydrolysis (Fig. 2). We found no literature describing a Hcy thiolactone racemase. However, given the activity of PLP-dependent α-amino-ε-caprolactam racemases (ACLR, EC 5.1.1.15) towards α-amino-lactams and α-amino acid amides,^35,40–43^ we surmised that such enzymes may also act on Hcy thiolactone. Consistent with this expectation, we found that the recombinant ACLR from *Achromobacter obae* (*Ao*ACLR, PDB code: 3DXW)^44^ affords efficient H/D exchange at Cα of racemic Hcy thiolactone (Fig. S11†). α-Ketobutyrate was not detected, even after 4 h, suggesting that enzyme-catalyzed γ-elimination – a potential side reactivity of PLP-activated Hcy thiolactone – occurs at a negligible rate. Importantly, no *Ao*ACLR-mediated H/D exchange was observed at Cα of SAH (Fig. S12†).

To exploit this racemase activity for SAH production, we combined *Ao*ACLR with SAHH and bleomycin hydrolase from *Saccharomyces cerevisiae* (*Sc*BLH, PDB code: 1GCB).^45^ This cysteine protease is named for its ability to hydrolyze the anticancer drug bleomycin (EC 3.4.22.40), but more likely has evolved to hydrolyze l-Hcy thiolactone as a protection from the toxic effects of this electrophile.^46,47^ Reactions containing 1 μM of each *Ao*ACLR, *Sc*BLH and *Pa*SAHH or *Mm*SAHH, 1 mM racemic Hcy thiolactone, 1 mM adenosine in 50 mM sodium phosphate buffer (pH 8.0) produced more than 0.9 mM SAH within the first hour with an initial rate of 33 μM min^−1^ (Fig. 2 and S13†). In the absence of *Ao*ACLR, the rate of SAH production slows down 100-fold after 50% conversion, consistent with the expected selectivity of *Sc*BLH for l-Hcy thiolactone.^46,47^ In the absence of *Ao*ACLR and *Sc*BLH, SAH production is equally slow and depends on uncatalyzed hydrolysis of Hcy thiolactone (*t*_1/2,hydrolysis_ = ∼24–30 h at pH = 7.4).^48^ To demonstrate, that this three-enzyme cascade can be used for preparative purposes, we extracted SAH from a 50 mL reaction initially containing 10 mM racemic Hcy thiolactone and adenosine. This procedure yielded 217 mg of SAH isolated as a trifluoroacetate salt as inferred by ^1^H/^19^F/^13^C NMR and HR-ESI-MS analysis (78% isolated yield, Fig. S14–S20†).

**Scheme 1:**
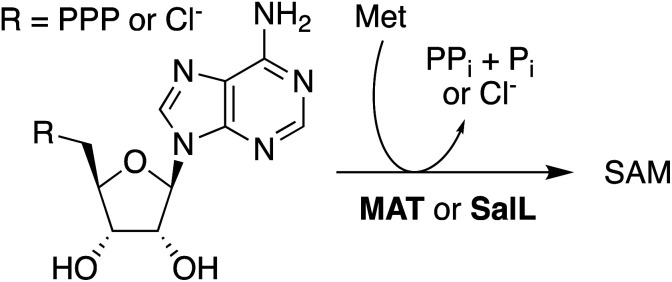
MAT- or SalL-catalyzed production of SAM from Met ant ATP or 5′-chloro-5′-deoxyadenosine.

*In situ*-generated SAH provides an excellent substrate for the production of SAM. Solutions containing *Ao*ACLR, *Sc*BLH, SAHH, and 1 mM of SAH were supplemented with 2 mM MeOTs and 2 μM HMT from *Ustilago maydis* (uma). Monitoring these reactions by HPLC showed that all SAH is converted to SAM within less than one hour (Fig. S21†). Synthesis of SAM also proceeds quantitatively starting with 10 mM adenosine and racemic Hcy thiolactone (Fig. S22†). Importantly, control reactions with 2 mM MeOTs but without uma produced no adenosine for at least 4 h, suggesting that uncatalyzed *S*-methylation of Hcy by MeOTs is not efficient enough to induce SAH consumption by the reversible activity of SAHH (Fig. S21†). Based on these observations we conclude that the synthesis of SAM from adenosine, Hcy thiolactone, and MeOTs is more efficient than previously published protocols. Production of SAM from ATP and Met suffers from the product inhibition of methionine adenosyltransferase (MAT, EC 2.5.1.6) by SAM (*K*_i_ = 10 μM) and pyrophosphate (*K*_i_ = 400 μM),^49^ making high concentrations of additives necessary to drive preparative reactions to completion (Scheme 1).^50–55^ As an exception, MAT from *Methanocaldococcus jannaschii* is characterized by comparatively weak product inhibition with respect to methionine and ATP (*K*_i_ near 2 mM).^56^ Alternatively, the enzyme SalL (EC 2.5.1.94) has been used to prepare SAM from 5′-chloro-5′-deoxyadenosine, but the efficiency of these reactions depends on many-fold excess of Met and low salt concentrations (Scheme 1).^57–61^

**Scheme 2:**
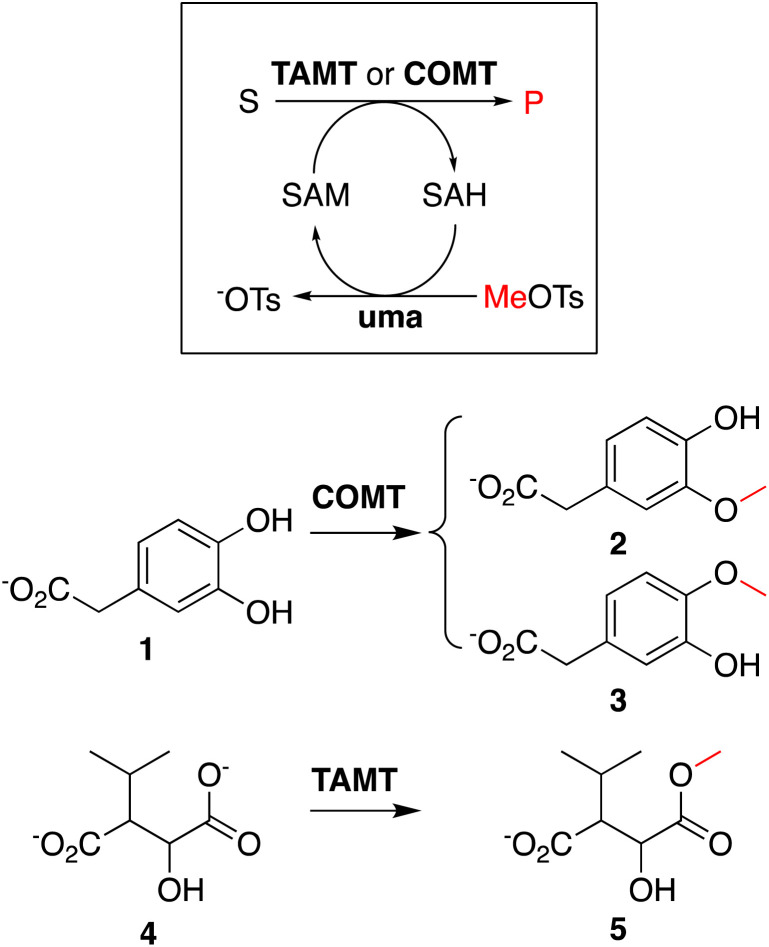
Enzyme cascades using *in situ*-generated SAH as methyl transfer catalyst: catechol MT (COMT)-catalyzed methylation of catechol (1) and *trans*-aconitate MT (TAMT)-catalyzed methylation of isopropyl maleate (4).

To showcase that *in situ* generated SAH can be used to initiate methyltransferase biocatalysis,^14^ we analyzed two methylation cascade reactions containing 5 μM uma and 5 μM of catechol MT (COMT) or *trans*-aconitate MT (TAMT) (Scheme 2), 2 mM of the requisite acceptor substrates 3,4-dihydroxyphenylacetic acid (1, Scheme 2) or 3-isopropyl malate (3), 40 μM *in situ* generated SAH, and 50 mM sodium phosphate buffer (pH 8.0) after incubated at 25 °C for 24 h. The ^1^H NMR spectra recorded for these solutions indicate quantitative methylation of 1 to 2 (87%) or 3 (13%, Fig. S23†), and 4 to 5 (Fig. S24†), matching previously reported results from similar transformations using commercial SAH.^15^

### SAH derivatives

With a final set of experiments, we explored the potential of the ACLR-BLH-SAHH cascade to generate artificial cofactors.^26,62–64^ Many derivatives of SAM and SAH have been designed as competitive methyltransferase inhibitors,^65–67^ as cofactors that are more stable towards uncatalyzed degradation,^22,24,61,68,69^ as cofactors transferring alternative alkyl groups,^16,17,20,59,70,71^ or as molecular hooks for fish for unknown MTs or unknown MT substrates.^72–75^ Despite the long list of *S*-nucleosyl Met derivatives known to be recognized by some MTs,^76–79^ predicting which cofactor modifications a particular MT might tolerate, remains difficult. This difficulty is related to the generally poor conservation of the SAM-binding pocket across the MT superfamily.^80–82^ Therefore, rapid access to a diverse range of *S*-nucleosyl Hcy and *S*-nucleosyl Met derivatives would significantly enhance screening efforts to align artificial cofactors with specific MTs.

**Fig. 3:**
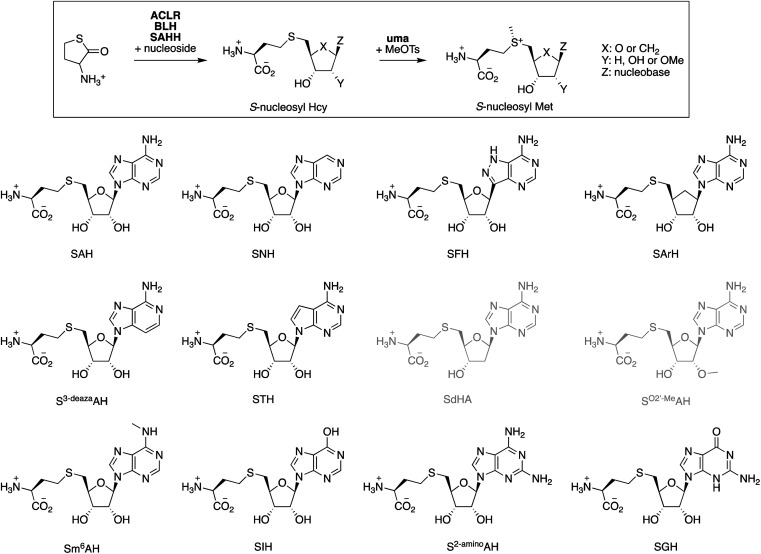
*S*-Nucleosyl Hcy derivatives produced by the ACLR-BLH-SAHH cascade reactions containing 1.05 mM racemic Hcy thiolactone, 1 mM adenosine derivatives, 1 μM *Ao*ACLR, 1 μM ScBLH, 1–20 μM *Pa*SAHH or *Mm*SAHH in 50 mM sodium phosphate buffer (pH 8.0). Compounds SdAH and S^O2′-Me^AH were not produced under these conditions (gray).

To test this idea, we screened eleven commercially available purine nucleosides (Fig. 3) as substrates for ACLR-BLH-SAHH-uma mediated production of *S*-nucleosyl Hcy and *S*-nucleosyl Met analogs. We incubated these nucleosides (1 mM) together and racemic Hcy thiolactone (1.05 mM) with *Ao*ACLR, *Sc*BLH, and PaSAHH or *Mm*SAHH in 50 mM phosphate buffer, pH 8.0 at 25 °C. The resulting nucleoside conjugates were analyzed by HPLC (quantification by UV at 265 nm), and identified by HR-ESI-MS and ^1^H/^13^C NMR (Fig. S25–S81†). Seven *S*-nucleosyl Hcy derivatives were formed with at least 90% conversion (Table 1), even though the initial rates for SAHH-catalyzed condensation vary by 400-fold. Two additional conjugates (STH, SGH) were also produced, but at least 4000-fold more slowly than SAH. The conjugates SdAH and S^O2′-Me^AH were not observed (grey, Fig. 3), consistent with the expectation that a free 2′-hydroxyl group at the ribose moiety is critical for recognition by SAHH and other nucleoside-binding proteins.^80,82,86^

**Table 1:** Evaluation of *S*-nucleosyl homocysteines as SAHH products and HMT substrates^a^

Compound	Full name	Yield^b^ % after time (h) [*Pa*SAHH, μM]	*k*_obs,condensation_ c (min^−1^)	Yield^c^ % after time (h) [*Mm*SAHH, μM]	*k*_obs,condensation_ d (min^−1^)	*k*_obs,S-methylation_ e (min^−1^)	*t*_1/2_ f	Ref.^g^
SAH	SAH	>98 (2) [1]	39 ± 1	>98 (2) [1]	37 ± 1	20 ± 1	33 ± 1	
SNH	*S*-Nebularyl-Hcy	96 (4) [5]	16 ± 1	95 (4) [5]	2.9 ± 0.1	8.3 ± 0.3	35 ± 1	83
SFH	*S*-Formycinyl-Hcy	>98 (4) [5]	9.1 ± 0.5	>98 (4) [5]	5.6 ± 0.1	8.9 ± 0.3	154 ± 11	83
SArH	*S*-Aristeromycinyl-Hcy	96 (20) [20]	0.1 ± 0.01	96 (20) [20]	0.065 ± 0.001	12 ± 1	424 ± 33	
S^3-deaza^AH	*S*-3-Deazaadenosyl-Hcy	>98 (1) [5]	33 ± 1	>98 (1) [20]	31 ± 2	11 ± 1	24 ± 1	83
STH	*S*-Tubercidinyl-Hcy	72 (44) [20]	0.0020 ± 0.0002	11 (44) [20]	0.0048 ± 0.0001	n.d.	n.d.	79
SdAH	*S*-Deoxyadenosyl-Hcy	<1 (20) [5]	n.a.	<1 (20) [5]	n.a.	n.d.	n.d.	
S^O2′-Me^AH	*S*-(2′-*O*-Methyladenosyl)-Hcy	<1 (20) [5]	n.a.	<1 (20) [5]	n.a.	n.d.	n.d.	
Sm^6^AH	*S*-N6-Methyladenosyl-Hcy	29 (20) [5]	0.15 ± 0.01	98 (20) [5]	4.7 ± 0.1	10 ± 1	37 ± 1	
SIH	*S*-Inosyl-Hcy	89 (2) [5]	3.2 ± 0.1	91 (2) [5]	5.3 ± 0.1	0.41 ± 0.02	n.d.	79, 84 and 85
S^2-amino^AH	*S*-2-Aminoadenosyl-Hcy	94 (2) [5]	10 ± 1	94 (2) [5]	12 ± 1	14 ± 1	60 ± 1	
SGH	*S*-Guanosyl-Hcy	36 (44) [20]	0.0090 ± 0.0002	48 (44) [20]	0.02 ± 0.001	n.d.	n.d.	

a Analytical data for the *S*-nucleosyl Hcy and *S*-nucleosyl Met analogs reported in this table are shown in the ESI (Fig. S37–S99).

b Reaction conditions: 200 μL solutions containing 1.05 mM racemic Hcy thiolactone, 1 mM nucleoside, 1 μM *Ao*ACLR, 1 μM *Sc*BLH, *Pa*SAHH or *Mm*SAHH (concentrations indicated in Table), and 50 mM sodium phosphate buffer (pH 8.0) were incubated at 25 °C (incubation time indicated in Table).

c Yield: % conversion of nucleoside to *S*-nucleosyl Hcy was quantified by HPLC at 265 nm (Fig. S25–S36).

d Initial rates for the condensation reactions (*k*_obs,condensation_) were determined by quantifying the time-dependent production of *S*-nucleosyl Hcy.

e Initial rates for the uma-catalyzed methylation of *S*-nucleosyl Hcy analogs (*k*_obs,*S*-methylation_) were determined by quantifying the time-dependent production of *S*-nucleosyl Met analogs. Reaction conditions: 200 μL solutions containing 0.5 mM *S*-nucleosyl Hcy, 1 mM MeOTs, 2 μM uma, and 50 mM sodium phosphate buffer, pH 8.0 were incubated at 25 °C.

f The concentrations of *S*-nucleosyl Met in 50 mM sodium phosphate buffer, pH 8.0 at 25 °C were monitored over 100 h. For comparison: *t*_1/2_ for SAM in 100 mM Tris–HCl, pH 8.0 at 37 °C has been measured to be 16 h.^68^

g References are given for *S*-nucleosyl Hcy analogs that have been made before using SAHH catalysis.

In a second step, we incubated seven *S*-nucleosyl Hcy analogs (0.5 mM) with MeOTs (1 mM) to test whether these compounds are substrate to uma-catalyzed *S*-methylation (Table 1 and Fig. S92–S99†). Remarkably, six of these compounds are methylated at similar rates as SAH – within a factor of three, suggesting that these *S*-nucleosyl Hcy analogs may be used as methyl transfer catalysts in methylation cascades. Of particular interest are the compounds SFH and SArH. HPLC-monitoring the concentration of these *S*-nucleosyl Met analogs in phosphate buffer, pH 8.0 at 25 °C for several days showed that methylated SFH and SArH are 5 – 10-fold more stable than SAM and other sulfonium derivatives in this study (Fig. S100†).

## Conclusions

Responding to the emerging role of SAH and SAM as reagents in biocatalysis,^14,25,26,29^ we have developed a protocol for their production from adenosine and racemic Hcy thiolactone in a scalable one-pot process that is not inhibited by its products. We have shown that *in situ* generated SAH can be used directly as cofactor-supplement for preparative methylation reactions, thereby reducing potential costs associated with scaling up. Our results also suggest that uma-catalyzed *S*-methylation of *in situ* generated SAH provides more efficient access to optically pure SAM than previously explored avenues. Finally, we used this protocol for the synthesis characterization of several *S*-nucleosyl Hcy and *S*-nucleosyl Met analogs as a step toward the development of artificial alkyl transfer cofactors.^87^ Our screen of eleven commercially available nucleosides identified two *S*-nucleosyl Hcy analogs as excellent substrates for enzyme-catalyzed *S*-methylation. The resulting *S*-nucleosyl Met analogs exhibit up to 10-fold greater stability than SAM, introducing two new candidates in the search for the most suitable artificial cofactor for MT biocatalysis.^22,24,61,68,69^

## Data availability

Electronic (ESI)† available: detailed descriptions of all experiments, protocols for the production and characterization of recombinant proteins, protocols for the synthesis and characterization of small molecules, kinetic data are reported in the ESI Fig. S1–S100.†

## Author contributions

X. W. and F. P. S. conceived the project and designed experiments. V. L. conducted all experimental work on “Strategy 1”, X. W. conducted all remaining experiments. X. W., V. L., and F. P. S. interpreted the results. X. W. and F. P. S. wrote the manuscript.

## Conflicts of interest

The authors declare no competing interests.

## Supplementary Material

SC-OLF-D4SC03801K-s001
