# Supplementary material for: Enzymatic synthesis of S-adenosyl-l-homocysteine and its nucleoside analogs from racemic homocysteine thiolactone
Source: Chem Sci. 2024 Sep 6;15(38):15900–6. doi: 10.1039/d4sc03801k (PMC11391342; doi:10.1039/d4sc03801k)
Supplement: SC-015-D4SC03801K-s001 [file SC-015-D4SC03801K-s001.pdf]

Supplementary Information (SI) for Chemical Science.
This journal is © The Royal Society of Chemistry 2024

Supporting Information

Enzymatic synthesis of S-adenosyl-L-homocysteine and its nucleoside analogs from racemic homocysteine thiolactone

Xiaojin Wen[1,2], Viviane Leopold[1], and Florian P. Seebeck[1,2]*

[1] Department of Chemistry, University of Basel, Mattenstrasse 22, Basel 4002, Switzerland

[2] Molecular Systems Engineering, National Competence Center in Research (NCCR), 4058 Basel, Switzerland

1. Materials and Methods

A. Unless otherwise stated, all chemicals and reagents were purchased from Sigma-Aldrich and used as received. Nebularine and N^6-methyladenosine were purchased from Cayman Chemical. Aristeromycin was purchased from Toronto Research Chemicals. Tubercidin was purchased from Combi-Blocks. 2'-*O*-Methyladenosine was purchased from Alfa Aesar. 2-Aminoadenosine was purchased from Fluorochem. Antibiotics for protein expression were purchased from PanReac AppliChem. Isopropyl β-D-thiogalactoside (IPTG) was purchased from Acros Organics. Roti®garose-His/Ni beads were purchased from ROTH (Art. # 1308.2). 2'-Deoxyadenosine hydrate, inosine, guanosine and buffer ingredients were purchased from Acros Organics. Deuterated solvents were purchased from Cambridge Isotope Laboratories.

B. High-performance liquid chromatography (HPLC) traces were obtained as follows (unless stated otherwise). 10 µL reaction samples were quenched by the addition of 10 µL of 1% trifluoroacetic acid (TFA) in water. 5 µL of quenched sample was analyzed by cation-exchange HPLC. HPLC traces for enzymatic reactions were recorded on a Shimadzu Prominence HPLC using a cation-exchange LC column 150 × 4.6 mm (Luna 5 µm SCX 100 Å) under the buffer A: 20 mM H_3PO_4; B: 20 mM H_3PO_4, 1 M NaCl, pH 2, filtered with 0.22 µm MCE Membrane MF-Millipore™. The HPLC gradient was programmed as follows: 0-0.5 min, 10 % B; 0.5-9 min, gradient from 10 to 40 % B; 9-11 min, gradient from 40 to 99 % B; 11-13.5 min, 99 % B; 13.5-14.5 min, gradient from 99 to 10 % B; 14.5-17 min, 10 % B. The flow rate was set to 1 mL/min. The HPLC traces were exported as ASCII files and processed on OriginLab2019b.

C. High-resolution electron spray ionization mass spectroscopy (HR-ESI-MS) spectra were obtained on a Bruker maXis 4G UHR-TOF Mass Spectrometer paired with a Shimadzu Nexera UPLC (X2 LC series). All nuclear magnetic resonance (NMR) spectra were recorded on a Bruker Avance Neo NMR spectrometer operating at 500 MHz proton frequency and chemical shifts were internally referenced to residual proton signals of solvents. Chemical shifts (δ) were reported in parts per million (ppm). Standard abbreviations indicating multiplicity were used as follows: s (singlet), d (doublet), t (triplet), dd (doublet of doublets), and m (multiplet). Coupling constants (*J*) were reported in Hertz (Hz).

2. Protein Sequences

>*Pp*AAR (PDB: 4DYJ, *Pseudomonas putida*)

MAFRRTLLAASLALLITGQAPLYAAPPLSMDNGTNALTVQNSNAWVEVSASALQHNIRTLQAELAGKSRLCAVLK
ADAYGHGIGLVMPSIIAQGVPCVAVASNEEARVVRASGFTGQLVRVRLASLSELEDALQYDMEELVGSAEFARQV
DAIAARHGKTLRIHMALNSSGMSRNGVEMATWSGRGEALQITDQKHLKLVALMTHFAVEDKDDVRKGLAAFNEQT
DWLIKHAKLDRSKLTLHAANSFATLEVPEARLDMVRTGGALFGDTVPARTEYQRAMQFKSHVAAVHSYPAGNTVG
YDRTFTLARDSRLANITVGYSDGYRRVFTNKGHVLINGHRIPVVGKVSMNTLMVDVTDFPDVKGGNEVVLFGKQA
GGEITQAEMEEINGALLADLYTVWGSSNPKILVDLEHHHHHH

>*Vc*AAR (PDB: 4BEU, *Vibrio cholerae*)

MAPLHIDTALPDAAQIQQSNSWLEISLGQFQSNIEQFKSHMNANTKICAIMKADAYGNGIRGLMPTIIAQGIPCV
GVASNAEARAVRESGFKGELIRVRSASLSEMSSALDLNIEELIGTHQQALDLAELAKQSGKTLKVHIALNDGGMG
RNGIDMTTEAGKKEAVSIATQPSLSVVGIMTHFPNYNADEVRAKLAQFKESSTWLMQQANLKREEITLHVANSYT
ALNVPEAQLDMVRPGGVLFGDLPTNPEYPSIVSFKTRVSSLHHLPKDSTVGYDSTFTTSRDSVLANLPVGYSDGY
PRKMGNKAEVLINGQRAKVVGVTSMNTTVVDVTEIKGVLPGQEVVLFGQQQKQSIAVSEMENNAELIFPELYTLW
GTSNPRFYVKSSGLEHHHHHH

>*Ao*ACLR (PDB: 3DXW, *Achromobacter obae*)

MGHHHHHHAENLYFQGSGSLVPRGSHMTKALYDRDGAAIGNLQKLRFFPLAISGGRGARLIEENGRELIDLSGAW
GAASLGYGHPAIVAAVSAAAANPAGATILSASNAPAVTLAERLLASFPGEGTHKIWFGHSGSDANEAAYRAIVKA
TGRSGVIAFAGAYHGCTVGSMAFSGHSVQADAAKADGLILLPYPDPYRPYRNDPTGDAILTLLTEKLAAVPAGSI
GAAFIEPIQSDGGLIVPPDGFLRKFADICRAHGILVVCDEVKVGLARSGRLHCFEHEGFVPDILVLGKGLGGGLP
LSAVIAPAEILDCASAFAMQTLHGNPISAAAGLAVLETIDRDDLPAMAERKGRLLRDGLSELAKRHPLIGDIRGR
GLACGMELVCDRQSREPARAETAKLIYRAYQLGLVVYYVGMNGNVLEFTPPLTITETDIHKALDLLDRAFSELSA
VSNEEIAQFAGW

> *Sc*BLH (PDB: 1GCB, *Saccharomyces cerevisiae*)

MGHHHHHHAENLYFQGSGSSSIDISKINSWNKEFQSDLTHQLATTVLKNYNADDALLNKTRLQKQDNRVFNTVVS
TDSTPVTNQKSSGRCWLFAATNQLRLNVLSELNLKEFELSQAYLFFYDKLEKANYFLDQIVSSADQDIDSRLVQY
LLAAPTEDGGQYSMFLNLVKKYGLIPKDLYGDLPYSTTASRKWNSLLTTKLREFAETLRTALKERSADDSIIVTL
REQMQREIFRLMSLFMDIPPVQPNEQFTWEYVDKDKKIHTIKSTPLEFASKYAKLDPSTPVSLINDPRHPYGKLI
KIDRLGNVLGGDAVIYLNVDNETLSKLVVKRLQNNKAVFFGSHTPKFMDKKTGVMDIELWNYPAIGYNLPQQKAS
RIRYHESLMTHAMLITGCHVDETSKLPLRYRVENSWGKDSGKDGLYVMTQKYFEEYCFQIVVDINELPKELASKF
TSGKEEPIVLPIWDPMGALAK

>*Mm*SAHH (PDB: 5AXA, *Mus musculus*)

MGSSHHHHHHSSGLVPRGSHMSDKLPYKVADIGLAAWGRKALDIAENEMPGLMRMREMYSASKPLKGARIAGCLH
MTVETAVLIETLVALGAEVRWSSCNIFSTQDHAAAAIAKAGIPVFAWKGETDEEYLWCIEQTLHFKDGPLNMILD
DGGDLTNLIHTKYPQLLSGIRGISEETTTGVHNLYKMMSNGILKVPAINVNDSVTKSKFDNLYGCRESLIDGIKR
ATDVMIAGKVAVVAGYGDVGKGCAQALRGFGARVIITEIDPINALQAAMEGYEVTTMDEACKEGNIFVTTTGCVD
IILGRHFEQMKDDAIVCNIGHFDVEIDVKWLNENAVEKVNIKPQVDRYWLKNGRRIILLAEGRLVNLGCAMGHPS
FVMSNSFTNQVMAQIELWTHPDKYPVGVHFLPKKLDEAVAEAHLGKLNVKLTKLTEKQAQYLGMPINGPFKPDHY
RY

>*Pa*SAHH (PDB: 6F3M, *Pseudomonas aeruginosa PAO1*)

MGSSHHHHHHSSGLVPRGSHMSTVMTPAGFTDYKVADITLAAWGRRELIIAESEMPALMGLRRKYAGQQPLKGAK
ILGCIHMTIQTGVLIETLVALGAEVRWSSCNIFSTQDQAAAAIAAAGIPVFAWKGETEEEYEWCIEQTILKDGQP
WDANMVLDDGGDLTEILHKKYPQMLERIHGITEETTTGVHRLLDMLKNGTLKVPAINVNDSVTKSKNDNKYGCRH
SLNDAIKRGTDHLLSGKQALVIGYGDVGKGSSQSLRQEGMIVKVAEVDPICAMQACMDGFEVVSPYKNGINDGTE
ASIDAALLGKIDLIVTTTGNVNVCDANMLKALKKRAVVCNIGHFDNEIDTAFMRKNWAWEEVKPQVHKIHRTGKD
GFDAHNDDYLILLAEGRLVNLGNATGHPSRIMDGSFANQVLAQIHLFEQKYADLPAAEKAKRLSVEVLPKKLDEE
VALEMVKGFGGVVTQLTPKQAEYIGVSVEGPFKPDTYRY

>uma (UniProtKB: A0A0D1DT00, *Ustilago maydis*)

MTSSLSKDDQIQNLRRLFADSGVPNDPKAWDQAWIDSTTPWDANRPQPALVELLEGAHDADAKVPDVDGNLIPVS
QAIPKGDGTAVVPGCGRGYDARVFAERGLTSYGVDISSNAVAAANKWLGDQDLPTELDDKVNFAEADFFTLGTSK
SLVLELSKPGQATLAYDYTFLCAIPPSLRTTWAETYTRLLAKHGVLIALVFPIHGDRPGGPPFSISPQLVRELLG
SQKNADGSAAWTELVELKPKGPETRPDVERMMVWRRSLEHHHHHH

>TAMT (UniProtKB: P32643, *Saccharomyces cerevisiae (strain ATCC 204508/S288c)*)

MSSTFSASDFNSERYSSSRPSYPSDFYKMIDEYHDGERKLLVDVGCGPGTATLQMAQELKPFEQIIGSDLSATMI
KTAEVIKEGSPDTYKNVSFKISSSDDFKFLGADSVDKQKIDMITAVECAHWFDFEKFQRSAYANLRKDGTIAIWG
YADPIFPDYPEFDDLMIEVPYGKQGLGPYWEQPGRSRLRNMLKDSHLDPELFHDIQVSYFCAEDVRDKVKLHQHT
KKPLLIRKQVTLVEFADYVRTWSAYHQWKQDPKNKDKEDVADWFIKESLRRRPELSTNTKIEVVWNTFYKLGKRV
LEHHHHHH

>COMT (UniProtKB: P21964, *Homo sapiens*)

MGHHHHHHENLYFQGDTKEQRILNHVLQHAEPGNAQSVLEAIDTYSEQKEWAMNVGDKKGKIVDAVIQEHQPSVL
LELGAYVGYSAVRMARLLSPGARLITIEINPDSAAITQRMVDFAGVKDKVTLVVGASQDIIPQLKKKYDVDTLDM
VFLDHWKDRYLPDTLLLEESGLLRKGTVLLADNVISPGAPDFLAHVRGSSRFEATHYQSFLEYREVVDGLEKAIY
KGPGSEAGP

Comments: The COMT we used here was a cysteine-free variant of the natural human COMT. The mutations were C33S, C69V, C95S, C157S, C173S, C188R and C191A (highlighted in red). N- and C-terminal tails appended to the recombinant proteins are highlighted in gray.

3. General Procedures

A. **Plasmids.** The plasmid coding for HMT uma was obtained from Prof. Dr. Stephan Hammer in Bielefeld, Germany.[1] The plasmids coding for *Mm*SAHH and *Pa*SAHH were obtained from Prof. Dr. Jennifer Andexer in Freiburg, Germany. Codon-optimized genes of all other enzymes were purchased from BioCat GmbH as inserts in pET28a(+) plasmids as NcoI/Xho1 fragments. The expression strain SAH nucleosidase deficient cell *E. coli* Δmtn (DE3) was constructed in a previous study in our lab.[2]

B. **Transformation.** Expression plasmids were transformed into *E. coli* Δmtn (DE3) cell by electroporation using a BIO-RAD GENE PULSER® II. The voltage was set to 2 kV, resulting in a time constant of around 5 msec. Capacitance was set at 30 µF. Immediately after applying the electric pulse, cells were suspended in 1 mL of 37°C Lysogeny Broth (LB). The cell culture was incubated at 37°C for a minimum of 45 minutes before plating on LB-Agar plates containing antibiotic kanamycin.

C. **Cell culture.** Cells containing the plasmid were collected from selective kanamycin containing LB-AGAR plates and used to inoculate the LB medium. After incubation at 37°C overnight, sterilized Terrific Broth (TB) containing 50 mg/L kanamycin was inoculated with 5 mL of pre-culture per L of medium. The 1 L of cultures were incubated in a 3 L shaking flask at 37°C (180 rpm) until OD_{600} reached 0.6 and then the cultures were allowed to cool down to 18°C by changing the set temperature of the shaker. The cultures were supplemented with 100 µM IPTG and incubated for 20 h more h at 18°C. Cells were harvested by centrifugation at 17,568 x g for 25 minutes at 4°C and stored at -20°C for a minimum of 2 hours before purification.

D. **Protein purification.** For purification, frozen cell pellets were suspended in 4 mL per gram of wet cells (ml/g_{cww}) of lysis buffer (50 mM sodium phosphate, 300 mM NaCl, pH 8.0). The suspension was homogenized at 4°C by EmulsiFlex at 1000 bar applied with 4 bar pressure of N_2. The cell-free lysates were cleared by centrifugation at 47,850 x g for 45 minutes at 4°C. The cleared lysate was supplemented with 1 mL of Ni^{II} NTA agarose slurry per 25 ml lysate and incubated at 4°C for 20 minutes. Subsequently, this suspension was loaded onto a column (10 mL polypropylene column, Thermo Scientific) with a matching polyethylene frit at the bottom. The agarose beads were washed with 10 mL cold lysis buffer containing 10 mM and 20 mM imidazole respectively. The protein was eluted in cold lysis buffer containing 250 mM imidazole. The absorbance at 280 nm of the eluted fractions was measured on NanoDrop 2000 Spectrophotometer (Thermo Scientific). Fractions with $Abs_{280nm} > 0.2$ were combined and dialyzed in a reconstituted cellulose bag (MW 12-14 kDa cutoff, 23 µm thick, art.# E684.1, purchased from ZelluTransROTH®) against dialysis buffer (50 mM sodium phosphate, pH 8.0). The final protein concentration was determined by measuring Abs_{280nm} against the dialysis buffer. The protein solution was aliquoted, frozen in liquid nitrogen, and stored at -80°C. The homogeneity of the purified enzymes was assessed by sodium dodecyl sulfate polyacrylamide gel electrophoresis (SDS-PAGE) under denaturing conditions. The gels were stained with Coomassie brilliant blue (Figure S1).

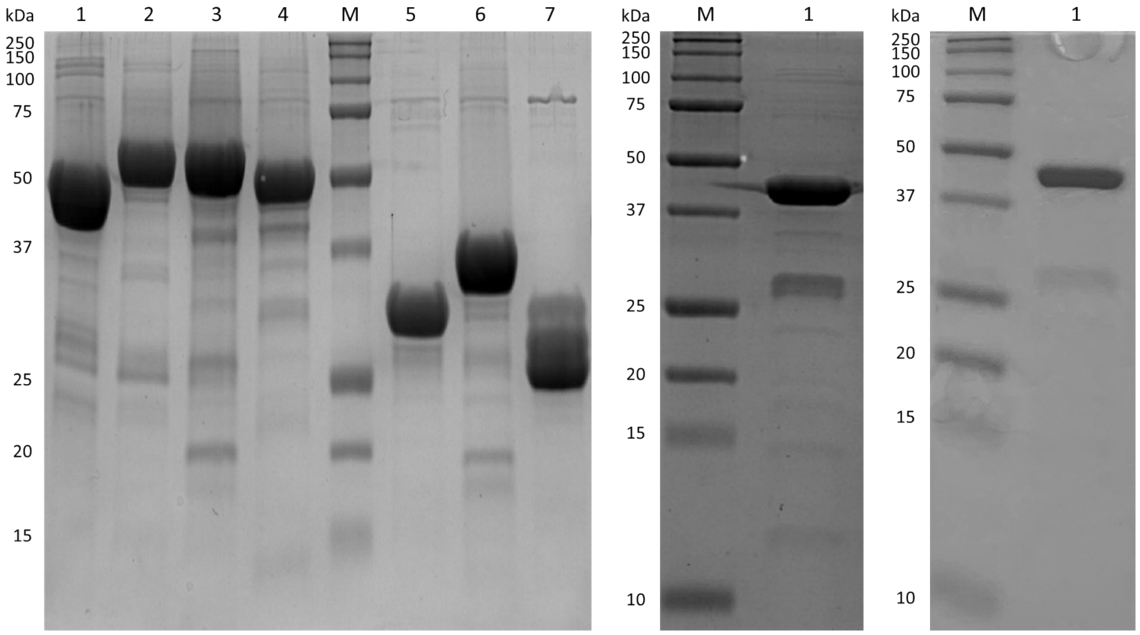

A **B** **C**

Figure S1. SDS-PAGE of recombinant enzymes. A) Lane M: marker with annotated MS, lane 1: *Ao*ACLR, lane 2: *Sc*BLH, lane 3: *Pa*SAHH, lane 4: *Mm*SAHH, lane 5: uma, lane 6: TAMT, lane 7: COMT; B) Lane M: marker with annotated MS, lane 1: *Vc*AAR; C) Lane M: marker with annotated MS, lane 1: *Pp*AAR.

Chemical synthesis of D/L-homocysteine (D/L-Hcy)

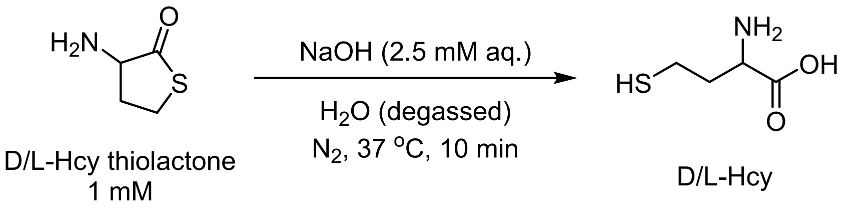

The synthesis of D/L-homocysteine (D/L-Hcy) was achieved under anaerobic environment. The conditions for ring opening of D/L-Hcy thiolactone follow the protocol established by Knipp *et al.*[3] Under anaerobic conditions, 1 mM of D/L-Hcy thiolactone·HCl was prepared in degassed milli-Q water and incubated with 2.5 mM NaOH prepared in degassed milli-Q water for 10 min at 37 °C, 300 rpm. Next, the pH was neutralized by the addition of H_3PO_4. The product was aliquoted and stored at -20 °C. The purity of D/L-Hcy was confirmed by ^{1}H-NMR.

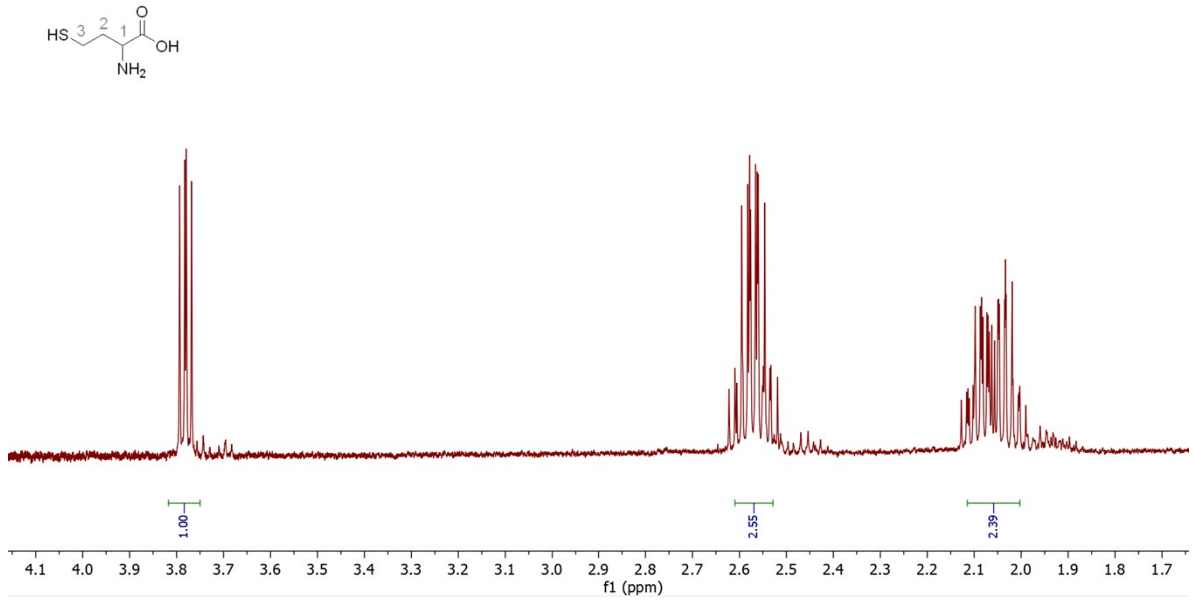

Figure S2. ^{1}H NMR spectrum of D/L-Hcy in D_2O. ^{1}H NMR (500 MHz, D_2O) δ 3.78 (dd, J = 7.2, J = 5.5 Hz, 1H, H-1), 2.63–2.50 (m, 2H, H-2), 2.14–1.98 (m, 2H, H-3).

Conversion of D/L-Hcy into SAH

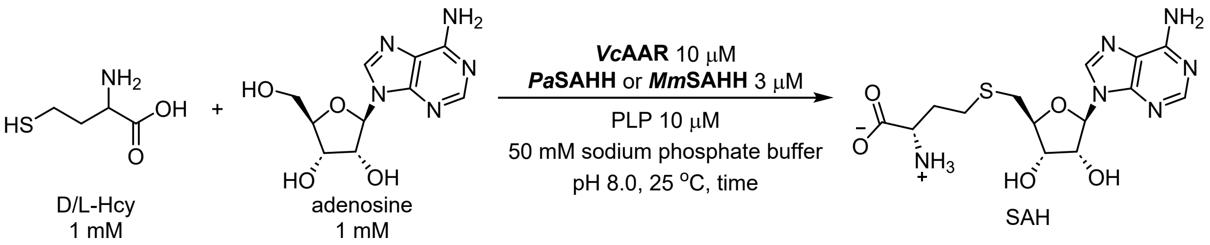

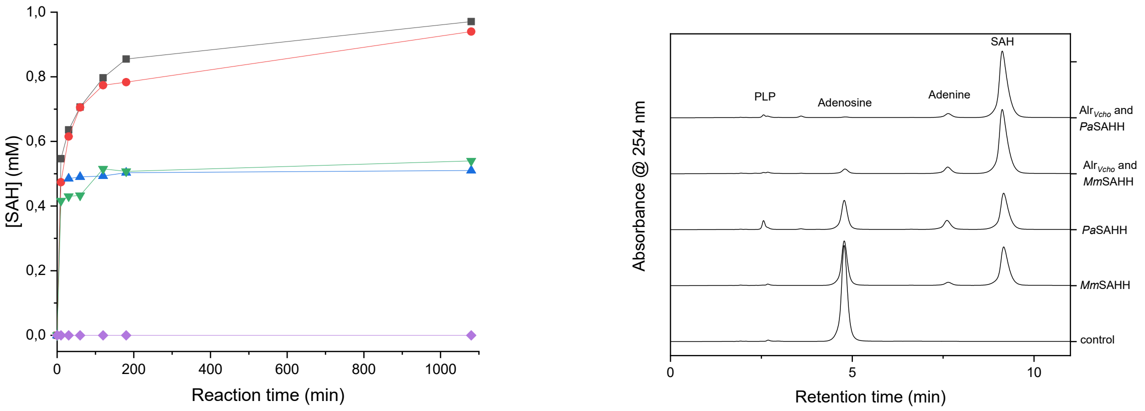

Figure S3. Left: Time-dependent production of SAH as monitored by cation-exchange HPLC at 254 nm. **Black curve:** reaction containing *Vc*AAR and *Pa*SAHH. **Red curve:** reaction containing *Vc*AAR and *Mm*SAHH. **Blue curve:** reaction containing only *Pa*SAHH. **Green curve:** reaction containing only *Mm*SAHH. **Purple curve**: no enzyme. **Right:** Representative IE-HPLC chromatograms recorded at 254 nm after overnight incubation of the reaction with *Vc*AAR and SAHH. Reaction conditions: 200 µL reactions containing 1 mM D/L-Hcy, 1 mM adenosine, 10 µM *Vc*AAR, 10 µM PLP, 3 µM SAHH (*Pa*SAHH or *Mm*SAHH) and 50 mM sodium phosphate buffer (pH 8.0) were incubated at 25 °C.

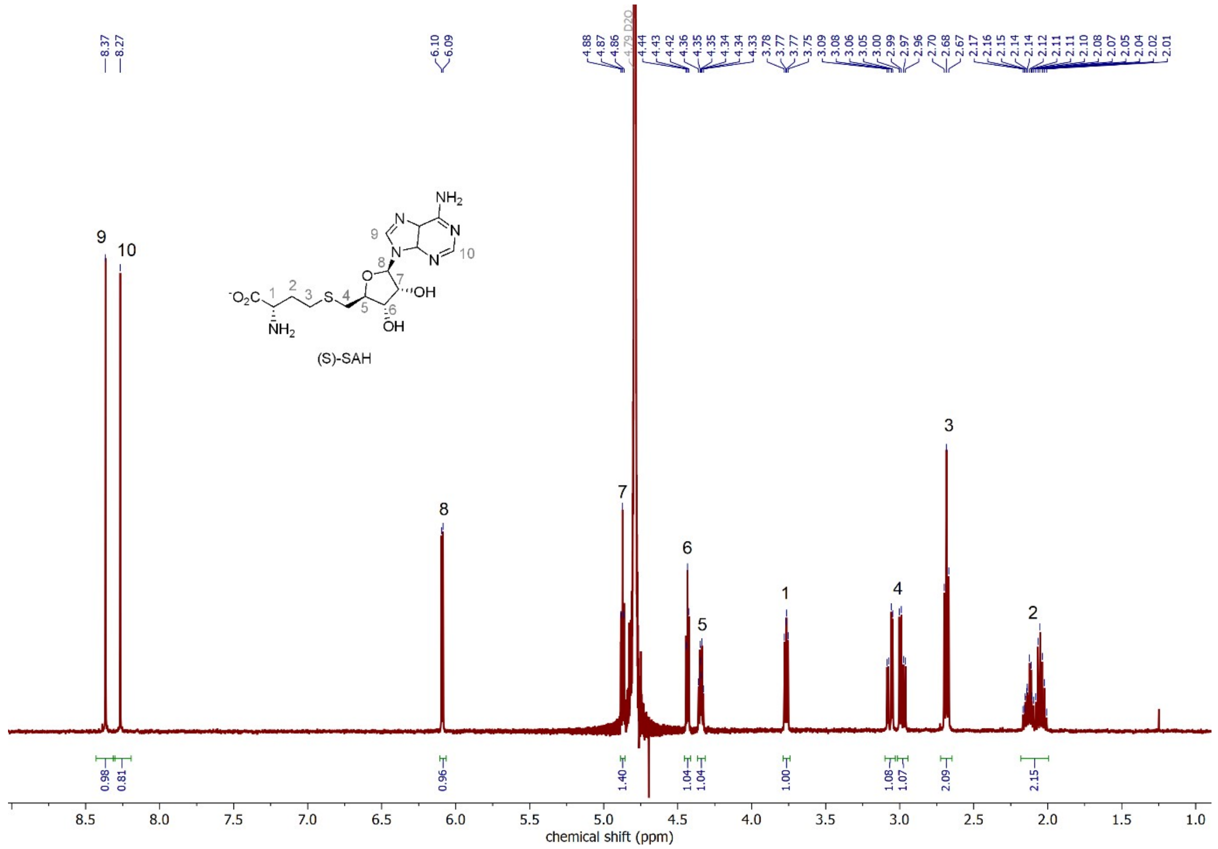

Figure S4. ^{1}H-NMR of commercial (*S*)-SAH in 50 mM sodium phosphate buffer (pD 8.4) in D_2O. ^{1}H NMR (500 MHz, D_2O) δ 8.37 (s, 1H, H-9), 8.27 (s, 1H, H-10), 6.09 (d, J = 5.2 Hz, 1H, H-8), 4.87 (t, J = 5.3 Hz, 1H, H-7), 4.43 (t, J = 5.2 Hz, 1H, H-6), 4.34 (dt, J = 6.7, 4.9 Hz, 1H, H-5), 3.77 (dd, J = 7.1, 5.5 Hz, 1H, H-1), 3.07 (dd, J = 14.2, 5.0 Hz, 1H, H-4a), 2.98 (dd, J = 14.2, 6.7 Hz, 1H, H-4b), 2.68 (t, J = 7.6 Hz, 2H, H-3), 2.19–1.99 (m, 2H, H-2).[4]

Racemization of Hcy and SAH by *Vc*AAR

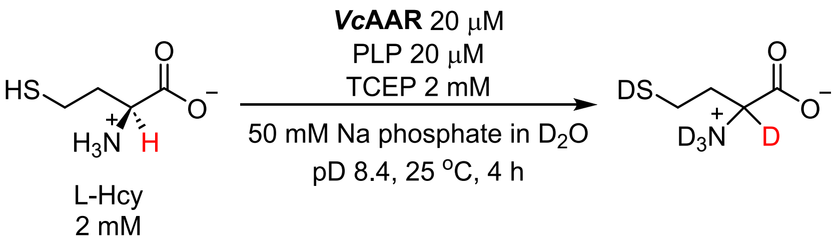

L-Hcy
2 mM

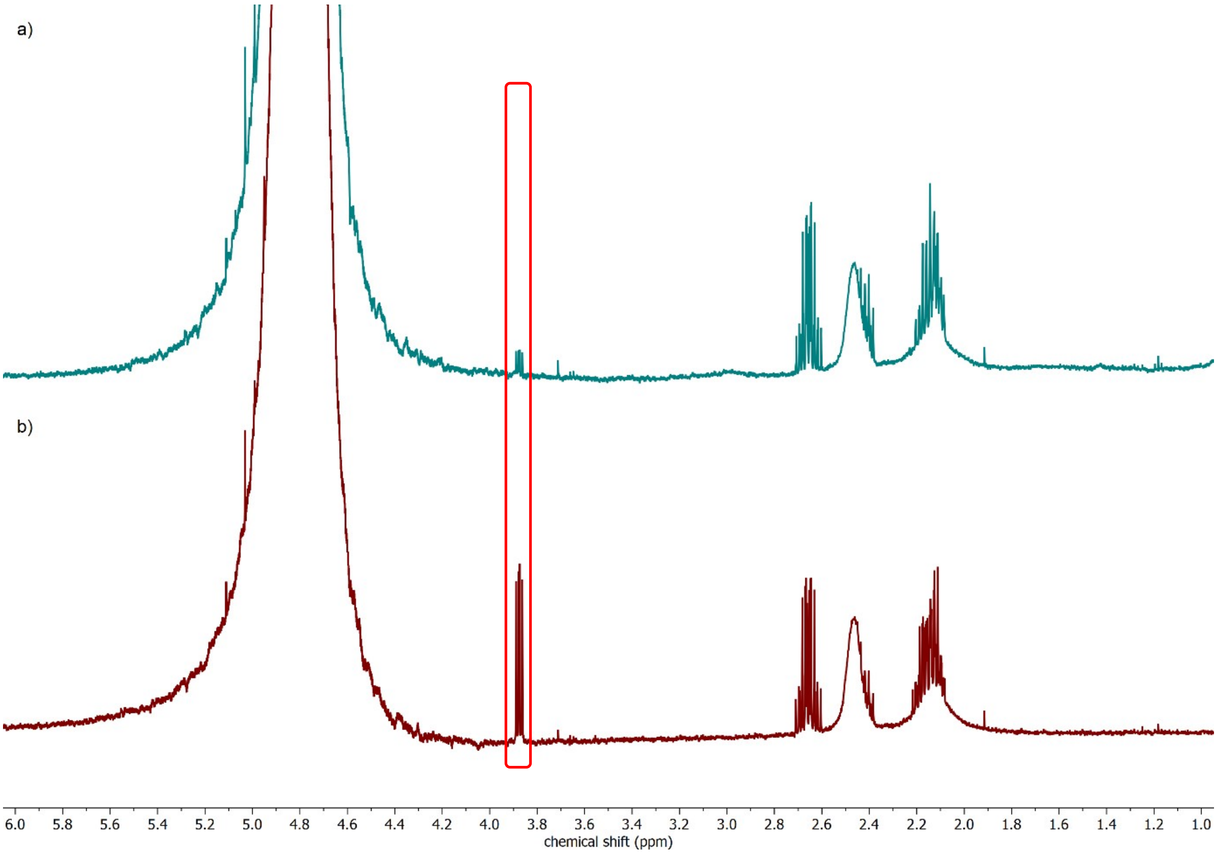

Figure S5. ^1H NMR spectra of racemization of L-Hcy with *Vc*AAR in D_2O. The α-H of L-Hcy (chemical shift 3.87 ppm) was labeled in the red rectangle. a) with *Vc*AAR and PLP; b) control: without *Vc*AAR and PLP. Reaction conditions: 0.5 mL reaction containing 2 mM L-Hcy, 2 mM TCEP, 20 μM *Vc*AAR, 20 μM PLP, and 50 mM sodium phosphate buffer in D_2O (pD 8.4) was incubated at room temperature. After 4 hours, the reaction was directly analyzed by ^1H NMR.

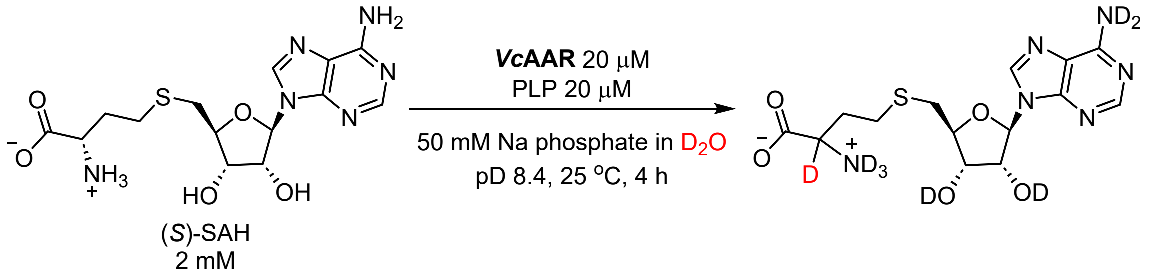

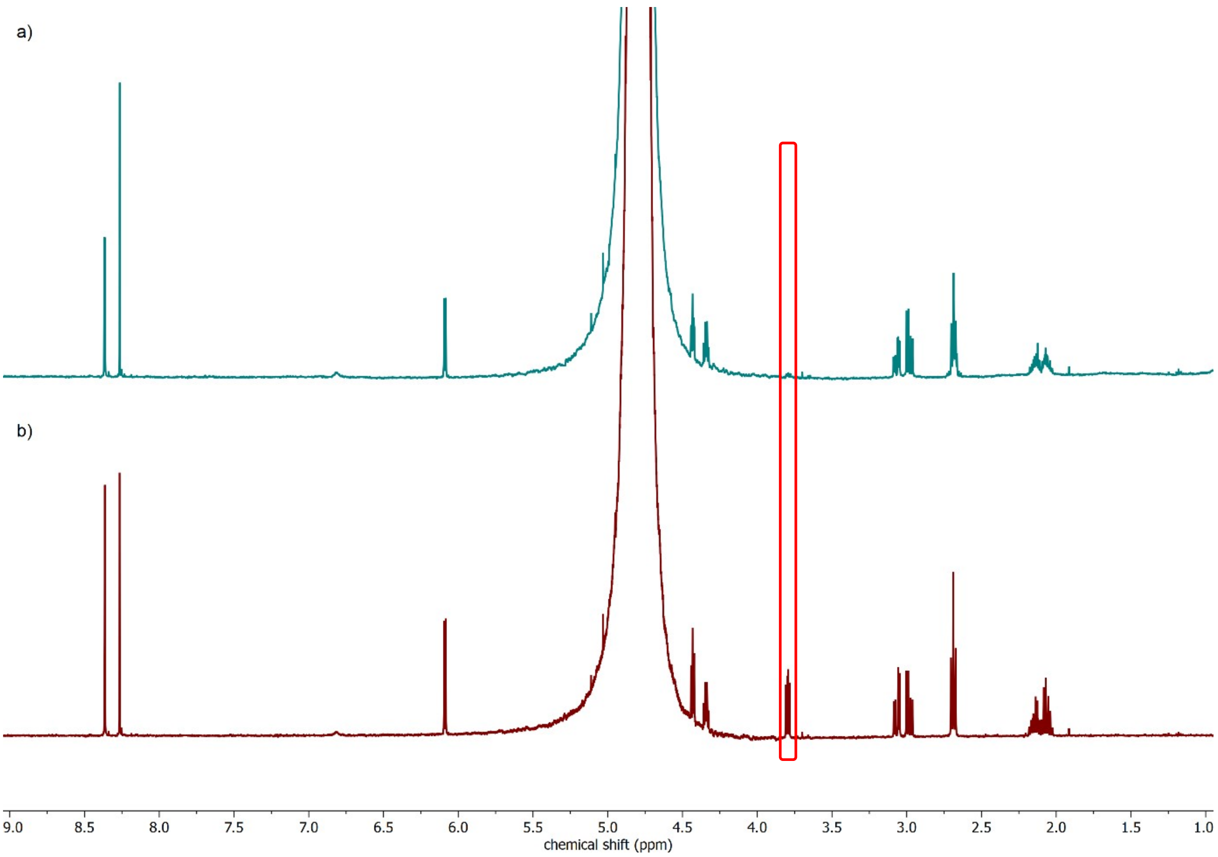

Figure S6. ^{1}H NMR spectra of racemization of (*S*)-SAH with *Vc*AAR in D_2O. The α-H of (*S*)-SAH (chemical shift 3.79 ppm) was labeled in the red rectangle. a) with *Vc*AAR and PLP; b) control: without *Vc*AAR and PLP. Reaction conditions: 0.5 mL reaction containing 2 mM (*S*)-SAH, 20 µM *Vc*AAR, 20 µM PLP, and 50 mM sodium phosphate buffer in D_2O (pD 8.4) was incubated at room temperature. After 4 hours, the reaction was directly analyzed by ^{1}H NMR.

Racemization of Hcy and SAH by *Pp*AAR

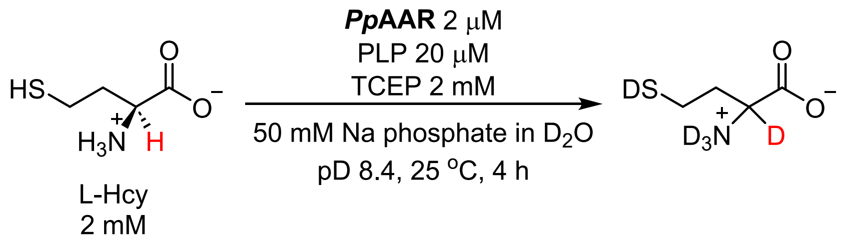

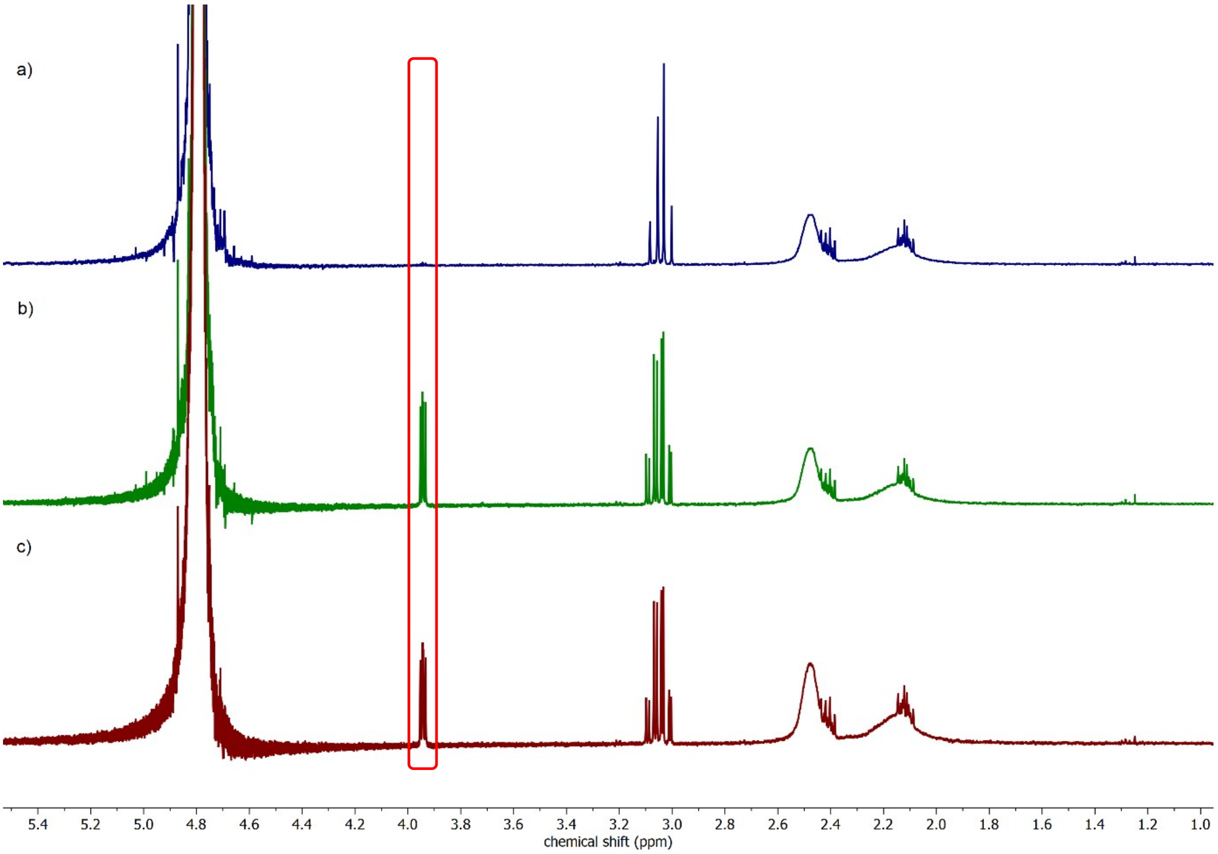

Figure S7. ^{1}H NMR spectra of racemization of L-Hcy with *Pp*AAR in D_2O. The α-H of L-Hcy (chemical shift 3.94 ppm) was labeled in the red rectangle. a) with *Pp*AAR and PLP; b) with only PLP; c) control: without *Pp*AAR and PLP. Reaction conditions: 0.5 mL reaction containing 2 mM L-Hcy, 2 mM TCEP, 2 μM *Pp*AAR, 20 μM PLP, and 50 mM sodium phosphate buffer in D_2O (pD 8.4) was incubated at room temperature. After 4 hours, the reaction was directly analyzed by ^{1}H NMR.

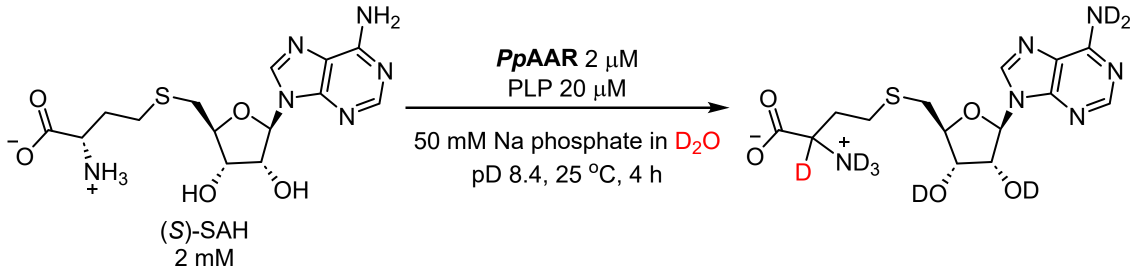

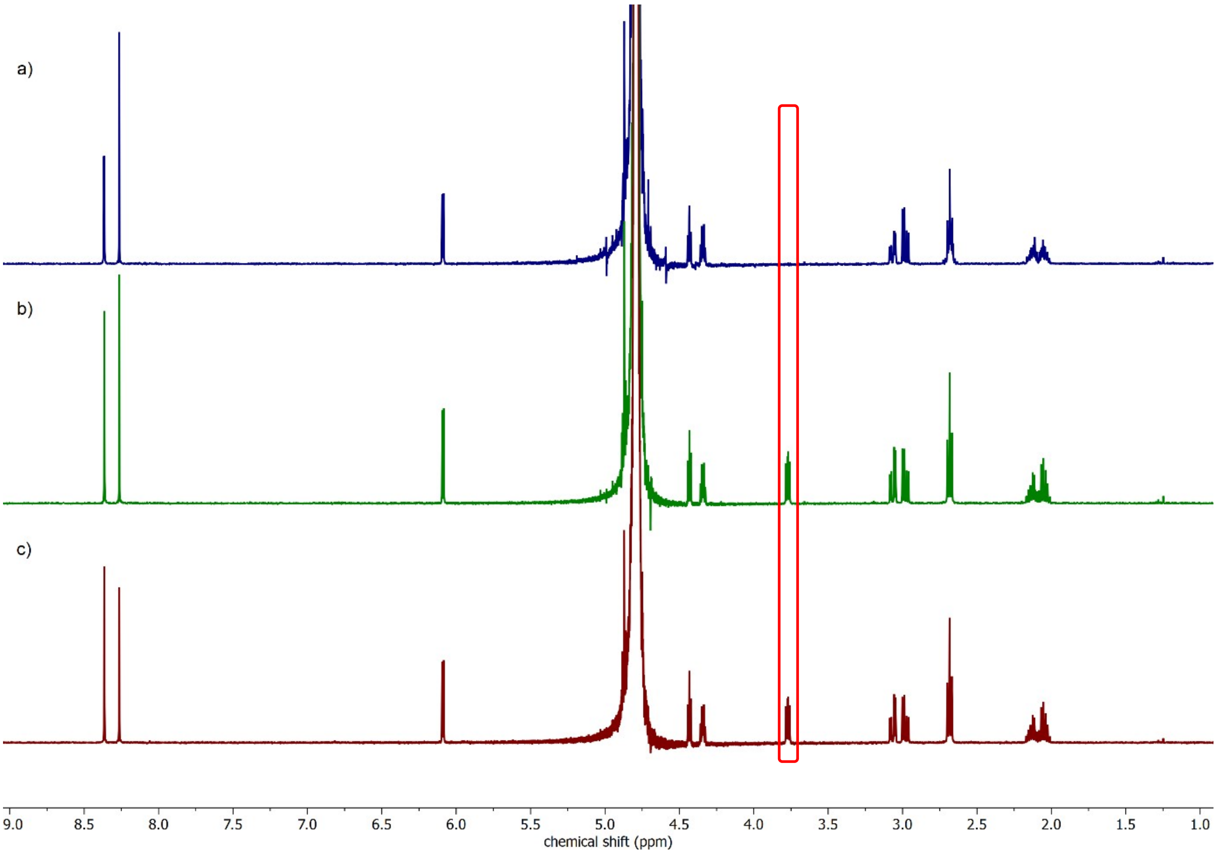

Figure S8. ^{1}H NMR spectra of racemization of (*S*)-SAH with *Pp*AAR in D_2O. The α-H of (*S*)-SAH (chemical shift 3.77 ppm) was labeled in the red rectangle. a) with *Pp*AAR and PLP; b) with only PLP; c) control: without *Pp*AAR and PLP. Reaction conditions: 0.5 mL reaction containing 2 mM (*S*)-SAH, 2 µM *Pp*AAR, 20 µM PLP, and 50 mM sodium phosphate buffer in D_2O (pD 8.4) was incubated at room temperature. After 4 hours, the reaction was directly analyzed by ^{1}H NMR.

SAH formation from L-, D-, or racemic Hcy catalyzed by SAHH

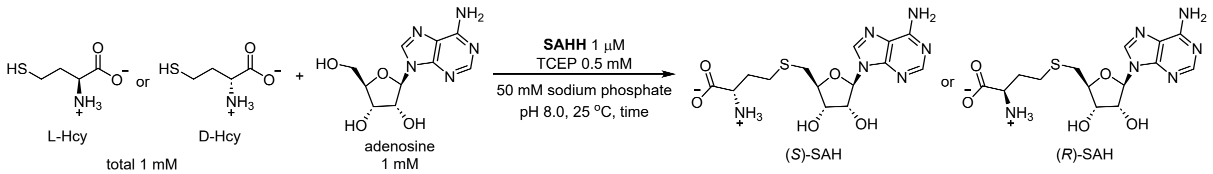

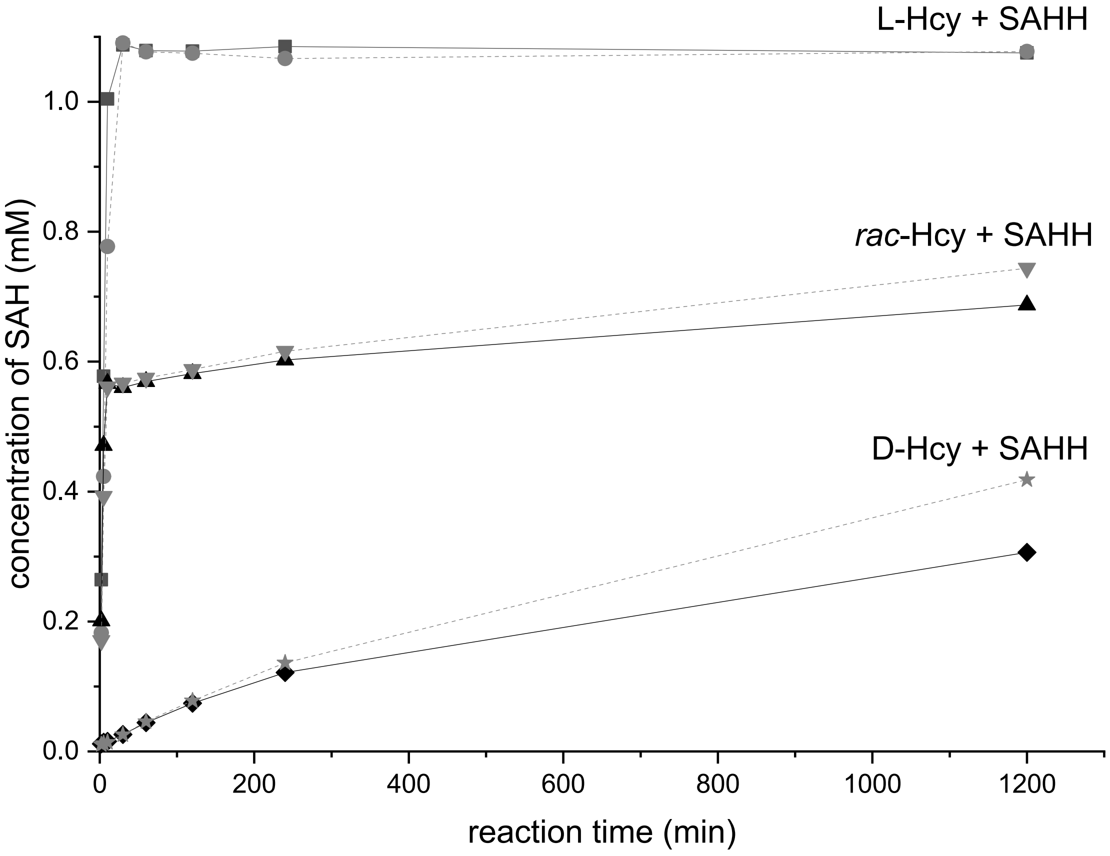

Figure S9. Time-dependent SAH formation from adenosine and two enantiomers of Hcy catalyzed by SAHH. The concentration of SAH was determined by HPLC. **Black solid**: with *Pa*SAHH; **Grey dash**: with *Mm*SAHH. Reaction conditions: 200 μL reaction containing 1 mM L-Hcy or 1 mM D-Hcy or racemic mixture of 0.5 mM L-Hcy and 0.5 mM D-Hcy, 1 mM adenosine, 0.5 mM TCEP, 1 μM *Pa*SAHH or *Mm*SAHH and 50 mM sodium phosphate buffer (pH 8.0) was incubated at 25 °C.

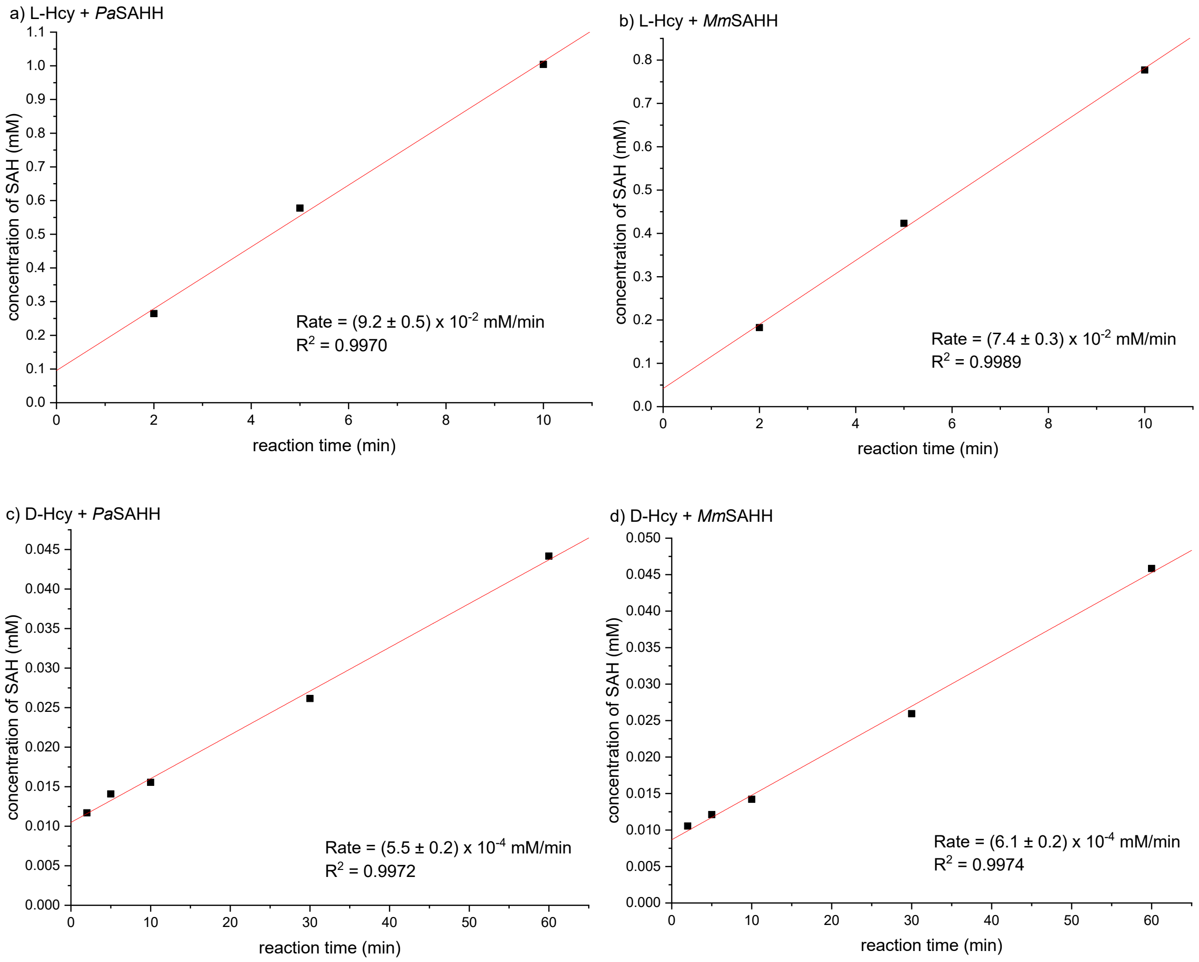

Figure S10. The initial rates of SAHH-catalyzed SAH production from adenosine and two enantiomers of Hcy. a) Starting from L-Hcy with *Pa*SAHH; b) Starting from L-Hcy with *Mm*SAHH; c) Starting from D-Hcy with *Pa*SAHH; d) Starting from D-Hcy with *Mm*SAHH. The data was obtained from Figure S9.

Racemization of Hcy thiolactone and SAH by *Ao*ACLR

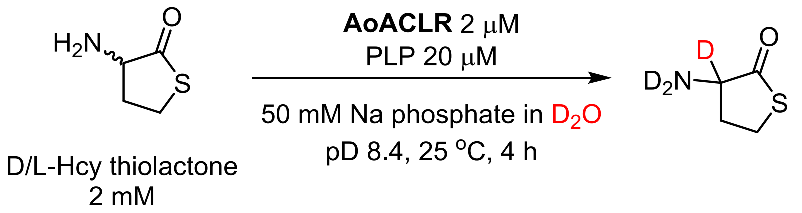

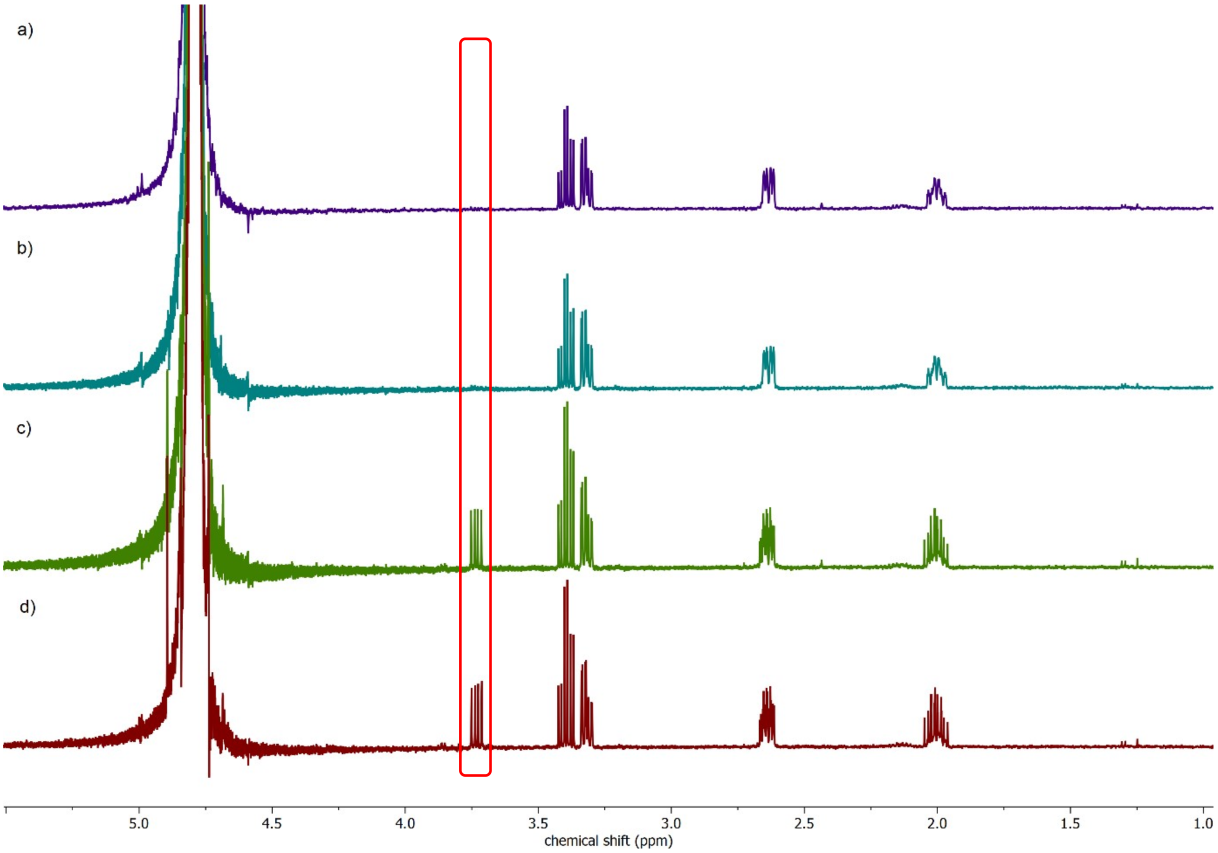

Figure S11. ^{1}H NMR spectra of racemization of D/L-Hcy thiolactone with *Ao*ACLR in D_2O. The α-H of D/L-Hcy thiolactone (chemical shift 3.73 ppm) was labeled in the red rectangle. a) with *Ao*ACLR and PLP; b) with only *Ao*ACLR; c) with only PLP; d) control: without *Ao*ACLR and PLP. Reaction conditions: 0.5 mL reaction containing 2 mM D/L-Hcy thiolactone, 2 μM *Ao*ACLR, 20 μM PLP, and 50 mM sodium phosphate buffer in D_2O (pD 8.4) was incubated at room temperature. After 4 hours, the reaction was directly analyzed by ^{1}H NMR.

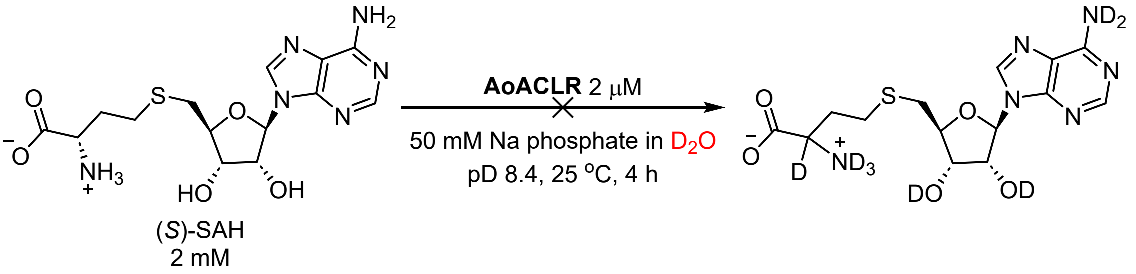

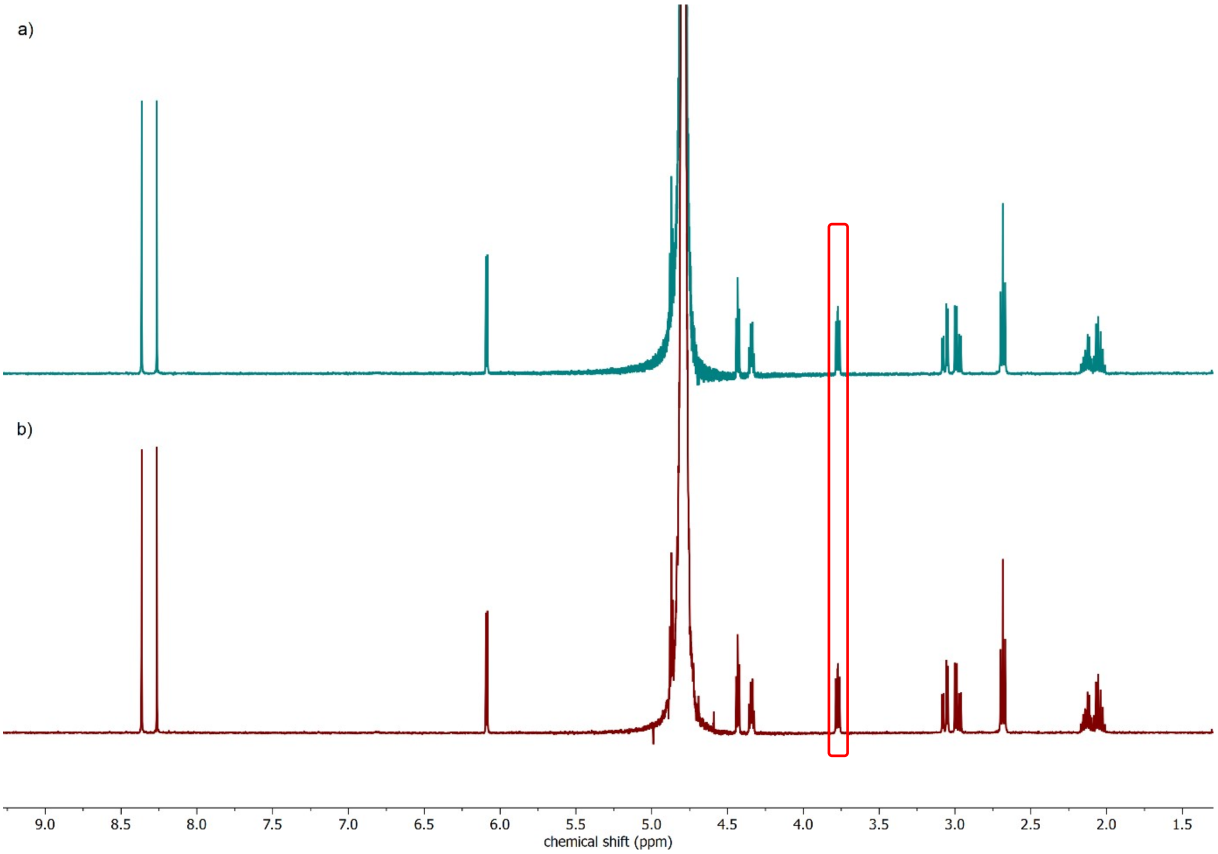

Figure S12. ^{1}H NMR spectra of racemization of (*S*)-SAH with *Ao*ACLR in D_2O. The α-H of (*S*)-SAH (chemical shift 3.77 ppm) was labeled in the red rectangle. a) with *Ao*ACLR; b) control: without *Ao*ACLR. Reaction conditions: 0.5 mL reaction containing 2 mM (*S*)-SAH, 2 μM *Ao*ACLR, and 50 mM sodium phosphate buffer in D_2O (pD 8.4) was incubated at room temperature. After 4 hours, the reaction was directly analyzed by ^{1}H NMR.

Production of SAH by ACLR-BLH-SAHH enzyme cascade

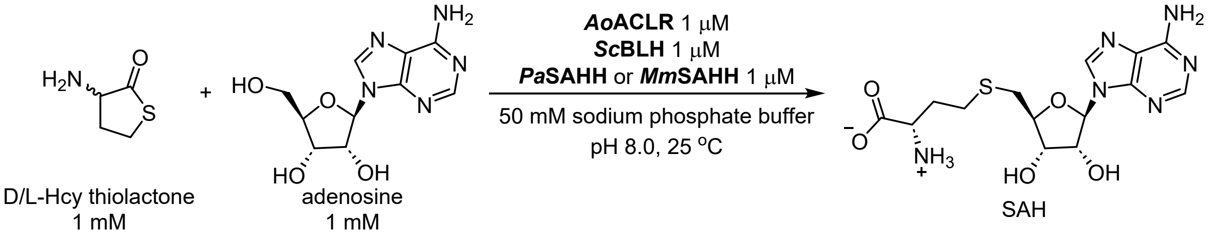

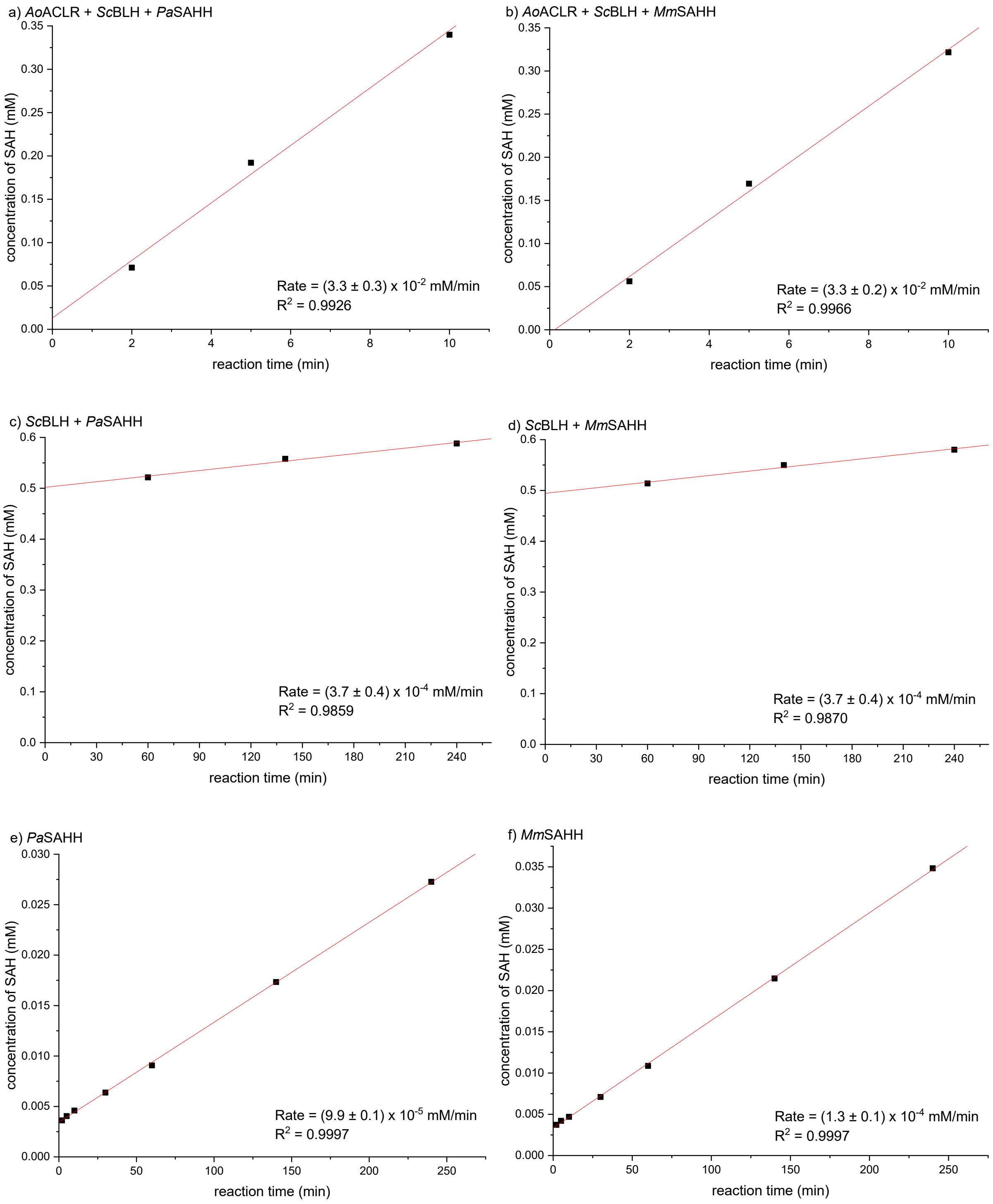

Figure S13. The kinetic parameters of SAH production by ACLR-BLH-SAHH enzyme cascade. The concentration of SAH was determined by HPLC. a) The initial rate of SAH production in the presence of *Ao*ACLR, *Sc*BLH and *Pa*SAHH; b) The initial rate of SAH production in the presence of *Ao*ACLR, *Sc*BLH and *Mm*SAHH; c) The rate of SAH production from Hcy generated by the uncatalyzed hydrolysis of Hcy thiolactone in the presence of *Sc*BLH and *Pa*SAHH; d) The rate of SAH production from Hcy generated by the uncatalyzed hydrolysis of Hcy thiolactone in the presence of *Sc*BLH and *Mm*SAHH; e) The initial rate of SAH production from Hcy generated by the uncatalyzed hydrolysis of Hcy thiolactone in the presence of *Pa*SAHH; f) The initial rate of SAH production from Hcy generated by the uncatalyzed hydrolysis of Hcy thiolactone in the presence of *Mm*SAHH. Reaction conditions: 200 μL reaction containing 1 mM D/L-Hcy thiolactone, 1 mM adenosine, 1 μM *Ao*ACLR, 1 μM *Sc*BLH, 1 μM *Pa*SAHH or *Mm*SAHH in 50 mM sodium phosphate buffer (pH 8.0) at 25 °C.

Large-scale enzymatic SAH synthesis

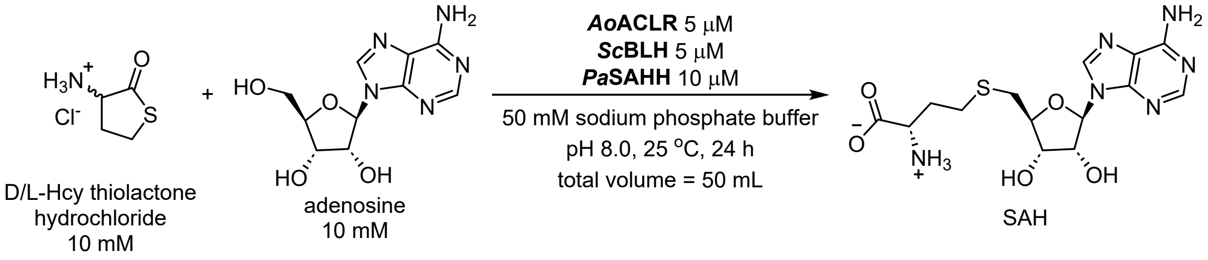

A 50 mL reaction containing 10 mM D/L-Hcy thiolactone hydrochloride (76.8 mg, 0.50 mmol), 10 mM adenosine (134 mg, 0.50 mmol), 5 μM *Ao*ACLR, 5 μM *Sc*BLH, 10 μM *Pa*SAHH and 50 mM sodium phosphate buffer (pH 8.0) were incubated at 25 °C. After 24 hours, the reaction was quenched by vigorously mixing with 50 mL of MeCN to precipitate enzymes. The denatured enzymes were removed by filtration and the filtrate was concentrated *in vacuo* to about 10 mL. The concentrated solution was purified by preparative reverse-phase HPLC (Column: Reprospher 100 C18-DE, particle size: 5 μm, length: 150 mm, inner diameter: 40 mm. Flow rate: 20 mL/min. Conditions: Buffer A: Milli-Q water with 0.1% (v/v) TFA; Buffer B: MeCN with 0.1% (v/v) TFA. 0-5 mins: 2% Buffer B; 5-25 mins: gradient of 2-40% Buffer B; 25-27 mins: gradient of 40-100% Buffer B; 27-32 mins: 100% Buffer B; 32-34 mins: gradient of 100-2% Buffer B). The eluted fractions were collected and lyophilized to give SAH·1.5 TFA salt as a white powder (217 mg, 78% yield).

^{1}H NMR (500 MHz, D_2O) δ 8.53 (s, 1H, H-9), 8.46 (s, 1H, H-12), 6.16 (d, J = 4.9 Hz, 1H, H-8), 4.88 (t, J = 5.1 Hz, 1H, H-7), 4.46 (t, J = 5.1 Hz, 1H, H-6), 4.36 (dt, J = 7.0, 4.9 Hz, 1H, H-5), 4.05 (dd, J = 6.8, 5.9 Hz, 1H, H-1), 3.09 (dd, J = 14.3, 4.9 Hz, 1H, H-4a), 3.03 (dd, J = 14.2, 6.9 Hz, 1H, H-4b), 2.74 (t, J = 7.5 Hz, 2H, H-3), 2.28–2.19 (m, 1H, H-2a), 2.18–2.09 (m, 1H, H-2b).

^{13}C NMR (126 MHz, D_2O) δ 172.5 ($\underline{C}O_2H$), 150.1 (C-11), 148.3 (C-13), 144.6 (C-12), 142.9 (C-9), 119.0 (C-10), 88.4 (C-8), 83.7 (C-5), 73.6 (C-7), 72.3 (C-6), 52.4 (C-1), 33.4 (C-4), 29.9 (C-2), 27.4 (C-3).

^{19}F NMR (470 MHz, D_2O) δ -75.59 (s).

HRMS (ESI) Cald. for $C_{14}H_{21}N_6O_5S$ (M+H$^+$) 385.1289, found 385.1290.

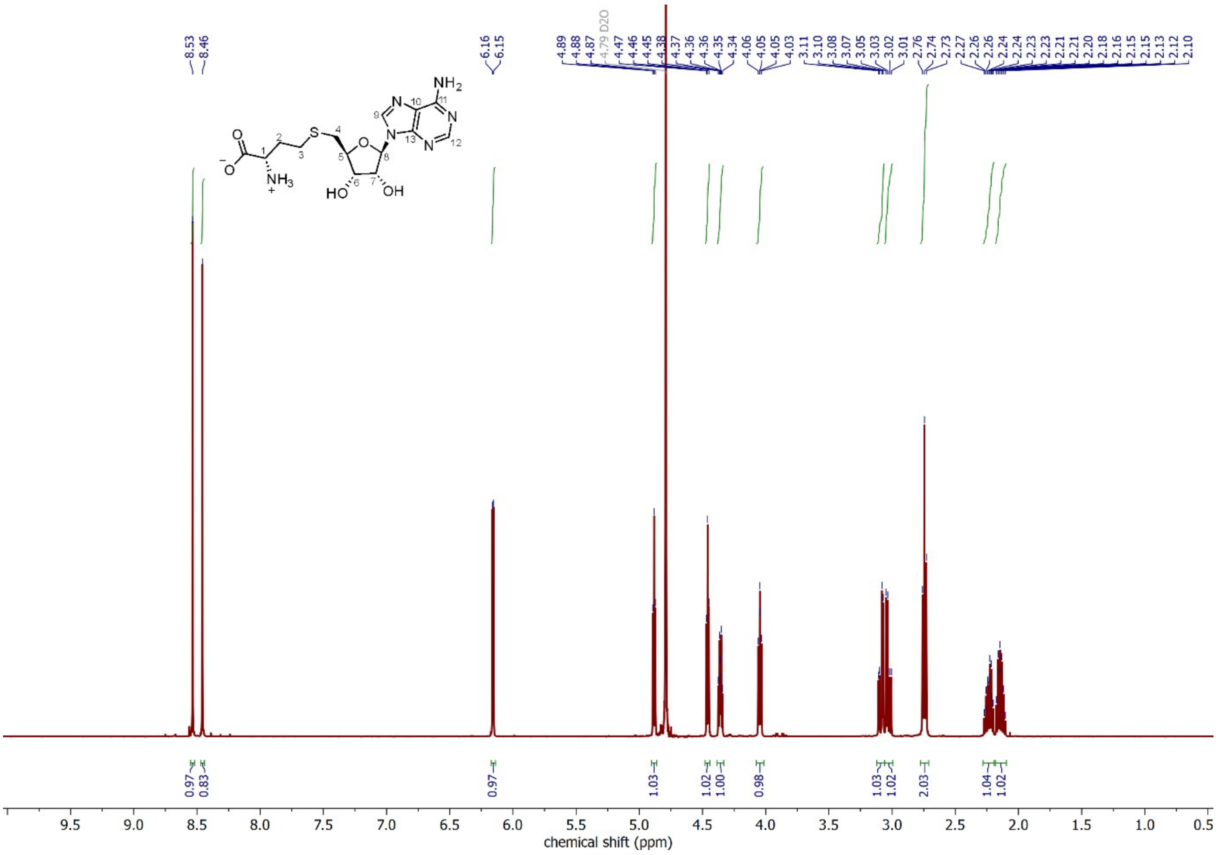

Figure S14. ^{1}H NMR spectrum of SAH·1.5 TFA salt.

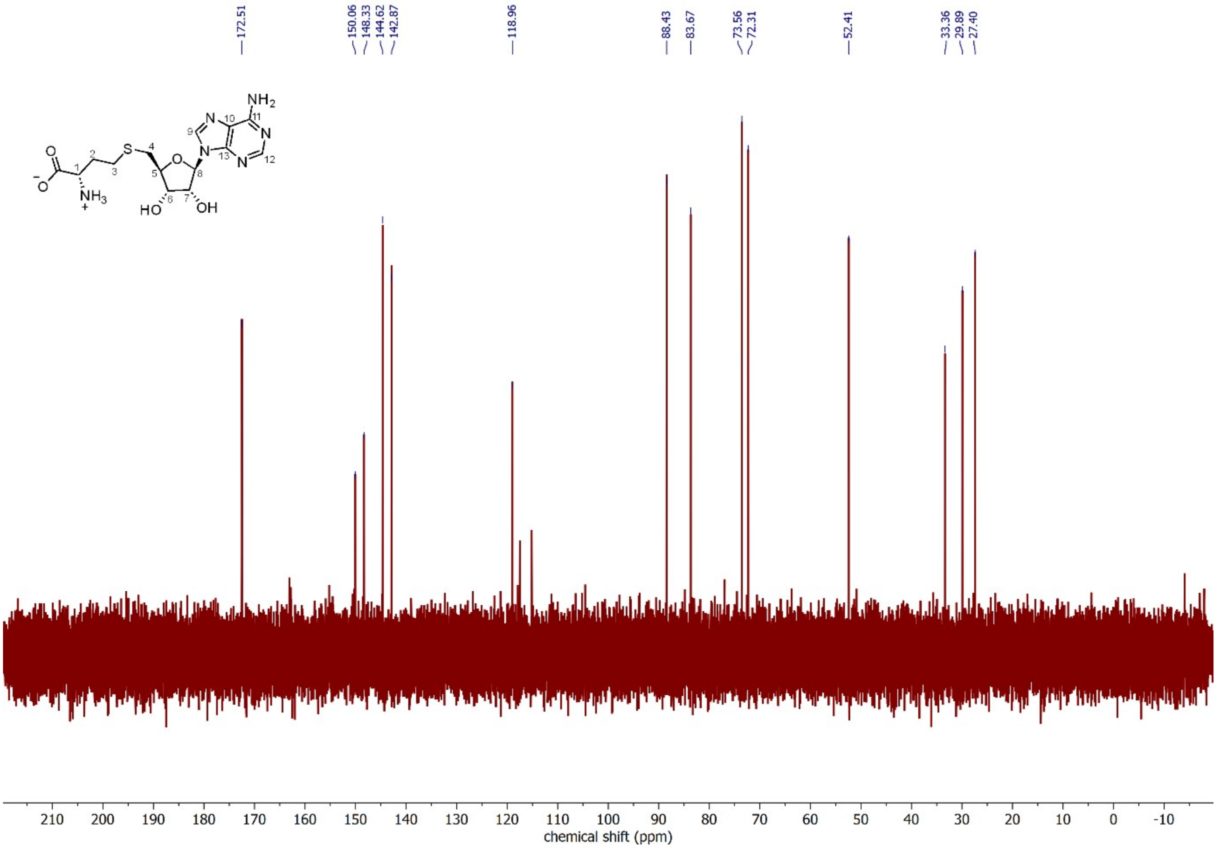

Figure S15. ^{13}C NMR spectrum of SAH·1.5 TFA salt.

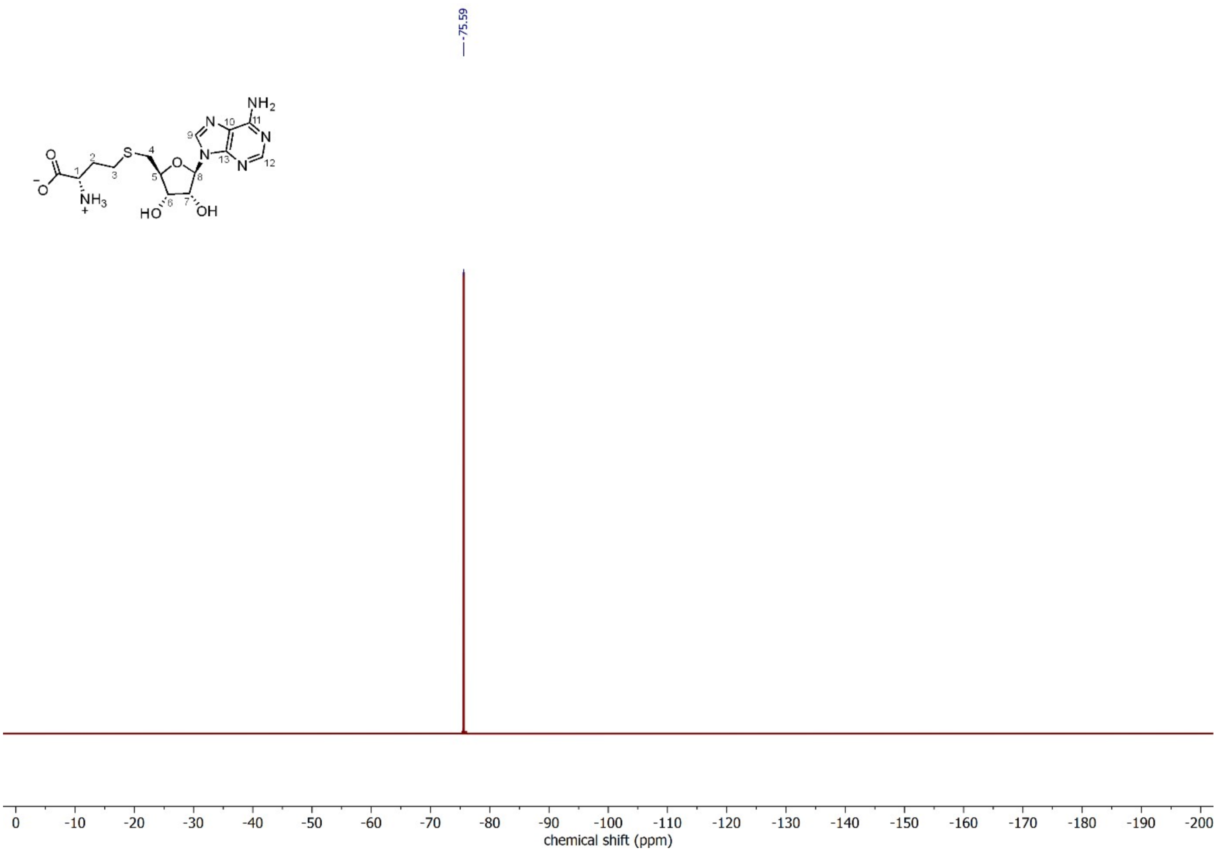

Figure S16. ^{19}F NMR spectrum of SAH·1.5 TFA salt.

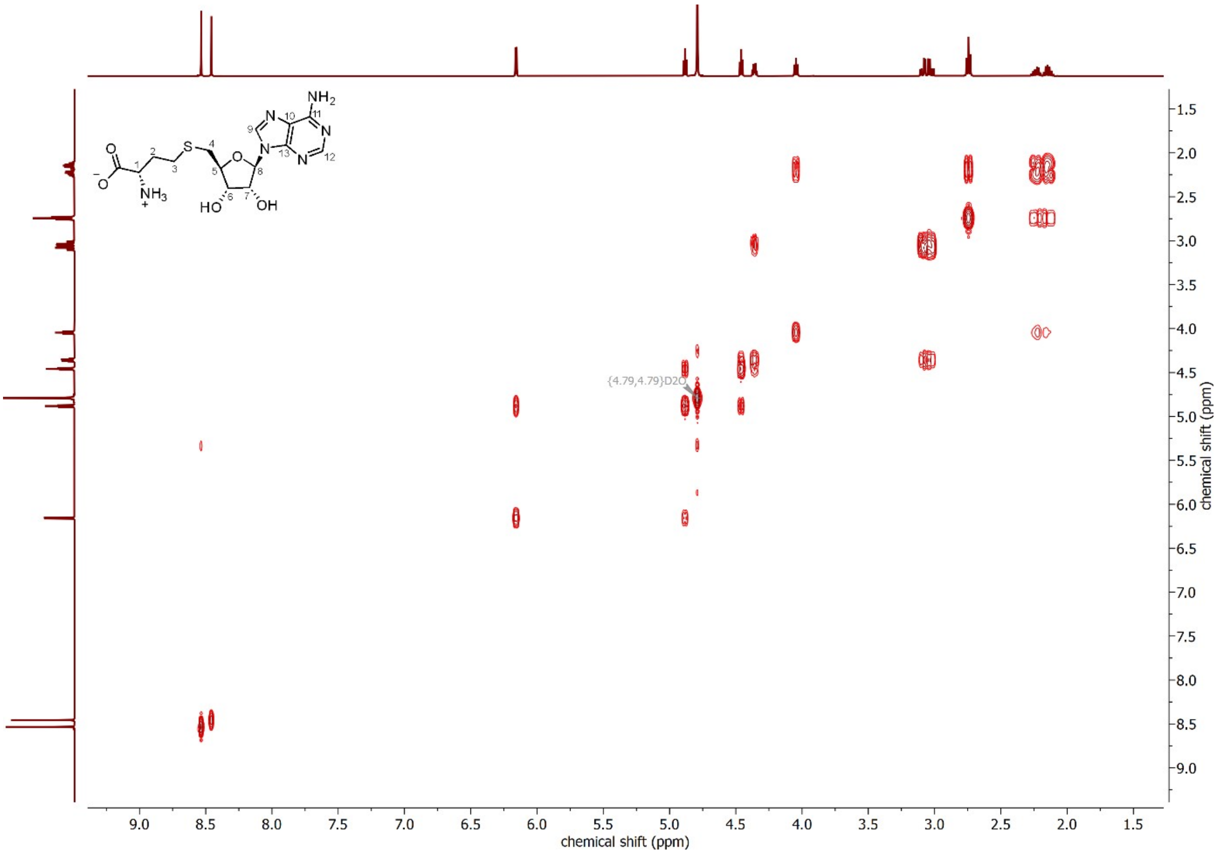

Figure S17. ^{1}H-^{1}H COSY spectrum of SAH·1.5 TFA salt.

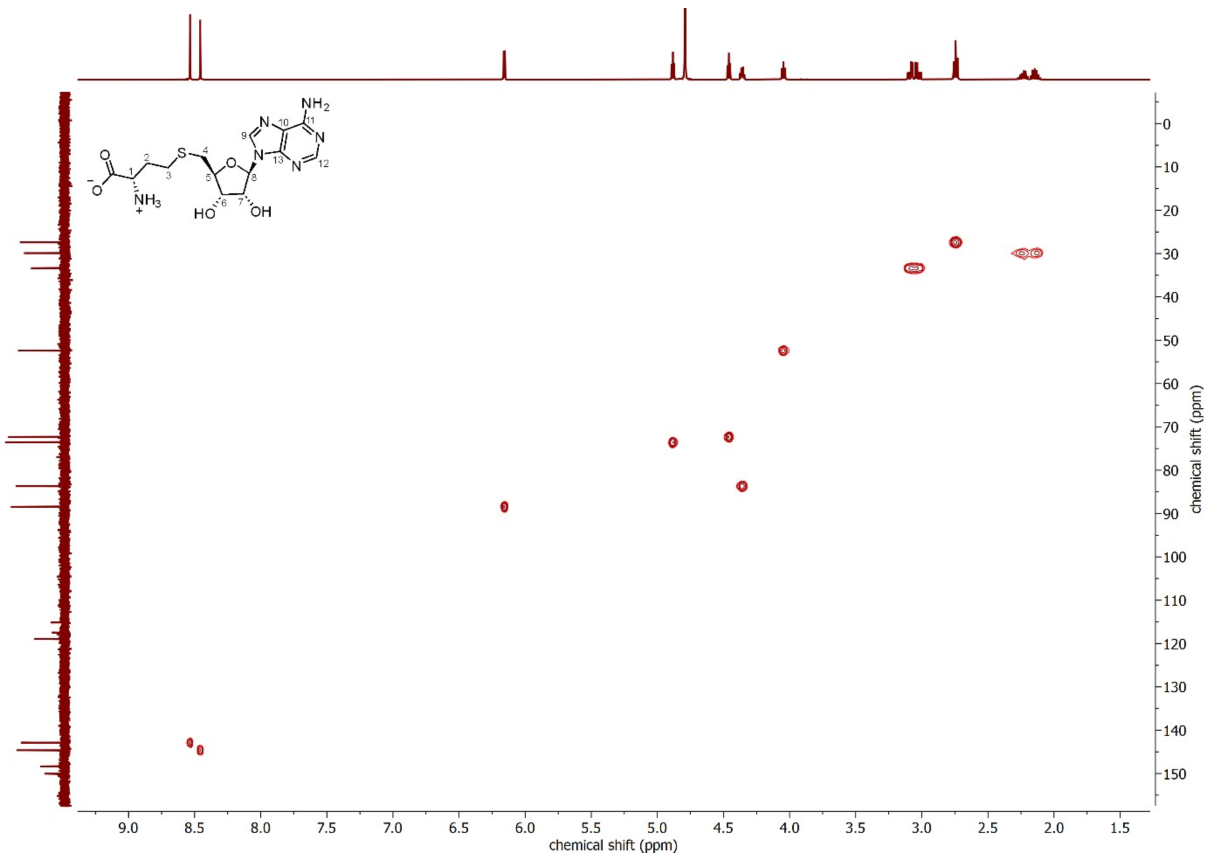

Figure S18. HSQC spectrum of SAH·1.5 TFA salt.

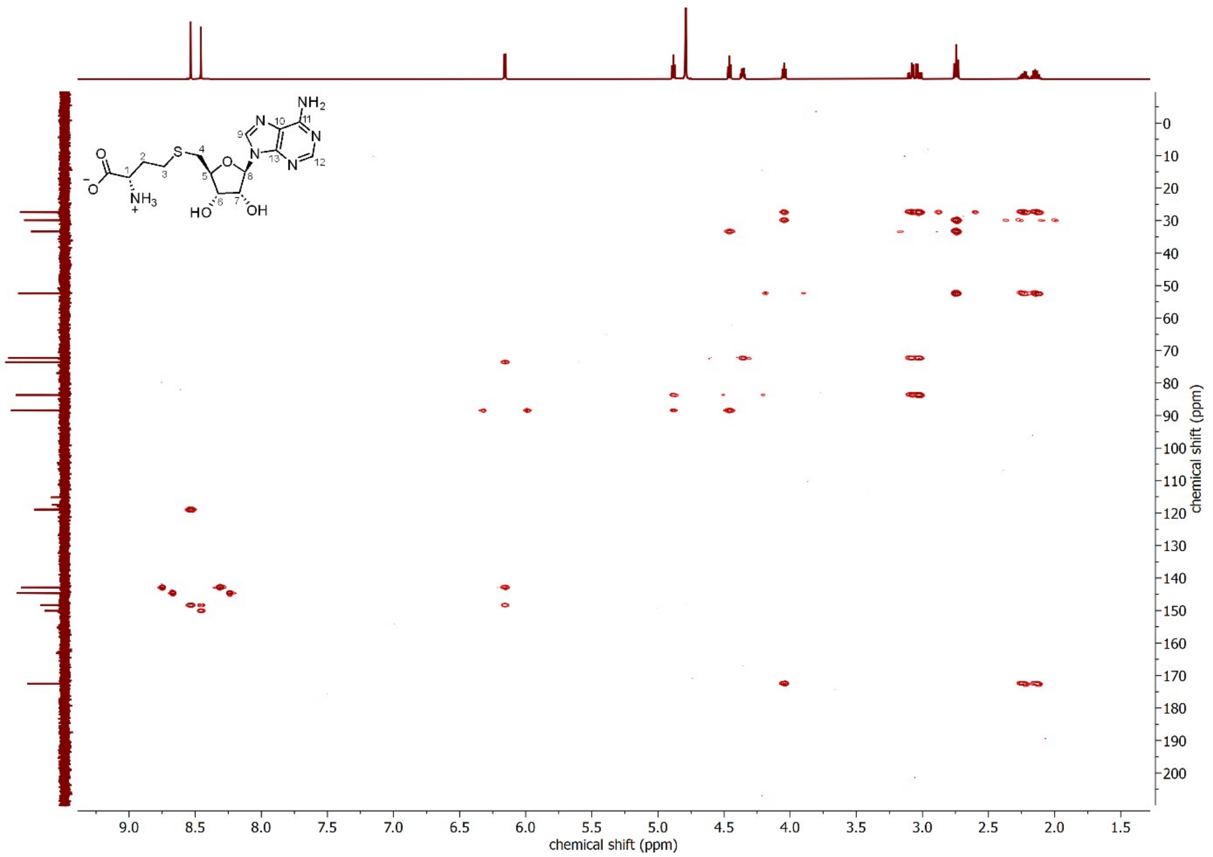

Figure S19. HMBC spectrum of SAH·1.5 TFA salt.

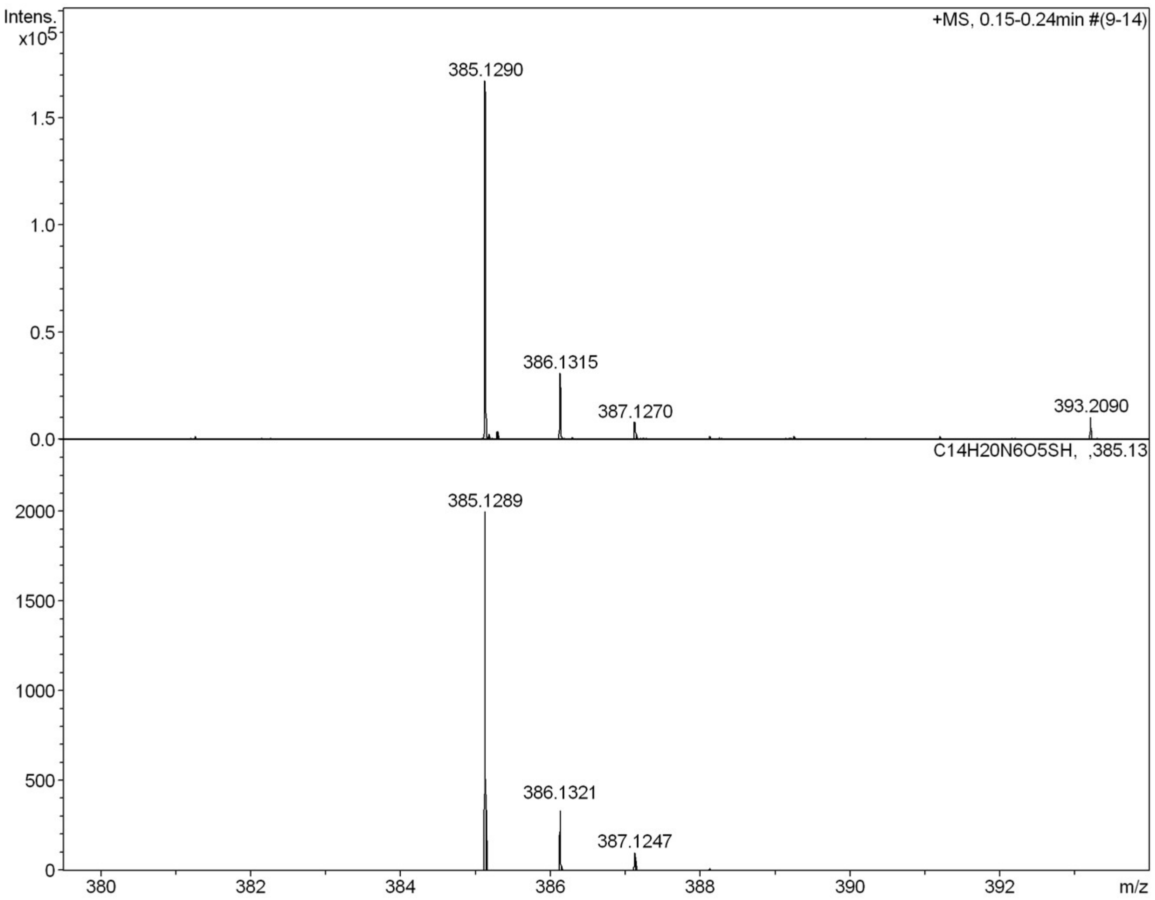

Figure S20. Measured (top) and calculated (bottom) HR-ESI-MS spectra of SAH·1.5 TFA salt.

Production of SAM by two-step enzyme cascade

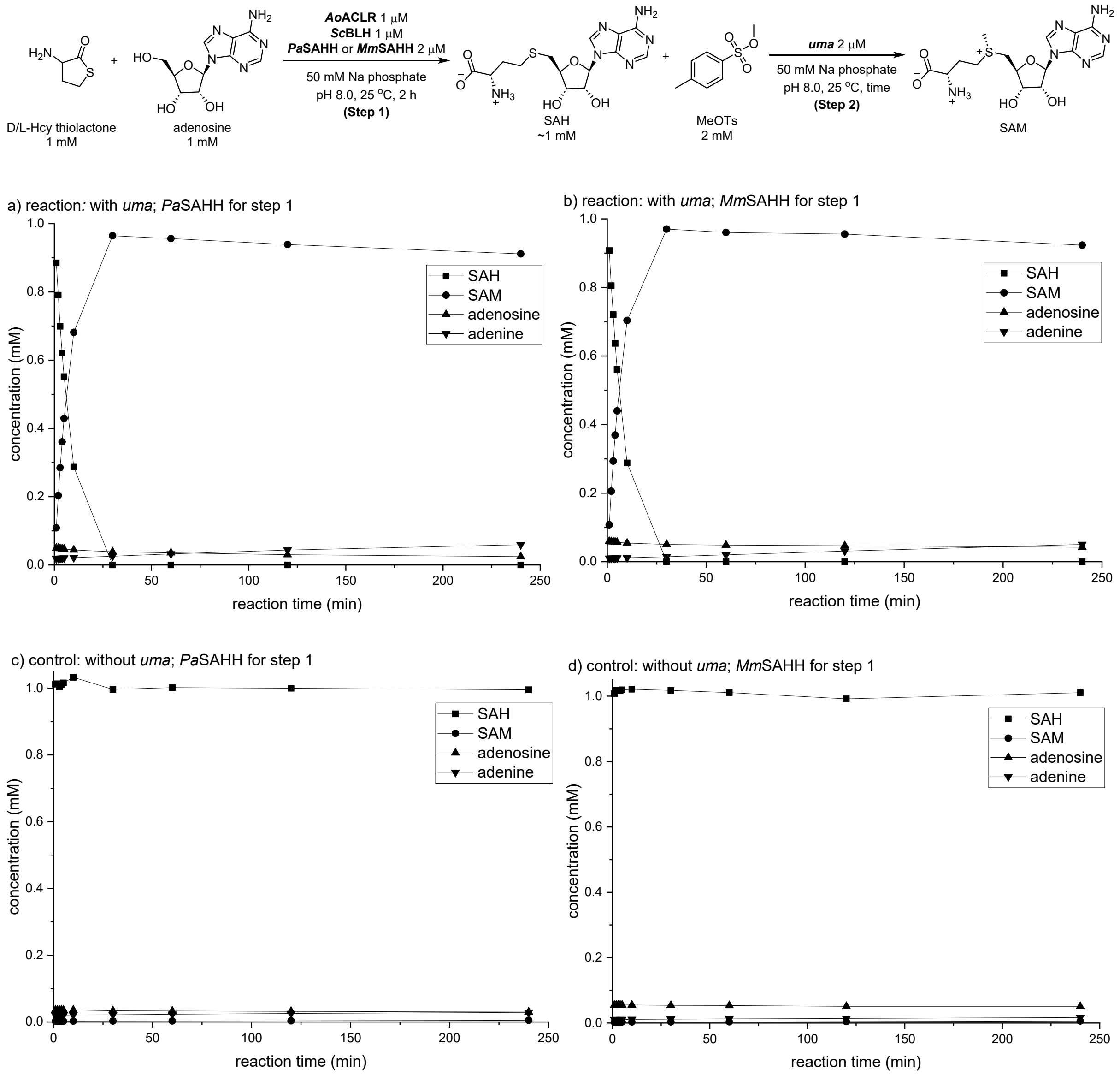

Figure S21. Time-dependent one-pot uma-catalyzed SAM formation from MeOTs and SAH generated by *Ao*ACLR-*Sc*BLH-SAHH enzyme cascade. The concentrations of all SAH derivatives were determined by HPLC. a) Reaction: with uma for the second step, using *Pa*SAHH for the first step; b) Reaction: with uma for the second step, using *Mm*SAHH for the first step; c) Control: without uma for the second step, using *Pa*SAHH for the first step; d) Control : without uma for the second step, using *Mm*SAHH for the first step. Reaction conditions: For the first step, 200 µL reaction containing 1 mM D/L-Hcy thiolactone, 1 mM adenosine, 1 µM *Ao*ACLR, 1 µM *Sc*BLH, 2 µM *Pa*SAHH or *Mm*SAHH in 50 mM sodium phosphate buffer (pH 8.0) was incubated at 25 °C for 2 h. Then 2 µM uma and 2 mM MeOTs were directly added to the reaction to start the second step and the reaction continued to be incubated at 25 °C.

Large-scale synthesis of SAM by two-step enzyme cascade

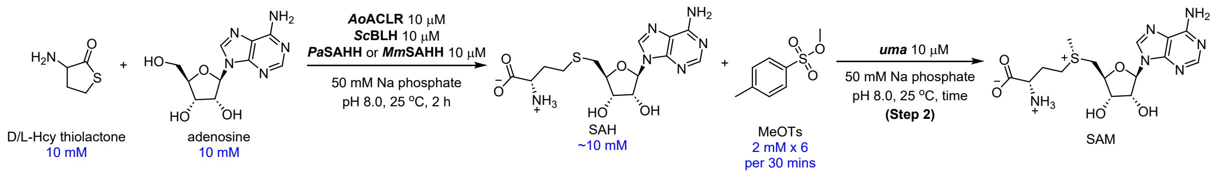

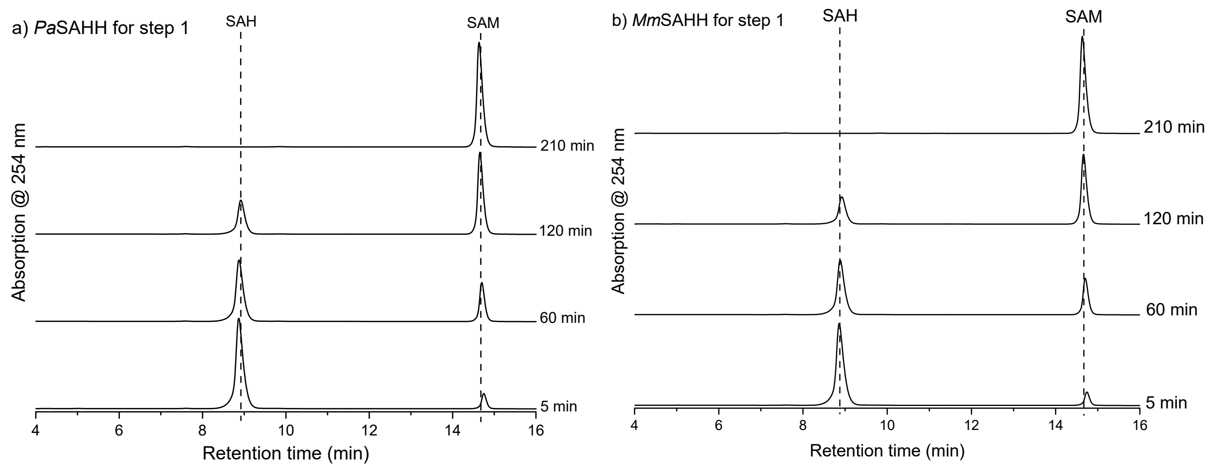

Figure S22. HPLC analysis monitoring one-pot uma-catalyzed SAM formation from MeOTs and SAH generated by *Ao*ACLR-*Sc*BLH-SAHH enzyme cascade. a) Using *Pa*SAHH for the first step; b) Using *Mm*SAHH for the first step. Reaction conditions: For the first step, 500 µL reaction containing 10 mM D/L-Hcy thiolactone, 10 mM adenosine, 10 µM *Ao*ACLR, 10 µM *Sc*BLH, 10 µM *Pa*SAHH or *Mm*SAHH in 50 mM sodium phosphate buffer (pH 8.0) was incubated at 25 °C for 2 h. The second step was initiated by adding 10 µM uma and 2 mM MeOTs directly to the reaction. Additional 2 mM doses of MeOTs were added after 30, 60, 90, 120 and 150 minutes.

Enzymatic methylation using SAH produced by three-enzyme cascade as a catalyst

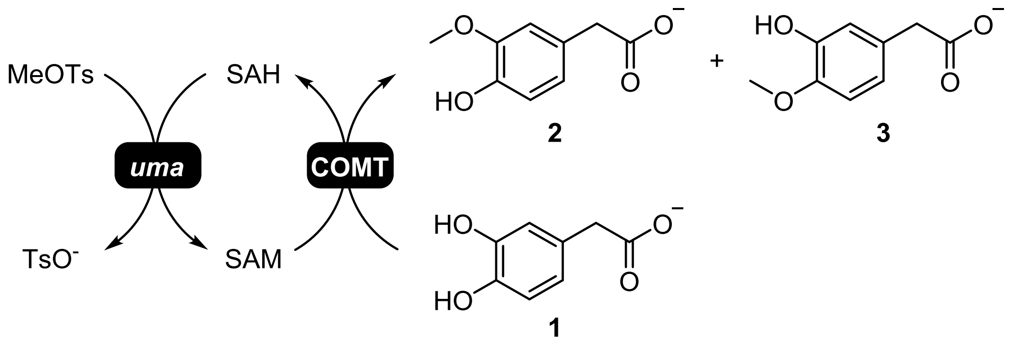

1 mL reactions containing 2 mM 3,4-dihydroxyphenylacetic acid **1**, 4 mM MeOTs, 4 mM $MgCl_2$, 40 µM SAH (diluted from 1 mM SAH stock solution produced by *Ao*ACLR-*Sc*BLH-*Pa*SAHH enzyme cascade without any purification), 10 µM uma, 20 µM human catechol *O*-methyltransferase (COMT) and 50 mM sodium phosphate buffer (pH 8.0) were incubated at 25 °C. After 24 hours, the reaction was quenched by the addition of 500 µL of $CHCl_3$ and vigorous mixing on the vortex. The aqueous layer was isolated by centrifugation and lyophilized. After lyophilization, the mixture was dissolved in 500 µL of D_2O. ^{1}H NMR was recorded. The ratio of the substrate, 3-*O*, and 4-*O* methylation products **2** and **3** was determined by the integration of characteristic ^{1}H NMR resonances from these compounds.

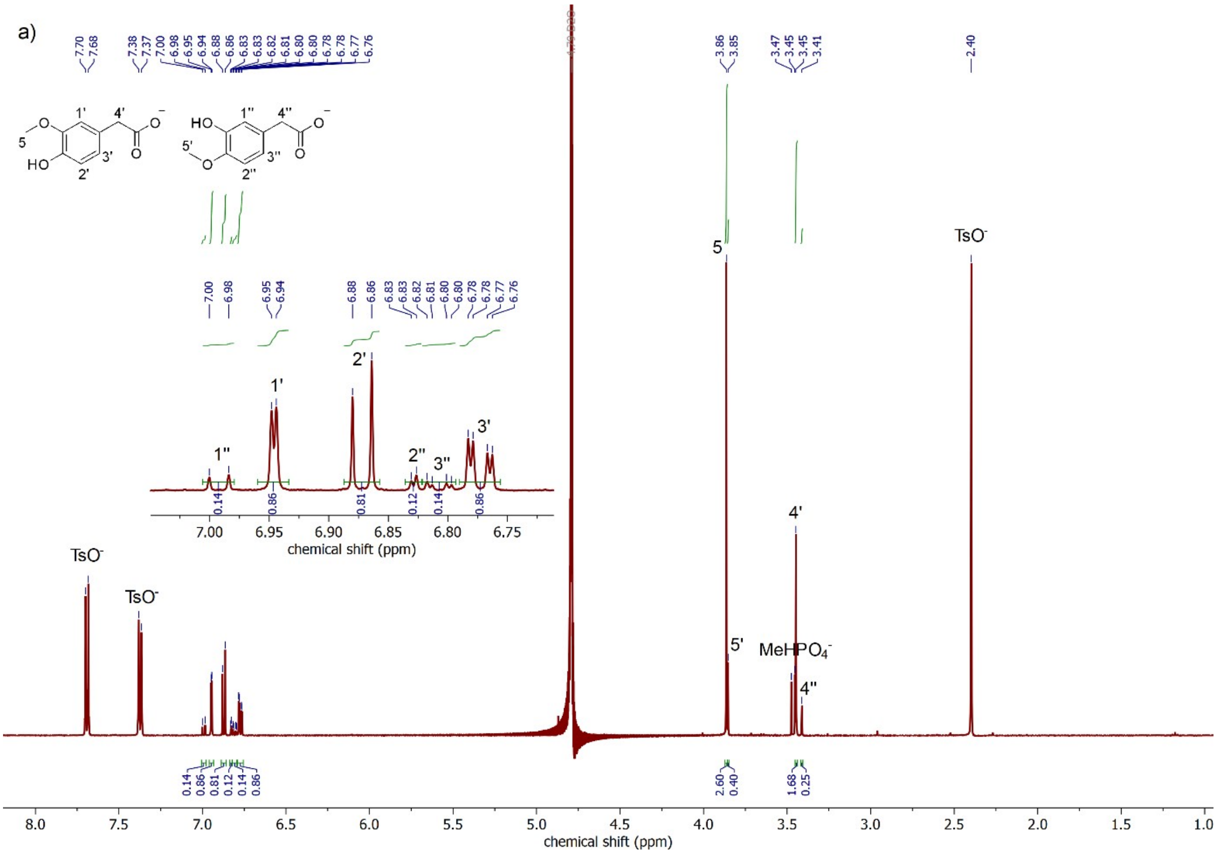

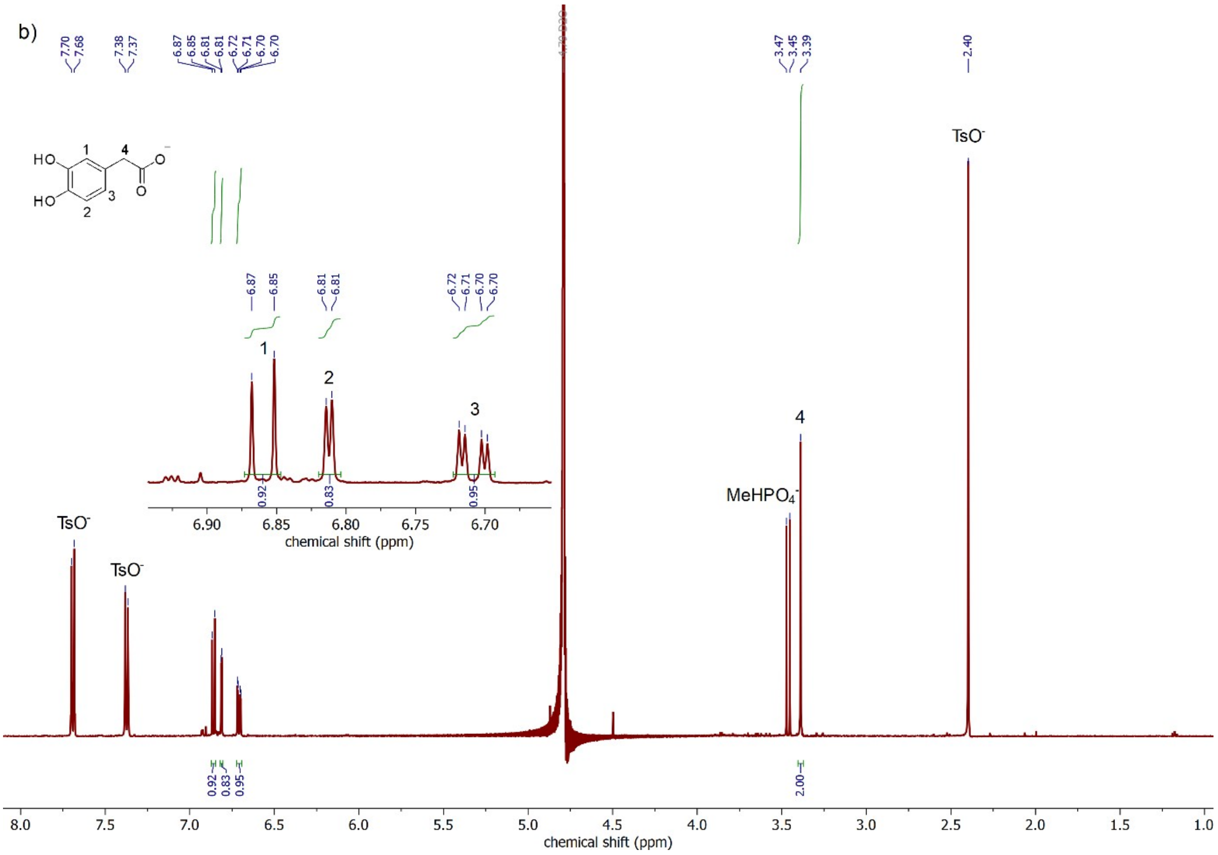

Figure S23. ¹H NMR spectra of uma-COMT cascade reaction using 3,4-dihydroxyphenylacetic acid **1** as a substrate. a) Reaction with uma and COMT enzymes. The 3-*O* and 4-*O* methylation products **2** and **3** were identified by comparison to the ¹H NMR spectra of commercially available compounds. No signal of substrate **1** was observed in the reaction mixture. Therefore, the total yield of products **2** and **3** was estimated to be 100%. The yield of each product was calculated based on integrals of methoxyl groups from products. The yield of **2** = 2.60 / (2.60 + 0.40) x 100% = 87%; the yield of **3** = 0.40 / (2.60+ 0.40) x 100% = 13%. b) Reaction without enzymes. Only the signal of the substrate **1** was observed.

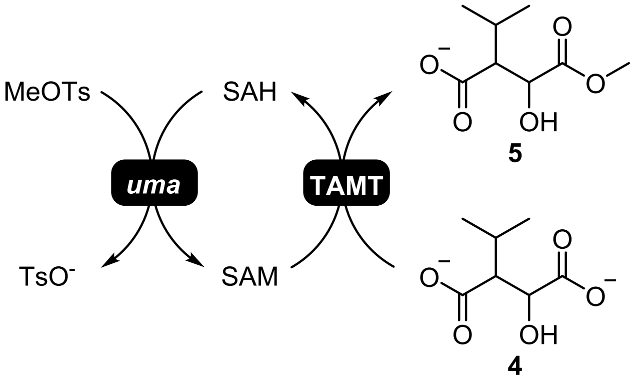

1 mL reaction containing 2 mM 3-isopropylmalate **4**, 4 mM MeOTs, 40 µM SAH (diluted from 1 mM SAH stock solution produced by *Ao*ACLR-*Sc*BLH-*Pa*SAHH enzyme cascade without any purification), 5 µM uma, 5 µM *trans*-aconitate 3-methyltransferase (TAMT), and 50 mM sodium phosphate buffer (pH 8.0) were incubated at 25 °C. After 24 hours, the reaction was quenched by the addition of 500 µL of $CHCl_3$ and vigorous mixing on the vortex. The aqueous layer was isolated by centrifugation and lyophilized. After lyophilization, the mixture was dissolved in 500 µL of D_2O. ^{1}H NMR was recorded. The ratio of substrate and product 3-isopropylmalate methyl ester **5** was determined by the integration of characteristic ^{1}H NMR resonances from these compounds.

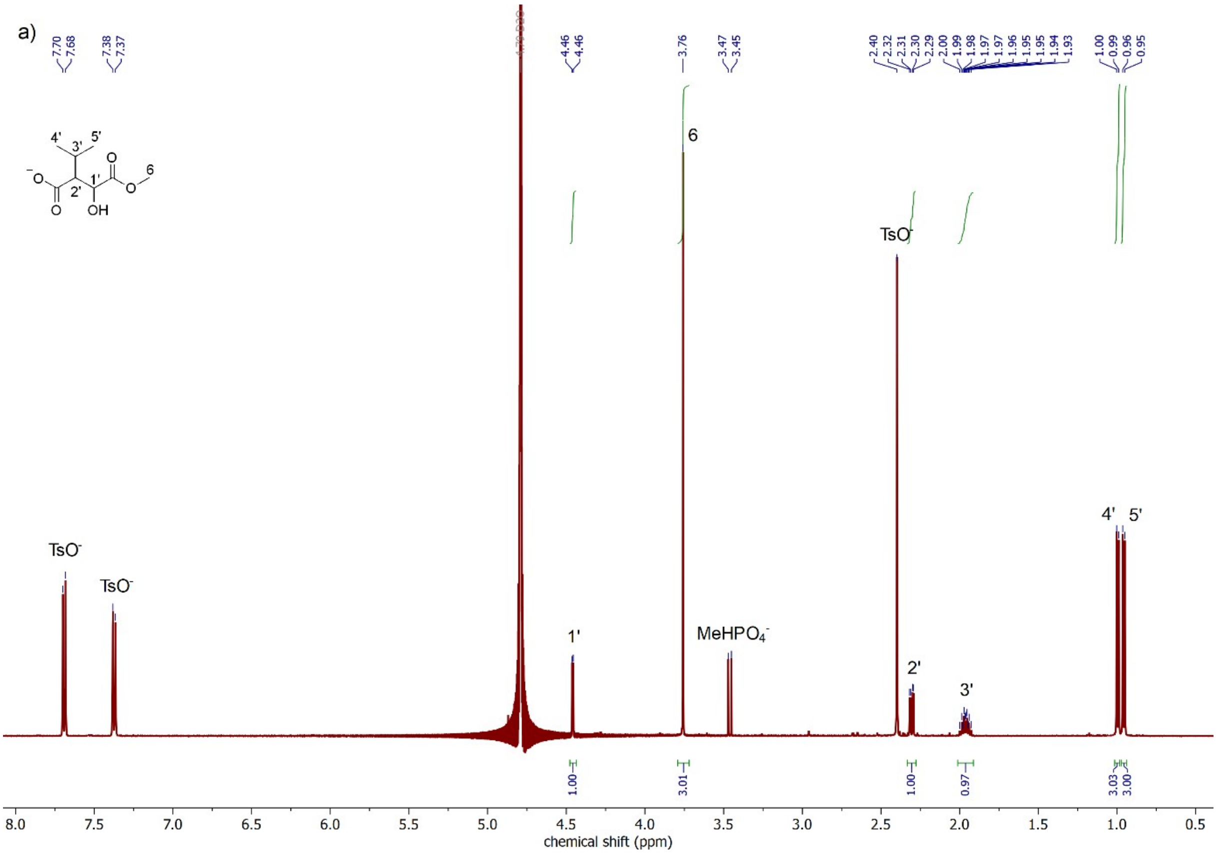

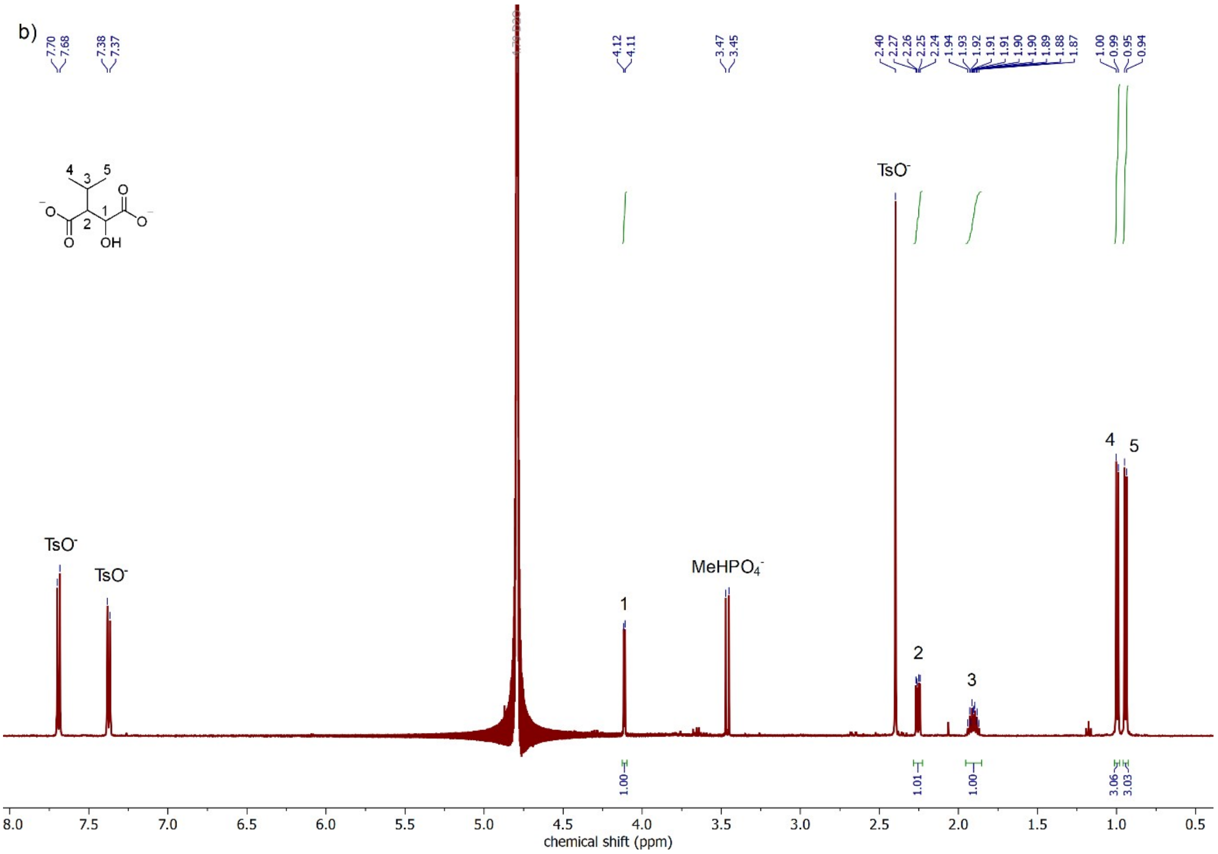

Figure S24. ^{1}H NMR spectra of uma-TAMT cascade reaction using 3-isopropylmalate **4** as a substrate. a) Reaction with uma and TAMT enzymes. No signal of substrate was observed in the reaction mixture. Therefore, the yield of product 3-isopropylmalate methyl ester **5** was estimated to be over 99%. ^{1}H NMR of **5** (500 MHz, D_2O, pD 8.4) δ 4.46 (d, J = 3.5 Hz, 1H, H-1'), 3.76 (s, 3H, H-6), 2.31 (dd, J = 9.7, 3.5 Hz, 1H, H-2'), 2.02–1.91 (m, 1H, H-3'), 1.00 (d, J = 6.7 Hz, 3H, H-4'), 0.96 (d, J = 6.7 Hz, 3H, H-5'). b) Reaction without enzymes. Only the signal of the substrate **4** was observed.

Production of SAH derivatives by *Ao*ACLR-*Sc*BLH-SAHH enzyme cascades

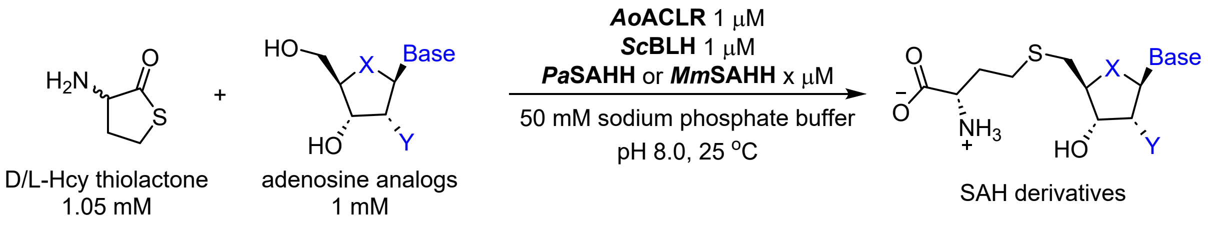

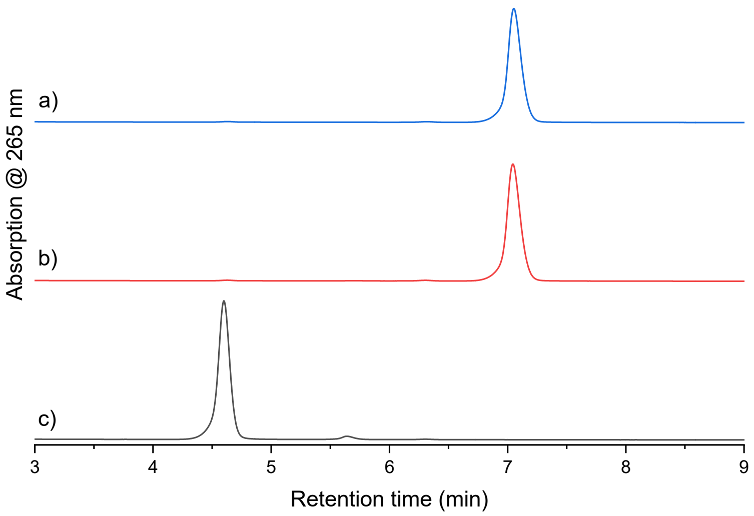

Entry	Retention time of adenosine (min)	Retention time of SAH (min)	Peak area of adenosine	Peak area of SAH	Yield of SAH
a	4.63	7.05	133	31796	>99%
b	4.63	7.05	126	31140	>99%

Figure S25. HPLC analysis monitoring SAH synthesis by *Ao*ACLR-*Sc*BLH-SAHH enzyme cascade from D/L-Hcy thiolactone and adenosine. a) with *Mm*SAHH; b) with *Pa*SAHH; c) control: without enzymes. The yield of SAH = peak area of SAH / (peak area of adenosine + peak area of SAH) x 100%. Reaction conditions: 200 μL reaction containing 1.05 mM D/L-Hcy thiolactone, 1 mM adenosine, 1 μM *Ao*ACLR, 1 μM *Sc*BLH, 1 μM *Pa*SAHH or *Mm*SAHH and 50 mM sodium phosphate buffer (pH 8.0) was incubated at 25 °C for 2 h.

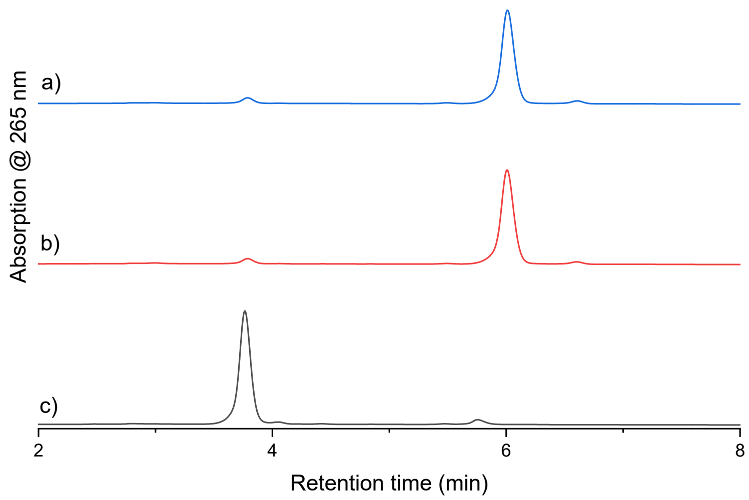

Entry	Retention time of nebularine (min)	Retention time of SNH (min)	Peak area of nebularine	Peak area of SNH	Yield of SNH
a	3.79	6.01	836	16179	95%
b	3.78	6.01	701	16293	96%

Figure S26. HPLC analysis monitoring synthesis of SNH by *Ao*ACLR-*Sc*BLH-SAHH enzyme cascade from D/L-Hcy thiolactone and nebularine. a) with *Mm*SAHH; b) with *Pa*SAHH; c) control: without enzymes. The yield of SNH = peak area of SNH / (peak area of nebularine + peak area of SNH) x 100%. Reaction conditions: 200 µL reaction containing 1.05 mM D/L-Hcy thiolactone, 1 mM nebularine, 1 µM *Ao*ACLR, 1 µM *Sc*BLH, 5 µM *Pa*SAHH or *Mm*SAHH and 50 mM sodium phosphate buffer (pH 8.0) was incubated at 25 °C for 4 h.

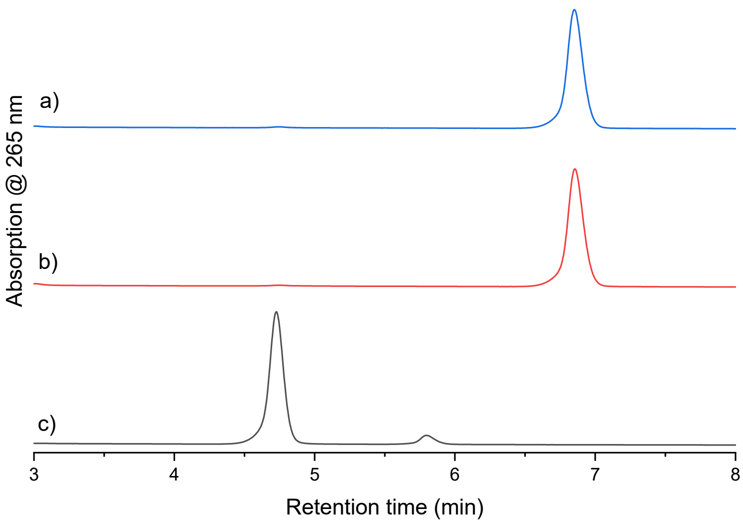

Entry	Retention time of formycin A (min)	Retention time of SFH (min)	Peak area of formycin A	Peak area of SFH	Yield of SFH
a	4.75	6.85	134	10656	99%
b	4.74	6.86	94	10737	99%

Figure S27. HPLC analysis monitoring synthesis of SFH by *Ao*ACLR-*Sc*BLH-SAHH enzyme cascade from D/L-Hcy thiolactone and formycin A. a) with *Mm*SAHH; b) with *Pa*SAHH; c) control: without enzymes. The yield of SFH = peak area of SFH / (peak area of formycin A + peak area of SFH) x 100%. Reaction conditions: 200 µL reaction containing 1.05 mM D/L-Hcy thiolactone, 1 mM formycin A, 1 µM *Ao*ACLR, 1 µM *Sc*BLH, 5 µM *Pa*SAHH or *Mm*SAHH and 50 mM sodium phosphate buffer (pH 8.0) was incubated at 25 °C for 4 h.

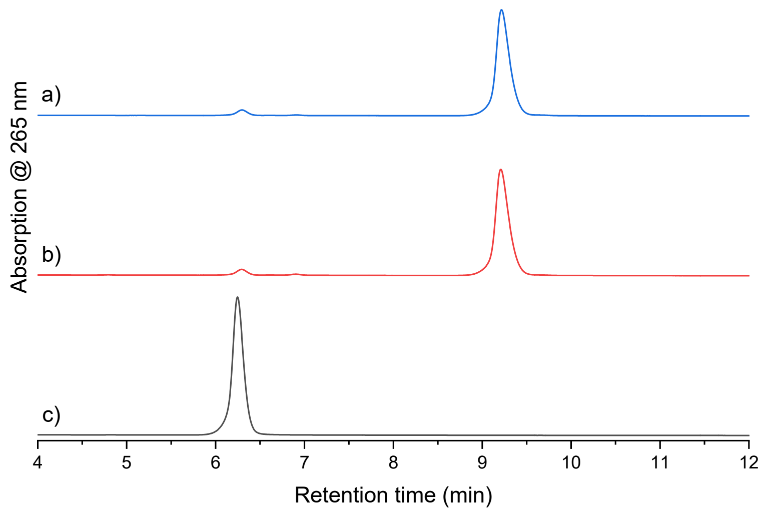

Entry	Retention time of aristeromycin (min)	Retention time of SArH (min)	Peak area of aristeromycin	Peak area of SArH	Yield of SArH
a	6.29	9.22	1577	38554	96%
b	6.30	9.21	1575	38564	96%

Figure S28. HPLC analysis monitoring synthesis of SArH by *Ao*ACLR-*Sc*BLH-SAHH enzyme cascade from D/L-Hcy thiolactone and aristeromycin. a) with *Mm*SAHH; b) with *Pa*SAHH; c) control: without enzymes. The yield of SArH = peak area of SArH / (peak area of aristeromycin + peak area of SArH) x 100%. Reaction conditions: 200 µL reaction containing 1.05 mM D/L-Hcy thiolactone, 1 mM aristeromycin, 1 µM *Ao*ACLR, 1 µM *Sc*BLH, 20 µM *Pa*SAHH or *Mm*SAHH and 50 mM sodium phosphate buffer (pH 8.0) was incubated at 25 °C for 20 h.

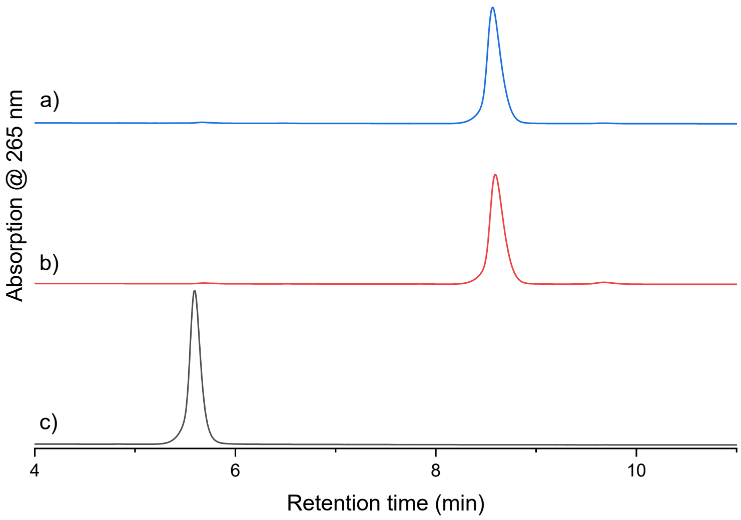

Entry	Retention time of 3-deazaadenosine (min)	Retention time of $S^{3\text{-deaza}}AH$ (min)	Peak area of 3-deazaadenosine	Peak area of $S^{3\text{-deaza}}AH$	Yield of $S^{3\text{-deaza}}AH$
a	5.69	8.56	181	24430	99%
b	5.67	8.59	231	25814	99%

Figure S29. HPLC analysis monitoring synthesis of $S^{3\text{-deaza}}AH$ by *Ao*ACLR-*Sc*BLH-SAHH enzyme cascade from D/L-Hcy thiolactone and 3-deazaadenosine. a) with *Mm*SAHH; b) with *Pa*SAHH; c) control: without enzymes. The yield of $S^{3\text{-deaza}}AH$ = peak area of $S^{3\text{-deaza}}AH$ / (peak area of 3-deazaadenosine + peak area of $S^{3\text{-deaza}}AH$) x 100%. Reaction conditions: 200 µL reaction containing 1.05 mM D/L-Hcy thiolactone, 1 mM 3-deazaadenosine, 1 µM *Ao*ACLR, 1 µM *Sc*BLH, 5 µM *Pa*SAHH or *Mm*SAHH, and 50 mM sodium phosphate buffer (pH 8.0) was incubated at 25 °C for 1 h.

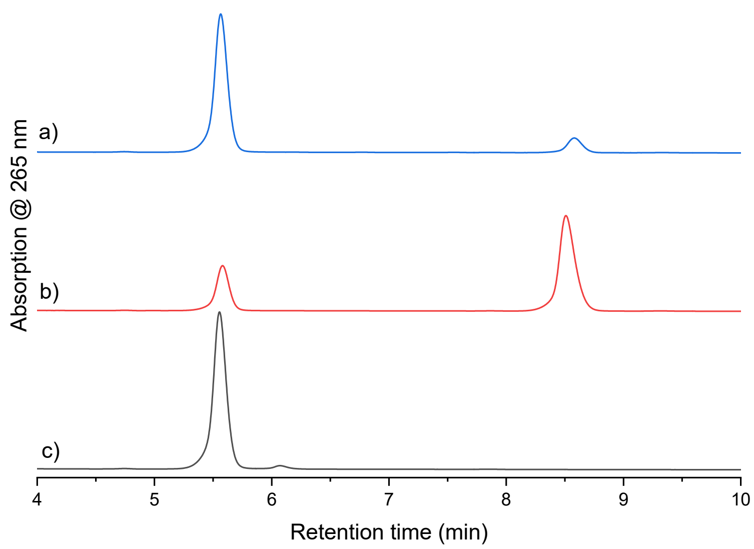

Entry	Retention time of tubercidin (min)	Retention time of STH (min)	Peak area of tubercidin	Peak area of STH	Yield of STH
a	5.57	8.58	24471	2970	11%
b	5.58	8.51	7897	20434	72%

Figure S30. HPLC analysis monitoring synthesis of STH by *Ao*ACLR-*Sc*BLH-SAHH enzyme cascade from D/L-Hcy thiolactone and tubercidin. a) with *Mm*SAHH; b) with *Pa*SAHH; c) control: without enzymes. The yield of STH = peak area of STH / (peak area of tubercidin + peak area of STH) x 100%. Reaction conditions: 200 μL reaction containing 1.05 mM D/L-Hcy thiolactone, 1 mM tubercidin, 1 μM *Ao*ACLR, 1 μM *Sc*BLH, 20 μM *Pa*SAHH or *Mm*SAHH and 50 mM sodium phosphate buffer (pH 8.0) was incubated at 25 °C for 44 h.

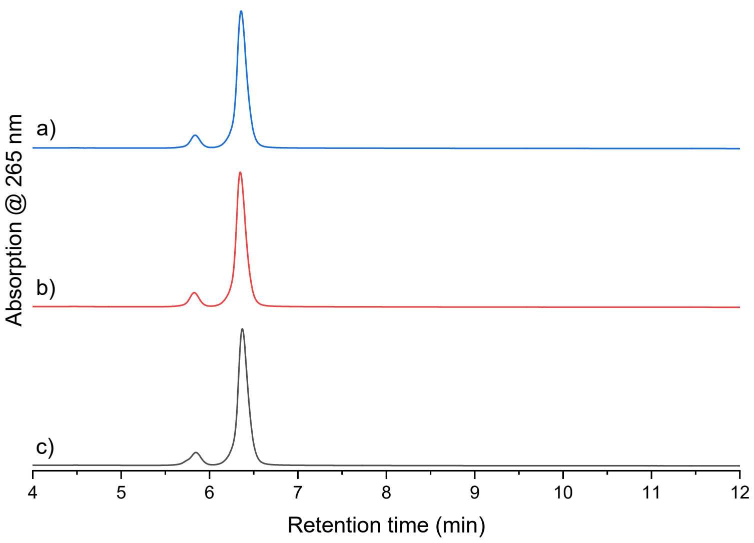

Entry	Retention time of 2'-deoxyadenosine (min)	Retention time of SdAH (min)	Peak area of 2'-deoxyadenosine	Peak area of SdAH	Yield of SdAH
a	6.36	N.D.	37584	N.D.	<1%
b	6.35	N.D.	36868	N.D.	<1%

Figure S31. HPLC analysis monitoring synthesis of SdAH by *Ao*ACLR-*Sc*BLH-SAHH enzyme cascade from D/L-Hcy thiolactone and 2'-deoxyadenosine. a) with *Mm*SAHH; b) with *Pa*SAHH; c) control: without enzymes. N.D. = not detected. As the target product SdAH could not be detected by HPLC in all enzymatic reactions, we assumed that the yield of SdAH was less than 1%. Reaction conditions: 200 µL reaction containing 1.05 mM D/L-Hcy thiolactone, 1 mM 2'-deoxyadenosine, 1 µM *Ao*ACLR, 1 µM *Sc*BLH, 5 µM *Pa*SAHH or *Mm*SAHH and 50 mM sodium phosphate buffer (pH 8.0) was incubated at 25 °C for 20 h.

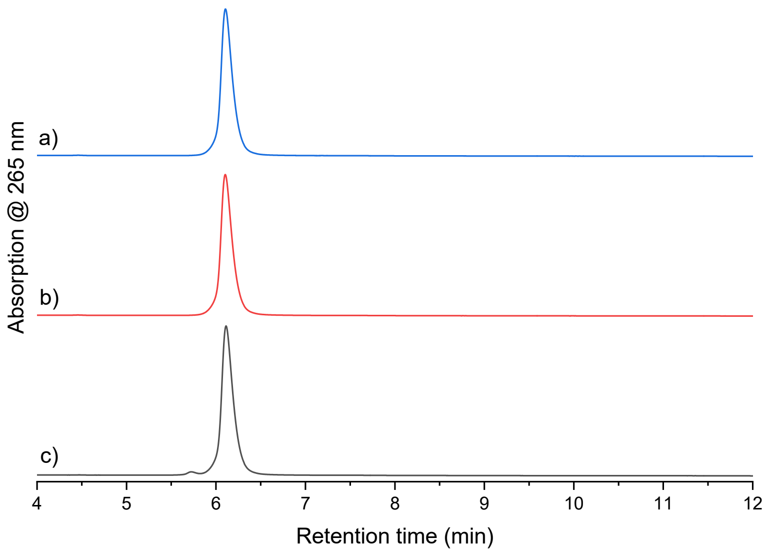

Entry	Retention time of 2'-*O*-methyladenosine (min)	Retention time of $S^{O2'\text{-}Me}AH$ (min)	Peak area of 2'-*O*-methyladenosine	Peak area of $S^{O2'\text{-}Me}AH$	Yield of $S^{O2'\text{-}Me}AH$
a	6.11	N.D.	38303	N.D.	<1%
b	6.11	N.D.	36663	N.D.	<1%

Figure S32. HPLC analysis monitoring synthesis of $S^{O2'\text{-}Me}AH$ by *Ao*ACLR-*Sc*BLH-SAHH enzyme cascade from D/L-Hcy thiolactone and 2'-*O*-methyladenosine. a) with *Mm*SAHH; b) with *Pa*SAHH; c) control: without enzymes. N.D. = not detected. As the target product $S^{O2'\text{-}Me}AH$ could not be detected by HPLC in all enzymatic reactions, we assumed that the yield of $S^{O2'\text{-}Me}AH$ was less than 1%. Reaction conditions: 200 µL reaction containing 1.05 mM D/L-Hcy thiolactone, 1 mM 2'-*O*-methyladenosine, 1 µM *Ao*ACLR, 1 µM *Sc*BLH, 5 µM *Pa*SAHH or *Mm*SAHH and 50 mM sodium phosphate buffer (pH 8.0) was incubated at 25 °C for 20 h.

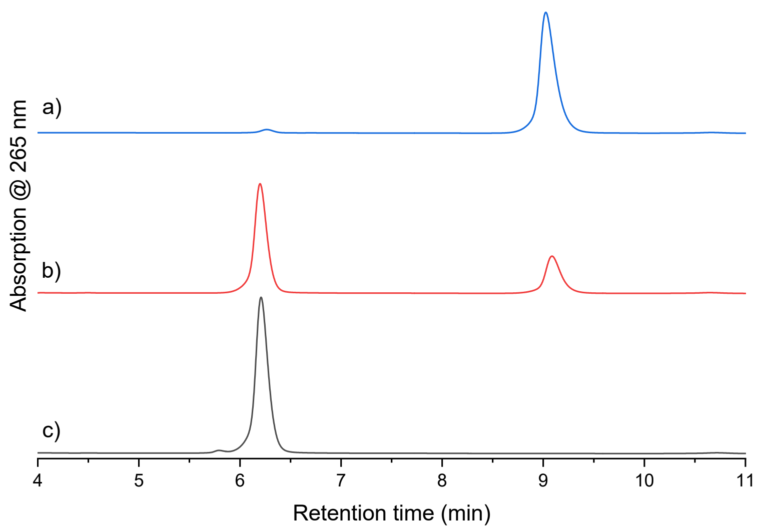

Entry	Retention time of N^6-methyladenosine (min)	Retention time of Sm⁶AH (min)	Peak area of N^6-methyladenosine	Peak area of Sm⁶AH	Yield of Sm⁶AH
a	6.26	9.03	942	45065	98%
b	6.20	9.09	32700	13640	29%

Figure S33. HPLC analysis monitoring synthesis of Sm^{6}AH by *Ao*ACLR-*Sc*BLH-SAHH enzyme cascade from D/L-Hcy thiolactone and N^6-methyladenosine. a) with *Mm*SAHH; b) with *Pa*SAHH; c) control: without enzymes. The yield of Sm^{6}AH = peak area of Sm^{6}AH / (peak area of N^6-methyladenosine + peak area of Sm^{6}AH) x 100%. Reaction conditions: 200 µL reaction containing 1.05 mM D/L-Hcy thiolactone, 1 mM N^6-methyladenosine, 1 µM *Ao*ACLR, 1 µM *Sc*BLH, 5 µM *Pa*SAHH or *Mm*SAHH and 50 mM sodium phosphate buffer (pH 8.0) was incubated at 25 °C for 20 h.

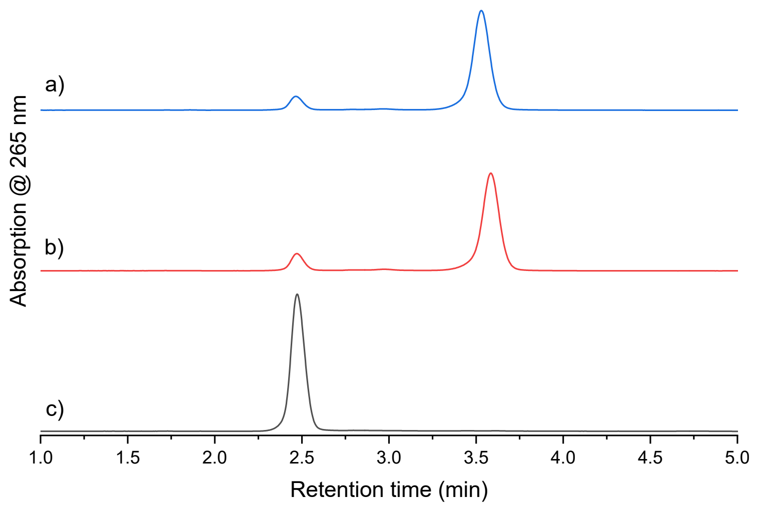

Entry	Retention time of inosine (min)	Retention time of SIH (min)	Peak area of inosine	Peak area of SIH	Yield of SIH
a	2.47	3.58	1592	15428	91%
b	2.47	3.53	1937	15127	89%

Figure S34. HPLC analysis monitoring synthesis of SIH by *Ao*ACLR-*Sc*BLH-SAHH enzyme cascade from D/L-Hcy thiolactone and inosine. a) with *Mm*SAHH; b) with *Pa*SAHH; c) control: without enzymes. The yield of SIH = peak area of SIH / (peak area of inosine + peak area of SIH) x 100%. Reaction conditions: 200 μL reaction containing 1.05 mM D/L-Hcy thiolactone, 1 mM inosine, 1 μM *Ao*ACLR, 1 μM *Sc*BLH, 5 μM *Pa*SAHH or *Mm*SAHH and 50 mM sodium phosphate buffer (pH 8.0) was incubated at 25 °C for 2 h.

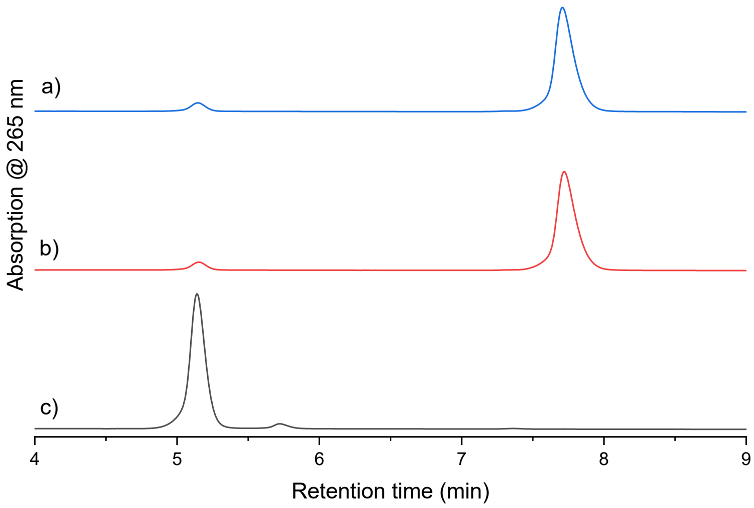

Entry	Retention time of 2-aminoadenosine (min)	Retention time of S2-aminoAH (min)	Peak area of 2-aminoadenosine	Peak area of S2-aminoAH	Yield of S2-aminoAH
a	5.15	7.71	1413	22579	94%
b	5.15	7.72	1328	21440	94%

Figure S35. HPLC analysis monitoring synthesis of S2-aminoAH by *Ao*ACLR-*Sc*BLH-SAHH enzyme cascade from D/L-Hcy thiolactone and 2-aminoadenosine. a) with *Mm*SAHH; b) with *Pa*SAHH; c) control: without enzymes. The yield of S2-aminoAH = peak area of S2-aminoAH / (peak area of 2-aminoadenosine + peak area of S2-aminoAH) x 100%. Reaction conditions: 200 µL reaction containing 1.05 mM D/L-Hcy thiolactone, 1 mM 2-aminoadenosine, 1 µM *Ao*ACLR, 1 µM *Sc*BLH, 5 µM *Pa*SAHH or *Mm*SAHH and 50 mM sodium phosphate buffer (pH 8.0) was incubated at 25 °C for 2 h.

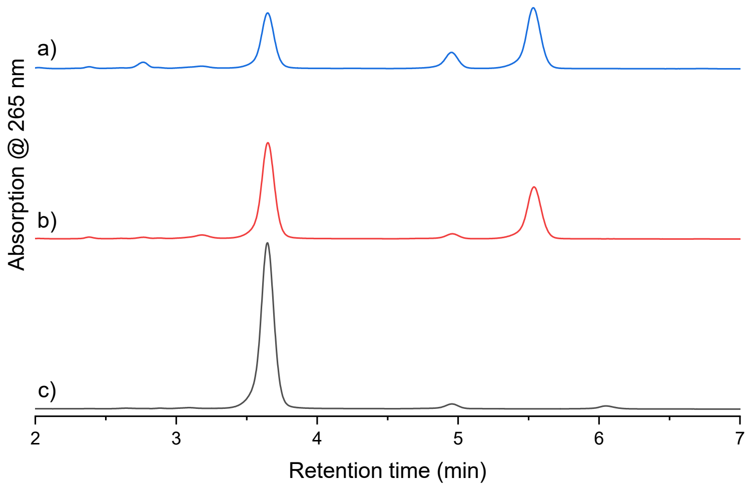

Entry	Retention time of guanosine (min)	Retention time of SGH (min)	Retention time of byproduct (min)	Peak area of guanosine	Peak area of SGH	Peak area of byproduct	Yield of SGH
a	3.65	5.53	4.95	9614	11742	2947	48%
b	3.65	5.54	4.96	16717	10030	916	36%

Figure S36. HPLC analysis monitoring synthesis of SGH by *Ao*ACLR-*Sc*BLH-SAHH enzyme cascade from D/L-Hcy thiolactone and guanosine. a) with *Mm*SAHH; b) with *Pa*SAHH; c) control: without enzymes. The yield of SGH = peak area of SGH / (peak area of guanosine + peak area of SGH + peak area of byproduct) x 100%. Reaction conditions: 200 µL reaction containing 1.05 mM D/L-Hcy thiolactone, 1 mM guanosine, 1 µM *Ao*ACLR, 1 µM *Sc*BLH, 20 µM *Pa*SAHH or *Mm*SAHH and 50 mM sodium phosphate buffer (pH 8.0) was incubated at 25 °C for 44 h.

Characterization of SAH derivatives

A 2 mL reaction containing 5.25 mM D/L-Hcy thiolactone hydrochloride, 5 mM adenosine analogs, 5 µM *Ao*ACLR, 5 µM *Sc*BLH, 20 or 60 µM *Pa*SAHH or *Mm*SAHH, and 50 mM sodium phosphate buffer (pH 8.0) were incubated at 25 °C. After 20 hours, the reaction was quenched by vigorously mixing with 2 mL of MeCN to precipitate enzymes. The denatured enzymes were removed by centrifugation. The supernatant was collected and purified by preparative reverse-phase HPLC (Column: Reprospher 100 C18-DE, particle size: 5 µm, length: 150 mm, inner diameter: 40 mm. Flow rate: 20 mL/min. Conditions: Buffer A: milliQ water; Buffer B: MeCN. 0-10 mins: 3% Buffer B; 10-25 mins: gradient of 3-30% Buffer B; 25-30 mins: gradient of 30-100% Buffer B; 30-38 mins: 100% Buffer B; 38-40 mins: gradient of 100-3% Buffer B). The eluted fractions were collected and lyophilized to give the product SAH derivatives.

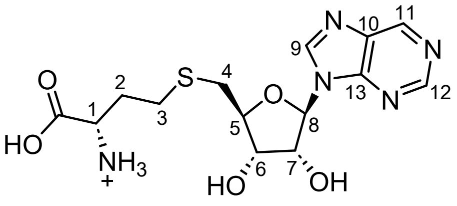

S-nebularylhomocysteine (SNH)

S-Nebularylhomocysteine (SNH) from nebularine. 20 µM *Pa*SAHH was used.

^{1}H NMR (500 MHz, D_2O) δ 9.16 (s, 1H, H-11), 8.98 (s, 1H, H-12), 8.71 (s, 1H, H-9), 6.23 (d, *J* = 5.0 Hz, 1H, H-8), 4.98 (t, *J* = 5.2 Hz, 1H, H-7), 4.50 (t, *J* = 5.1 Hz, 1H, H-5), 4.37 (dt, *J* = 6.9, 4.9 Hz, 1H, H-6), 3.74–3.65 (m, 1H, H-1), 3.10 (dd, *J* = 14.1, 5.0 Hz, 1H, H-4a), 3.03 (dd, *J* = 14.1, 6.9 Hz, 1H, H-4b), 2.68 (t, *J* = 7.6 Hz, 2H, H-3), 2.11 (dq, *J* = 14.0, 7.2 Hz, 1H, H-2a), 2.01 (dq, *J* = 14.5, 7.3 Hz, 1H, H-2b).

^{13}C NMR (126 MHz, D_2O) δ 175.9 ($\underline{C}O_2H$), 152.1 (C-12), 150.8 (C-13), 148.0 (C-11), 145.7 (C-9), 133.7 (C-10), 88.0 (C-8), 83.5 (C-6), 73.2 (C-7), 72.4 (C-5), 54.0 (C-1), 33.5 (C-4), 31.3 (C-2), 27.9 (C-3).

HRMS (ESI) Cald. for $C_{14}H_{20}N_5O_5S^+$ ($M+H^+$) 370.1180, found 370.1184.

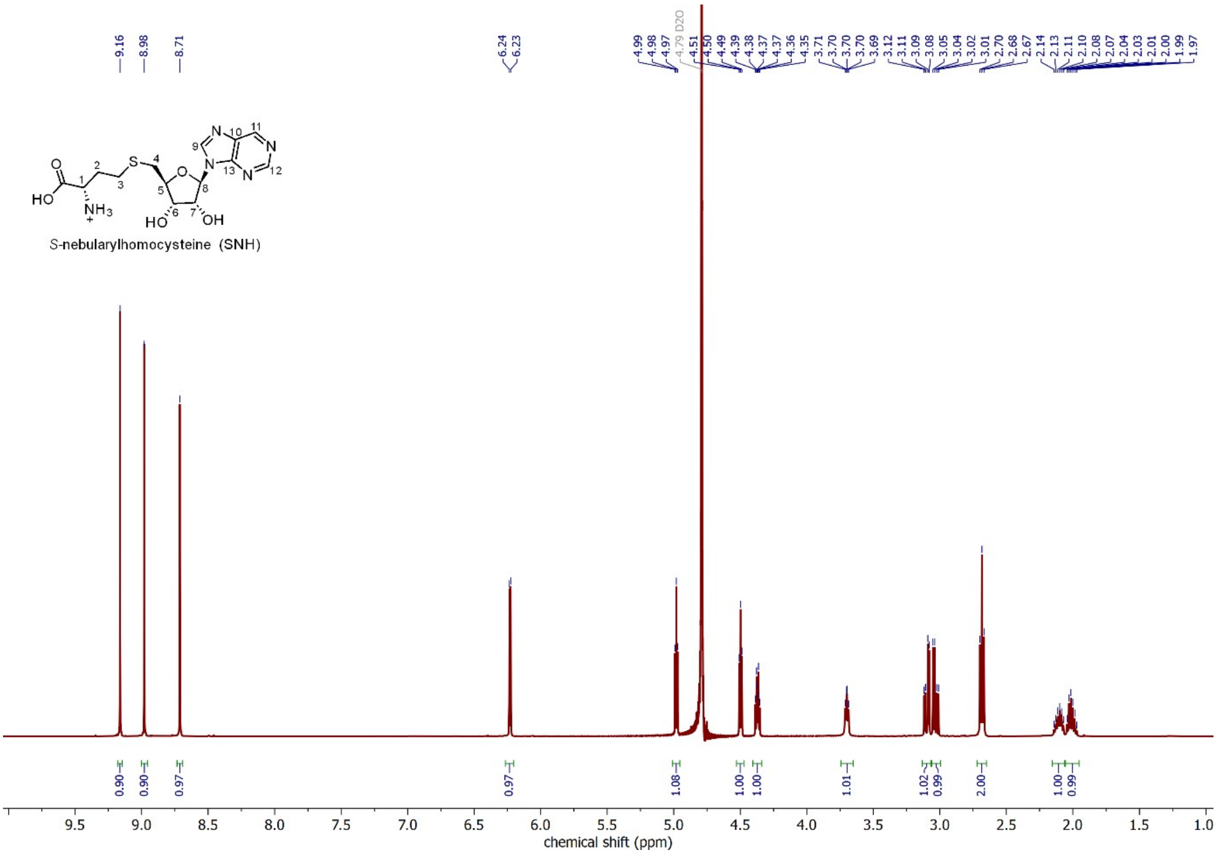

Figure S37. ^{1}H NMR spectrum of SNH.

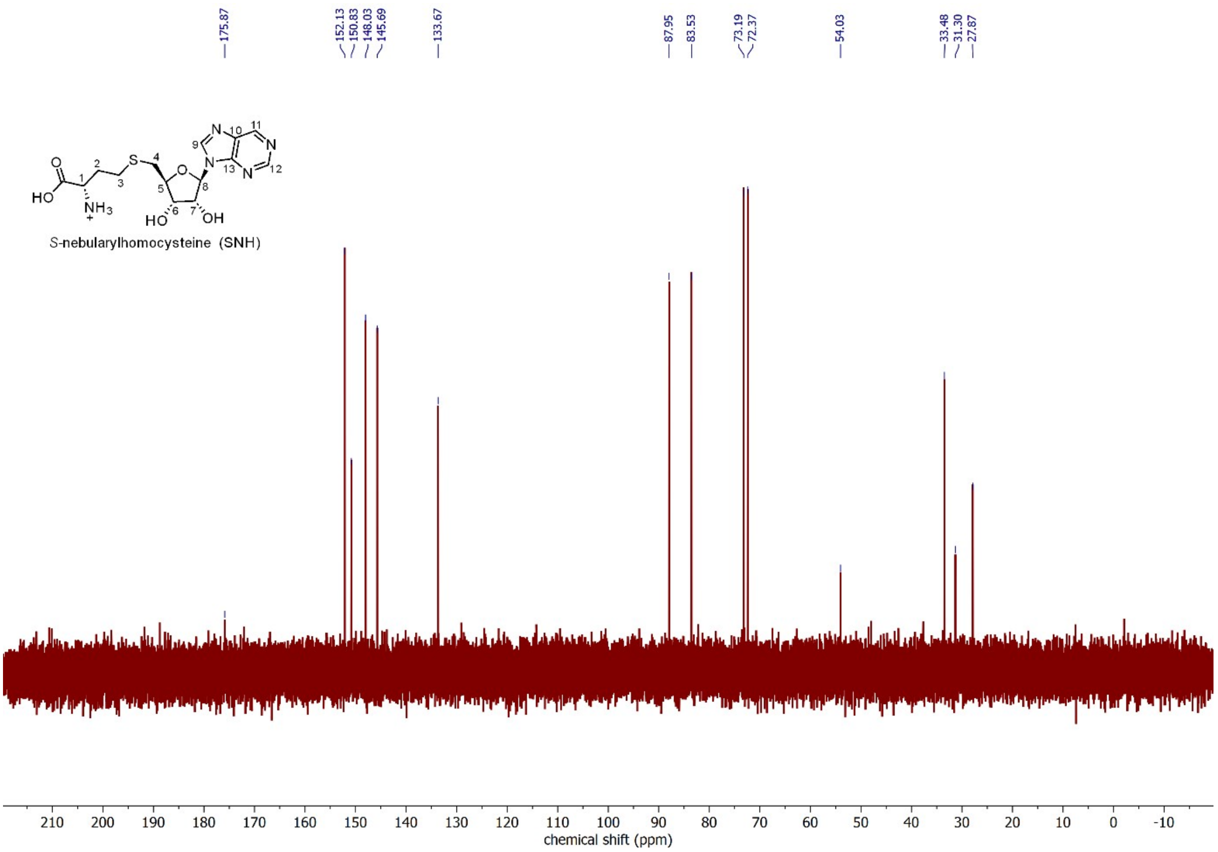

Figure S38. ^{13}C NMR spectrum of SNH.

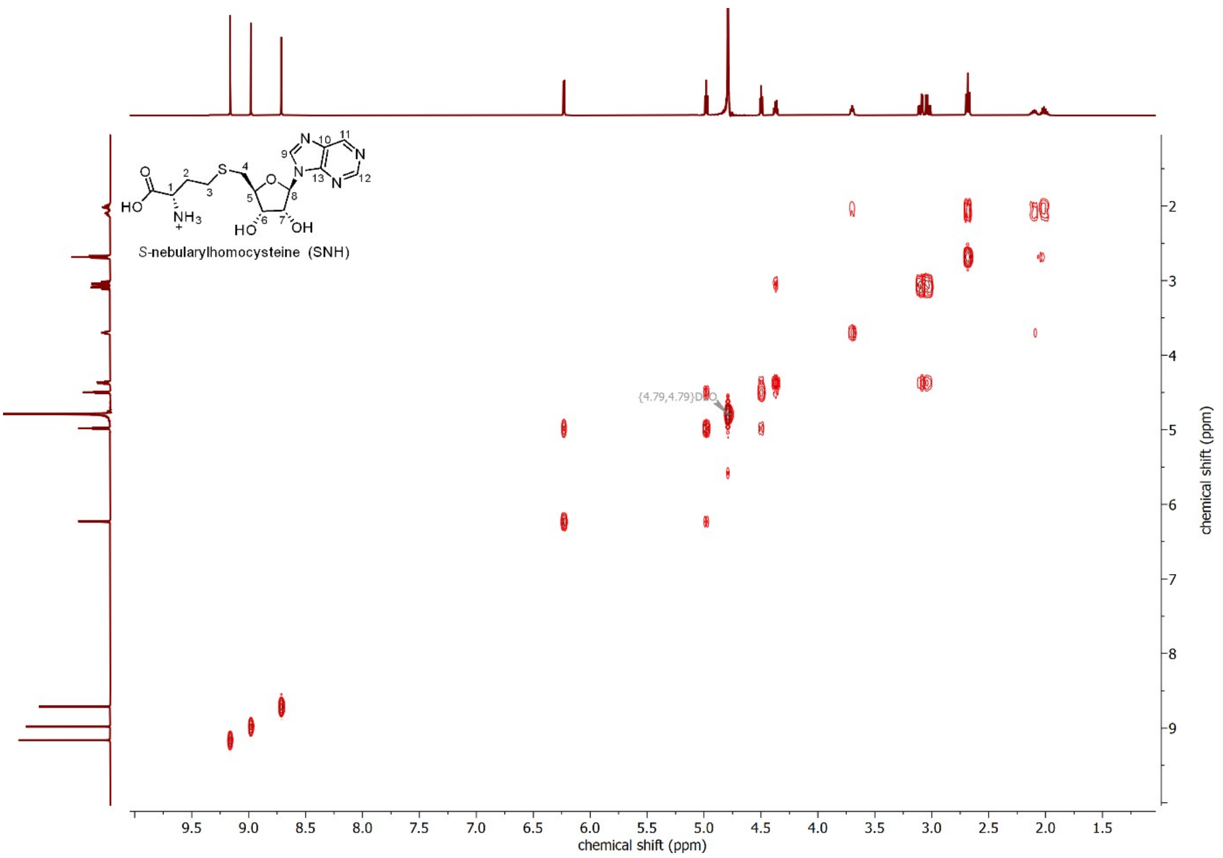

Figure S39. ^{1}H-^{1}H COSY spectrum of SNH.

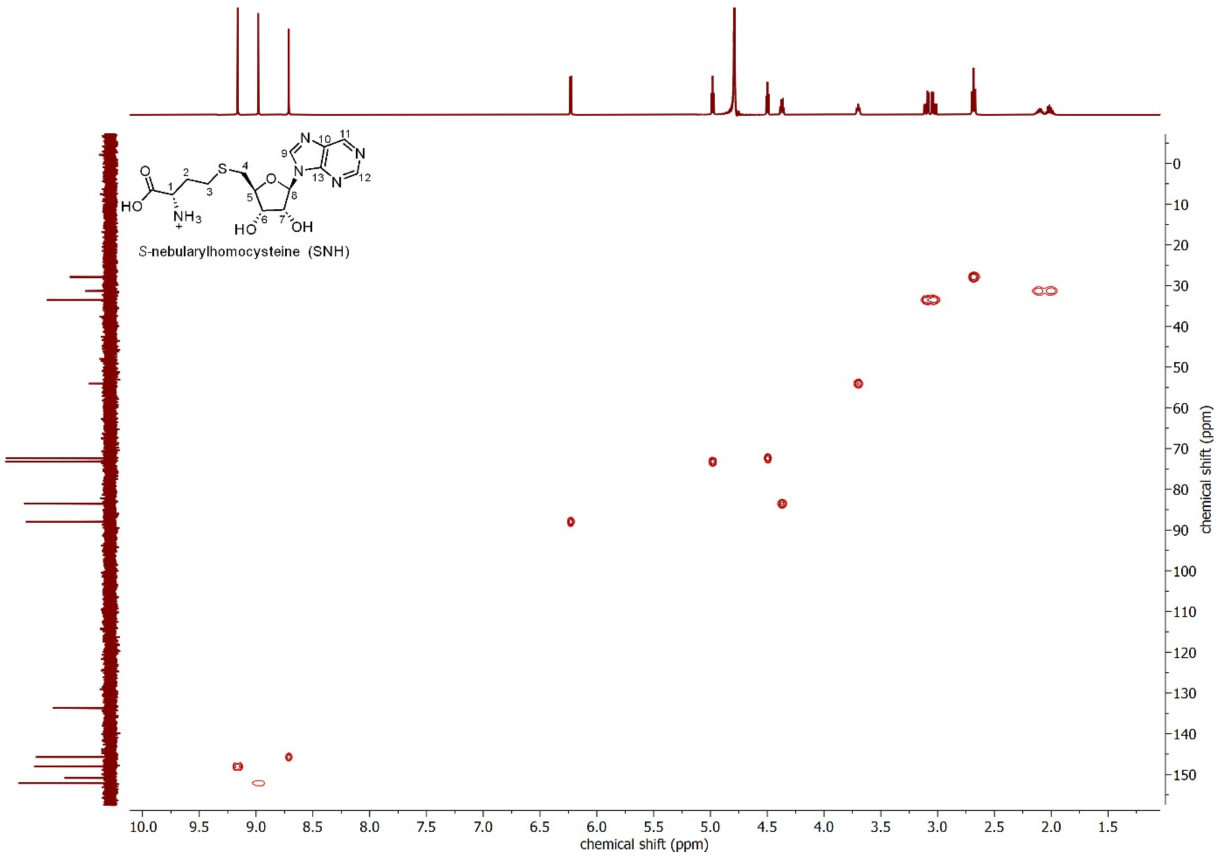

Figure S40. HSQC spectrum of SNH.

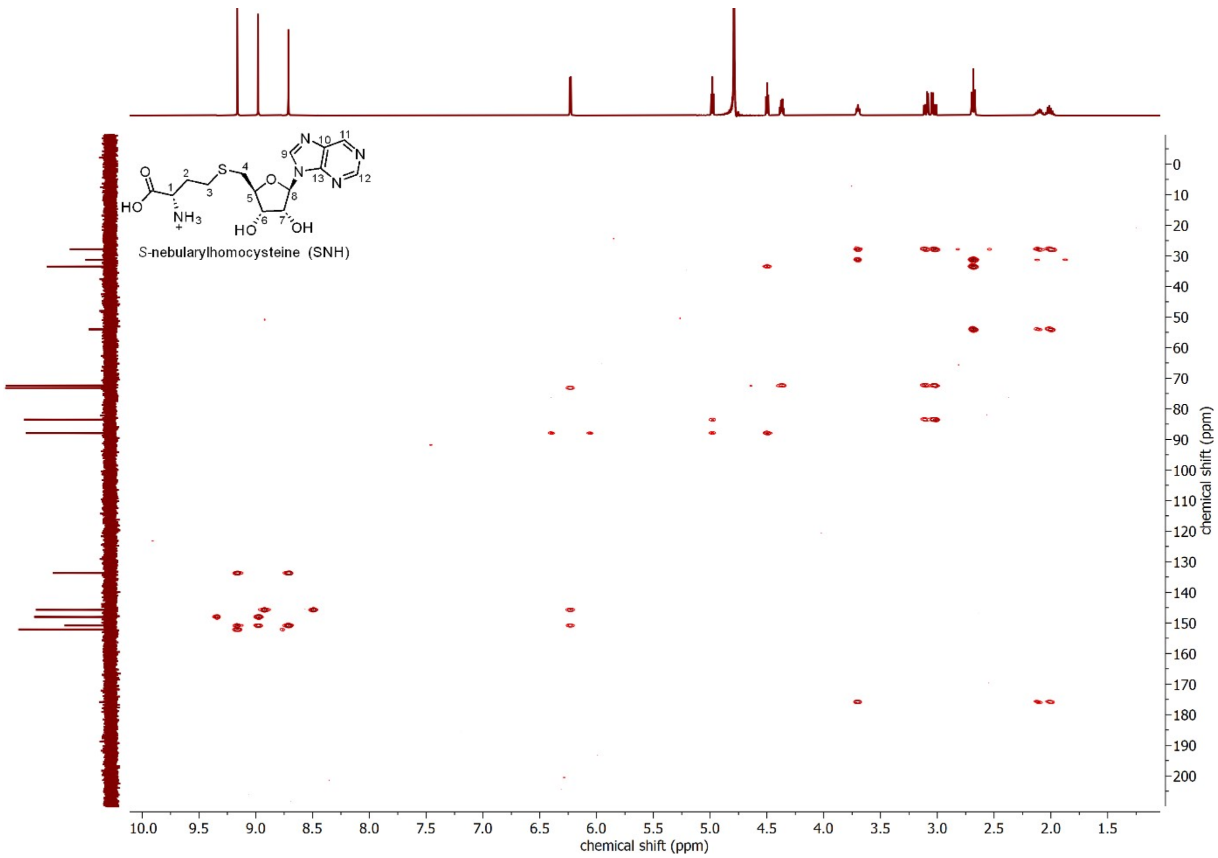

Figure S41. HMBC spectrum of SNH.

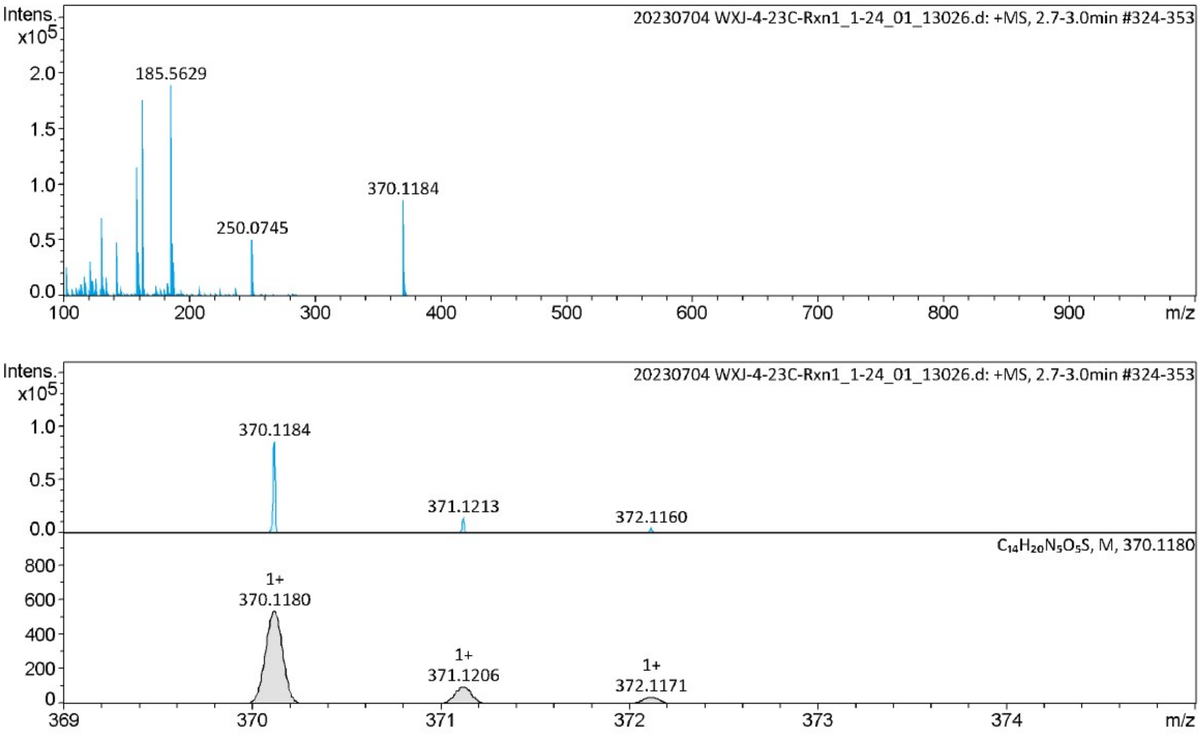

Figure S42. Measured and calculated HR-ESI-MS spectra of SNH.

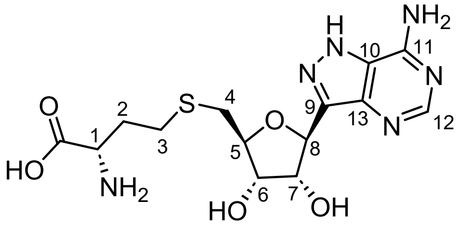

S-formycinylhomocysteine (SFH)

S-Formycinylhomocysteine (SFH) from formycin A. 20 µM *Pa*SAHH was used.[5]

^{1}H NMR (500 MHz, D_2O) δ 8.18 (s, 1H, H-12), 5.26 (d, *J* = 6.8 Hz, 1H, H-8), 4.80 (1H, H-7, assigned by 2D NMR), 4.33 (dd, *J* = 5.7, 4.5 Hz, 1H, H-5), 4.22 (dt, *J* = 7.0, 4.9 Hz, 1H, H-6), 3.67–3.57 (m, 1H, H-1), 2.99 (dd, *J* = 13.9, 5.2 Hz, 1H, H-4a), 2.89 (dd, *J* = 14.0, 7.1 Hz, 1H, H-4b), 2.74–2.61 (m, 2H, H-3), 2.13–2.01 (m, 1H, H-2a), 2.00–1.89 (m, 1H, H-2b).

^{13}C NMR (126 MHz, D_2O) δ 177.0 ($\underline{C}O_2H$, assigned by 2D NMR), 152.4 (C-11), 151.5 (C-12), 139.9 (C-9, assigned by 2D NMR), 138.3 (C-13, assigned by 2D NMR), 83.6 (C-6), 76.5 (C-8), 73.7 (C-5), 73.5 (C-7), 54.3 (C-1), 33.8 (C-4), 31.9 (C-2, assigned by 2D NMR), 28.0 (C-3).

HRMS (ESI) Cald. for $C_{14}H_{21}N_6O_5S^+$ ($M+H^+$) 385.1289, found 385.1291.

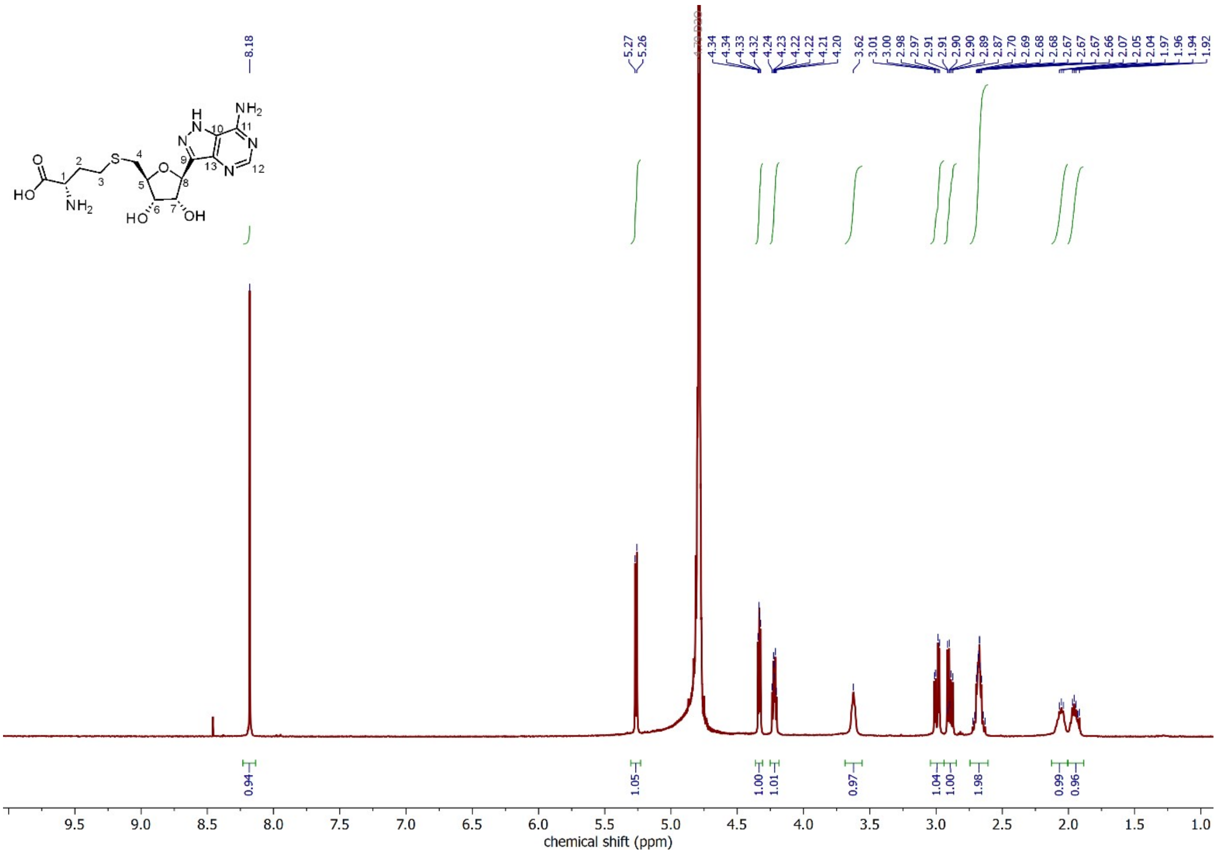

Figure S43. ^{1}H NMR spectrum of SFH.

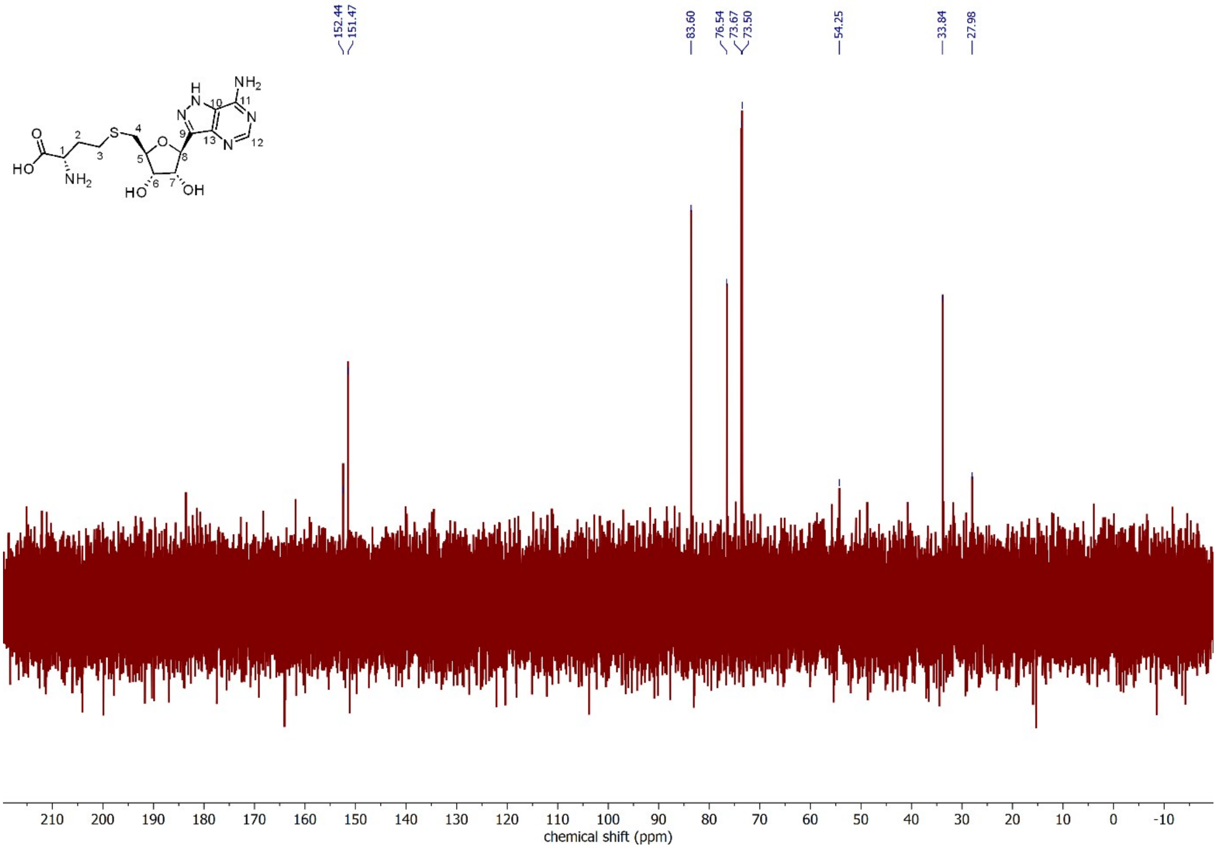

Figure S44. ^{13}C NMR spectrum of SFH.

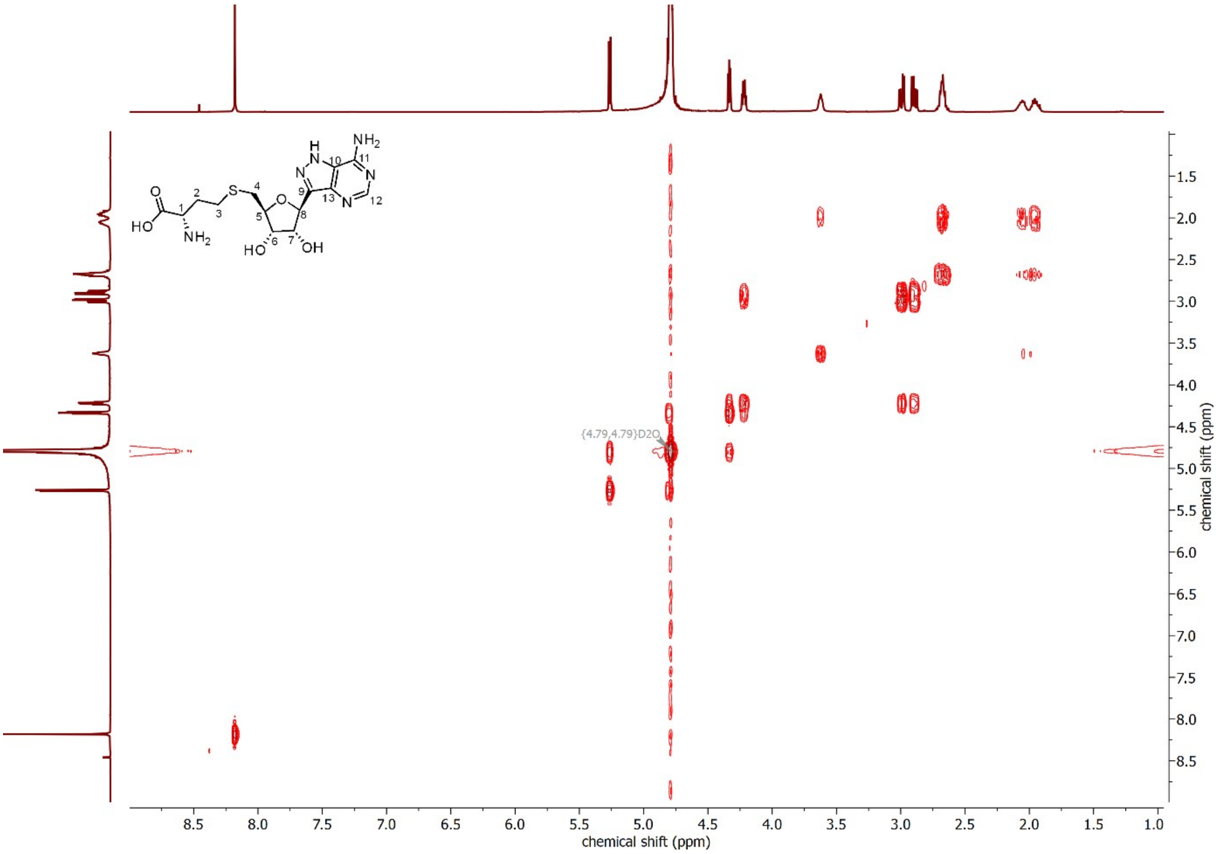

Figure S45. ^{1}H-^{1}H COSY spectrum of SFH.

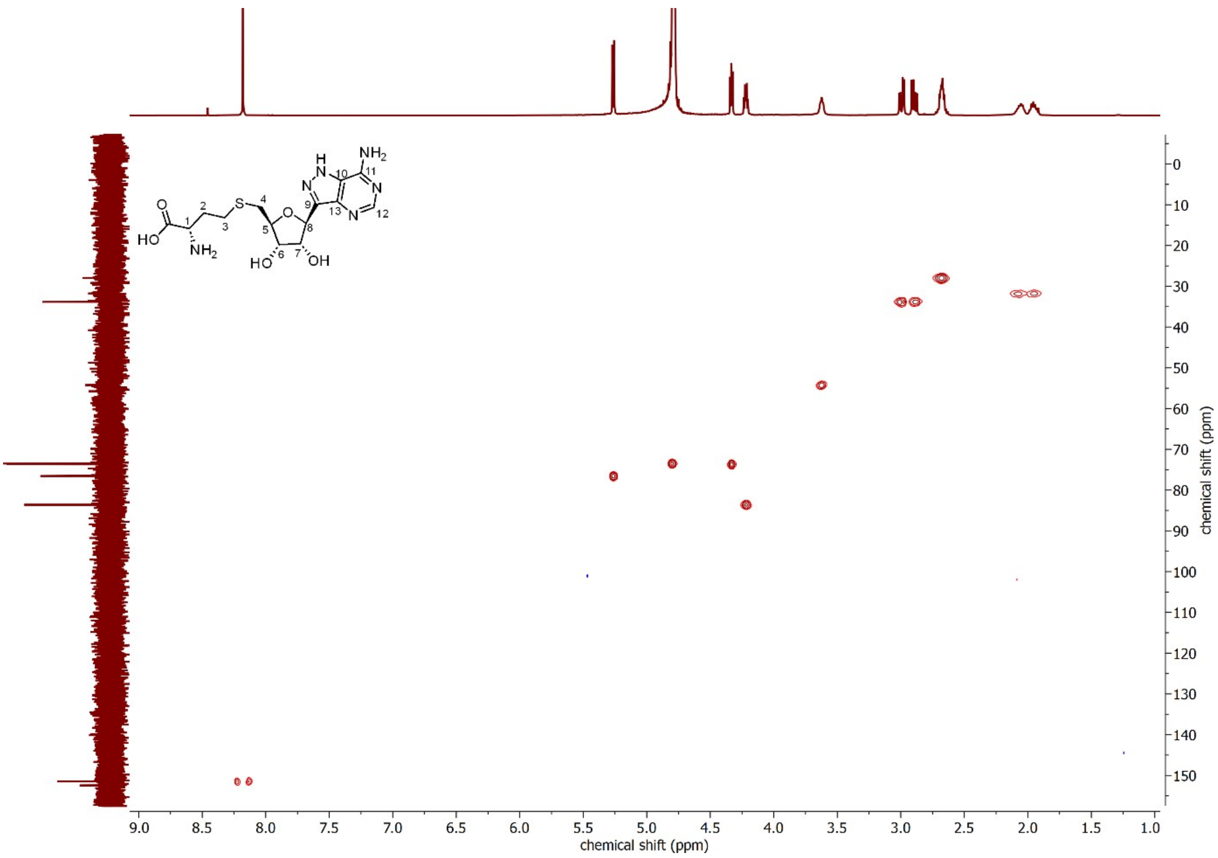

Figure S46. HSQC spectrum of SFH.

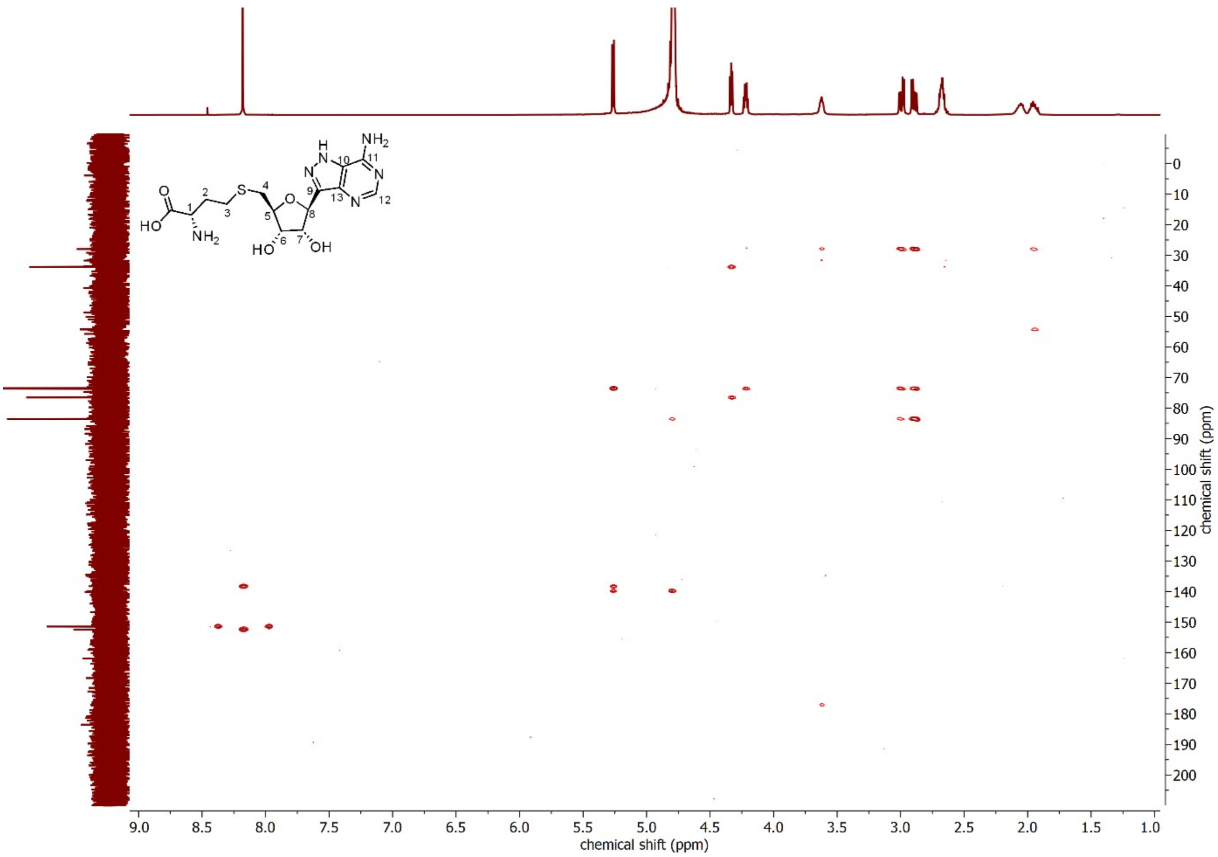

Figure S47. HMBC spectrum of SFH.

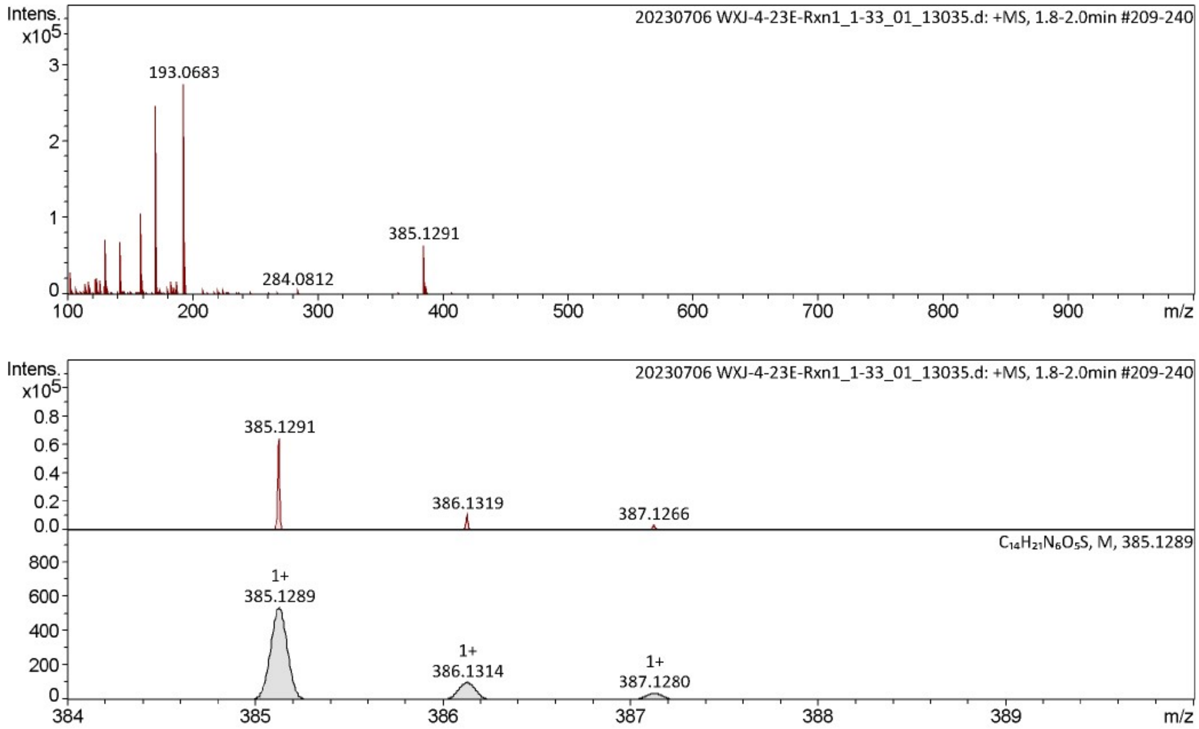

Figure S48. Measured and calculated HR-ESI-MS spectra of SFH.

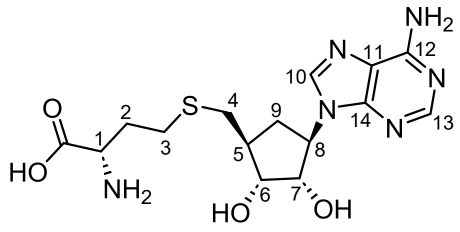

S-aristeromycinylhomocysteine (SArH)

S-Aristeromycinylhomocysteine (SArH) from aristeromycin.[6] 60 µM *Pa*SAHH was used.

^{1}H NMR (500 MHz, D_2O) δ 8.45 (s, 1H, H-10), 8.42 (s, 1H, H-13), 4.92 (dt, *J* = 10.9, 8.3 Hz, 1H, H-8), 4.58 (dd, *J* = 8.8, 5.9 Hz, 1H, H-7), 4.18 (dd, *J* = 6.7, 5.9 Hz, 1H, H-1), 4.12 (dd, *J* = 5.9, 4.0 Hz, 1H, H-5), 2.92 (dd, *J* = 13.0, 7.3 Hz, 1H, H-4a), 2.86–2.75 (m, 3H, H-3&4b), 2.61 (dt, *J* = 13.2, 8.1 Hz, 1H, H-9a), 2.40–2.27 (m, 2H, H-2a&6), 2.26–2.17 (m, 1H, H-2b), 1.93 (ddd, *J* = 13.3, 10.9, 9.2 Hz, 1H, H-9b).

^{13}C NMR (126 MHz, D_2O) δ 172.2 ($\underline{C}O_2H$), 149.8 (C-12), 148.9 (C-14), 143.8 (C-13), 143.5 (C-10), 118.6 (C-11), 75.0 (C-7), 73.7 (C-5), 60.3 (C-8), 52.1 (C-1), 42.4 (C-6), 34.5 (C-4), 31.5 (C-9), 29.7 (C-2), 26.8 (C-3).

HRMS (ESI) Cald. for $C_{15}H_{23}N_6O_4S^+$ ($M+H^+$) 383.1496, found 383.1502.

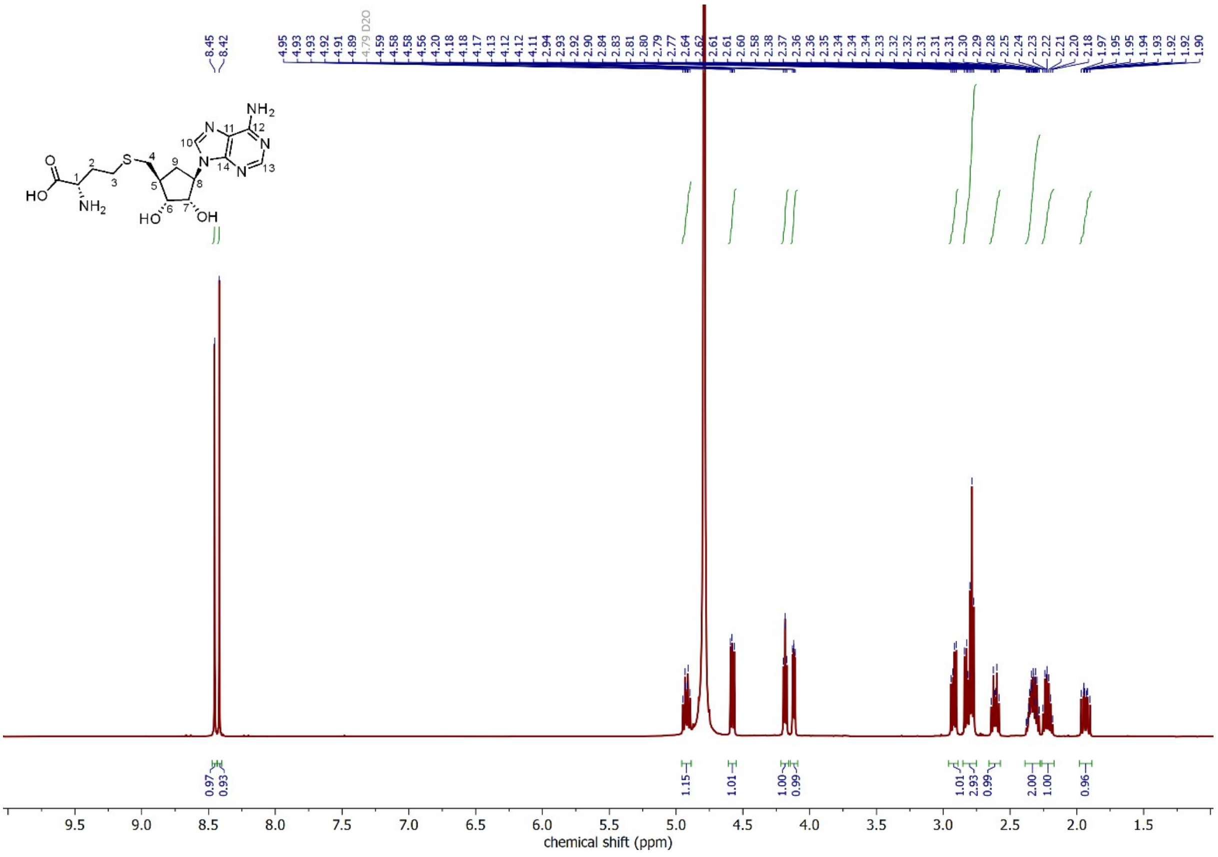

Figure S49. ^{1}H NMR spectrum of SArH.

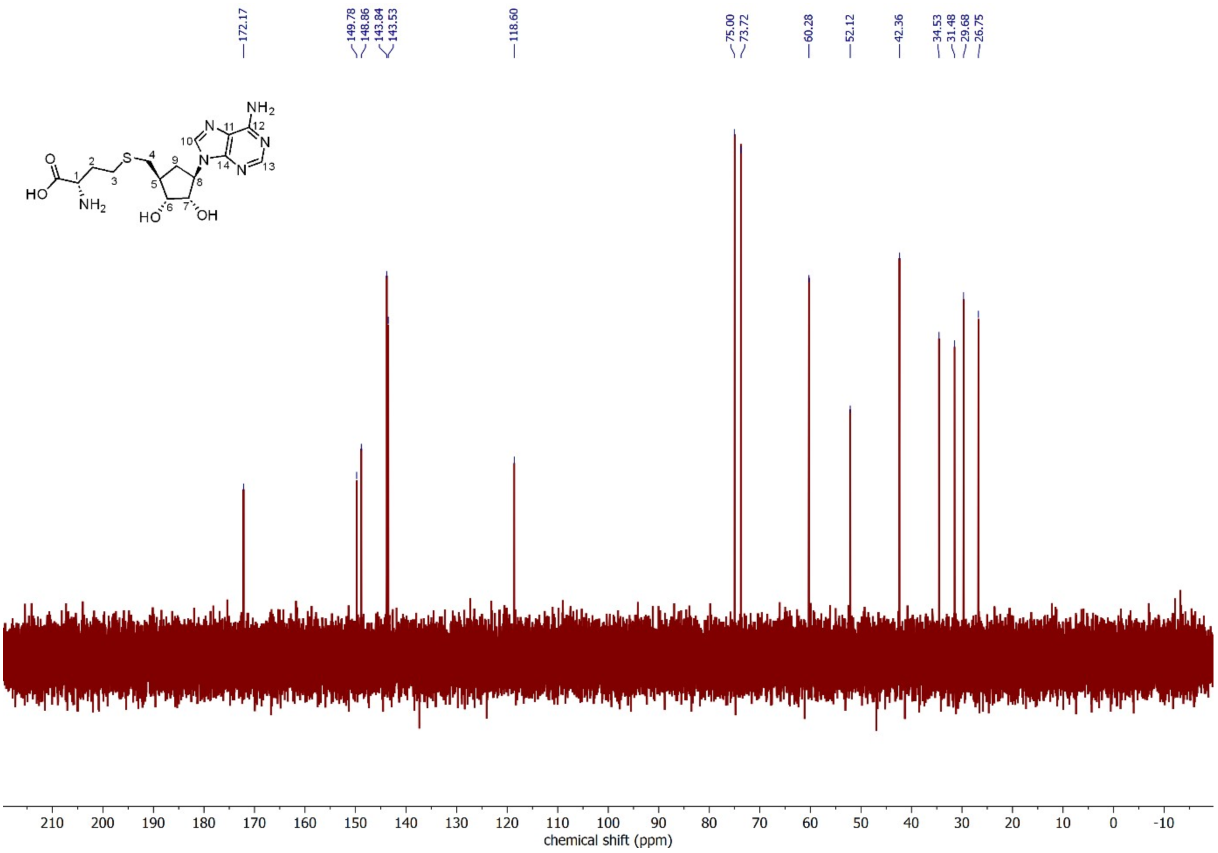

Figure S50. ^{13}C NMR spectrum of SArH.

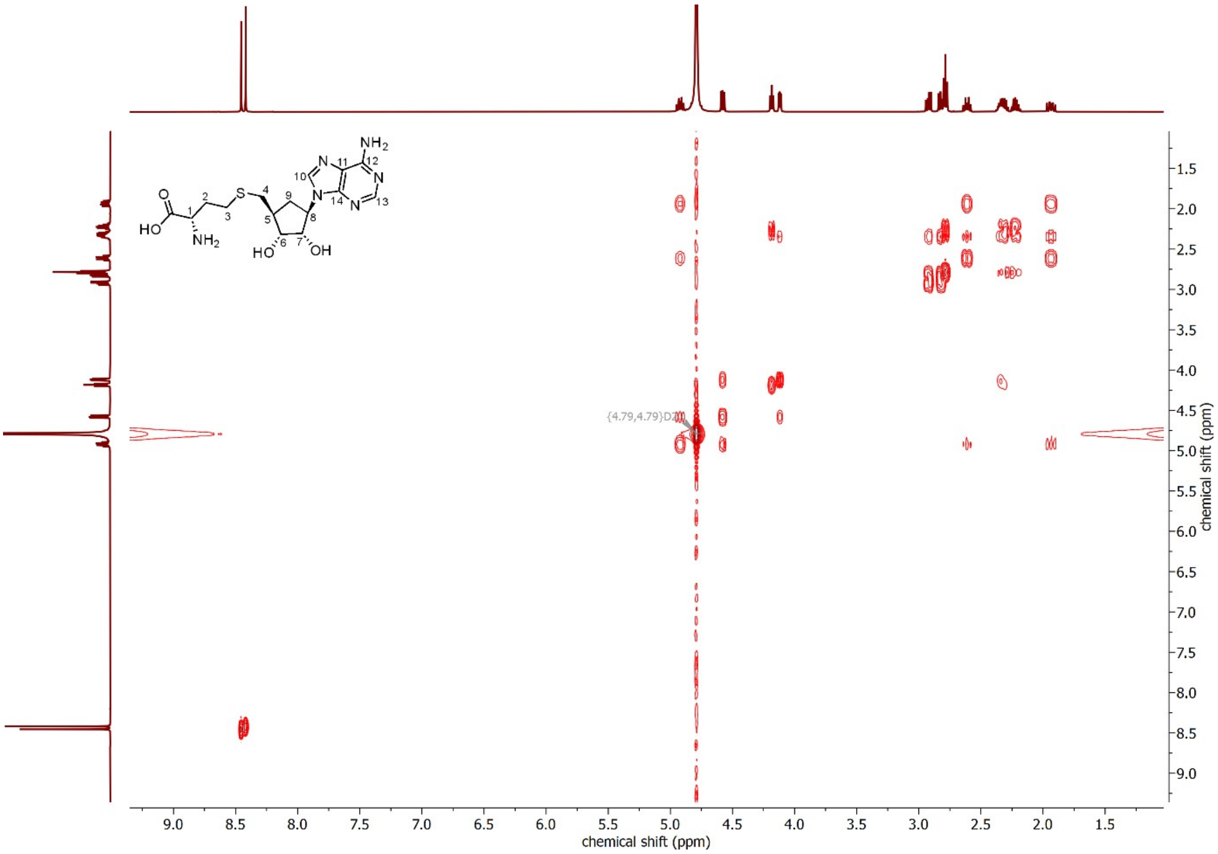

Figure S51. ^{1}H-^{1}H COSY spectrum of SArH.

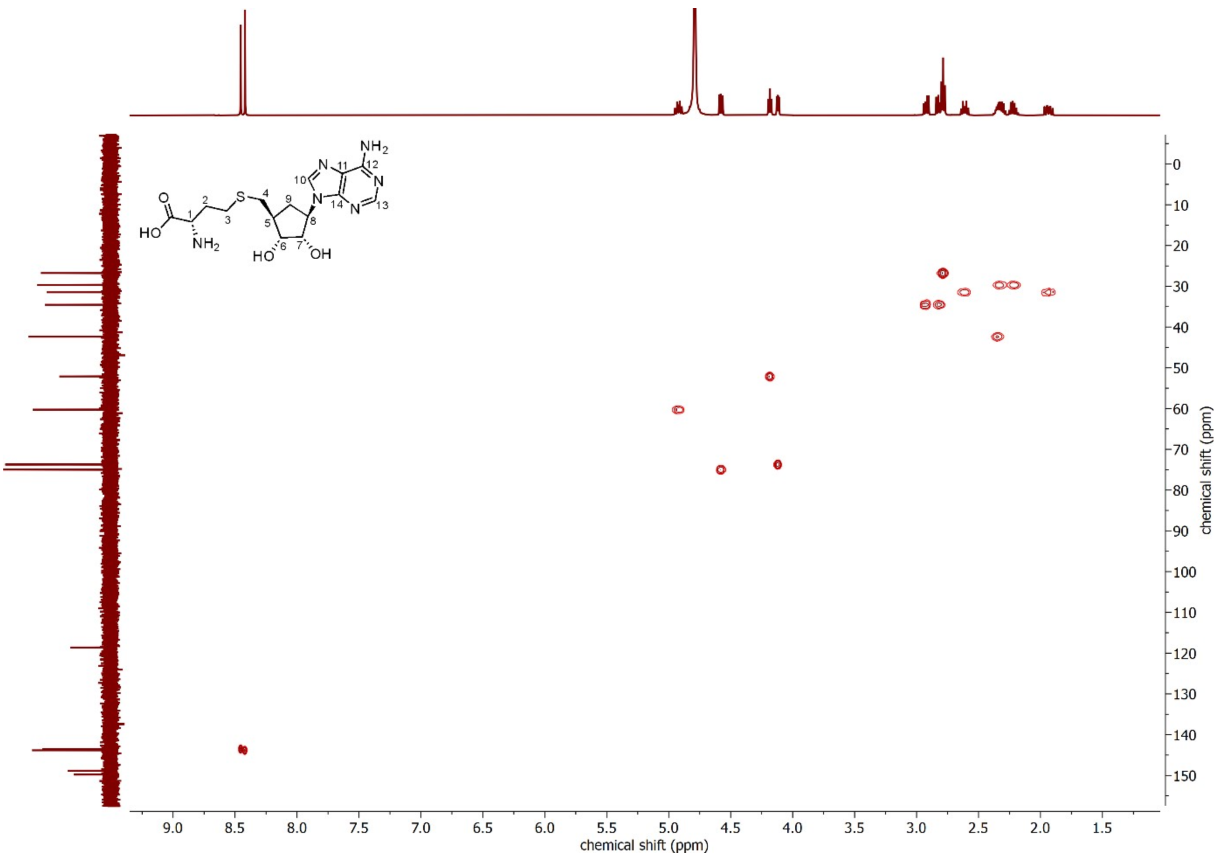

Figure S52. HSQC spectrum of SArH.

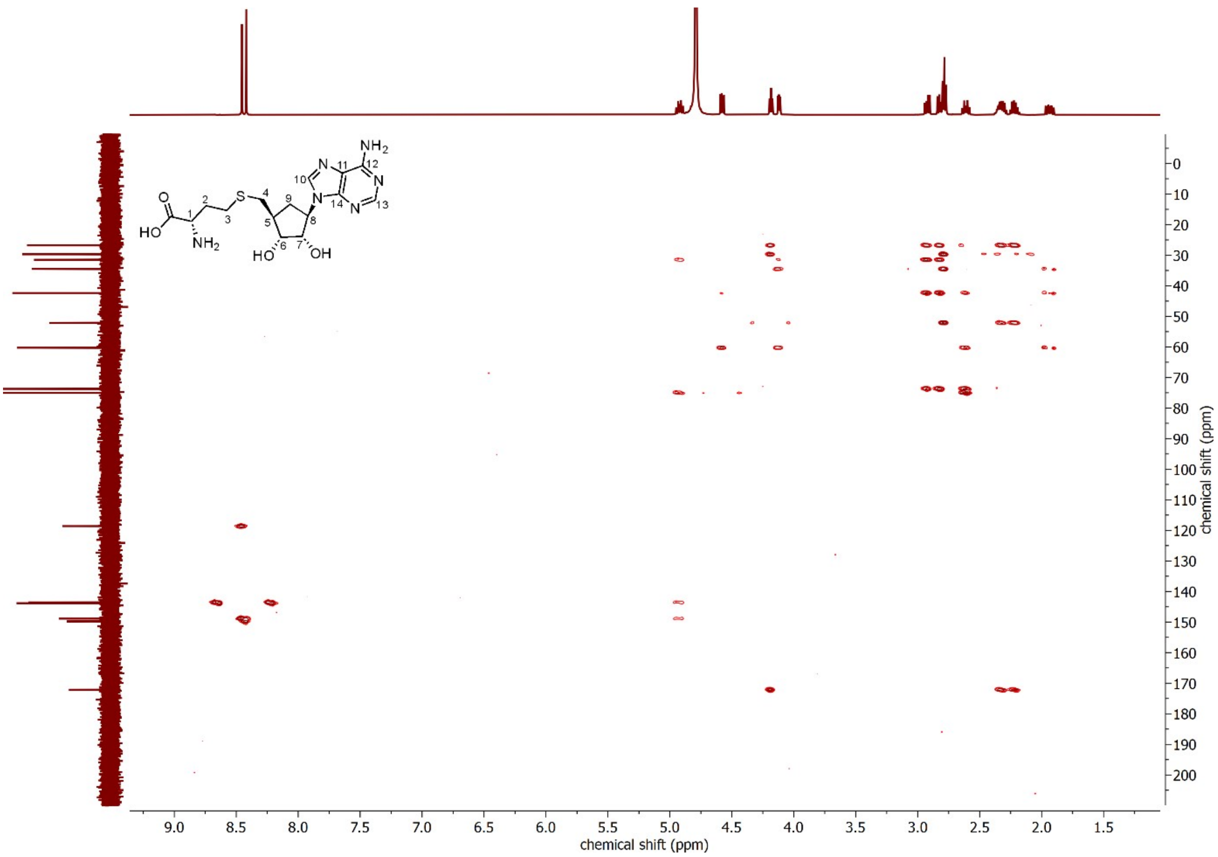

Figure S53. HMBC spectrum of SArH.

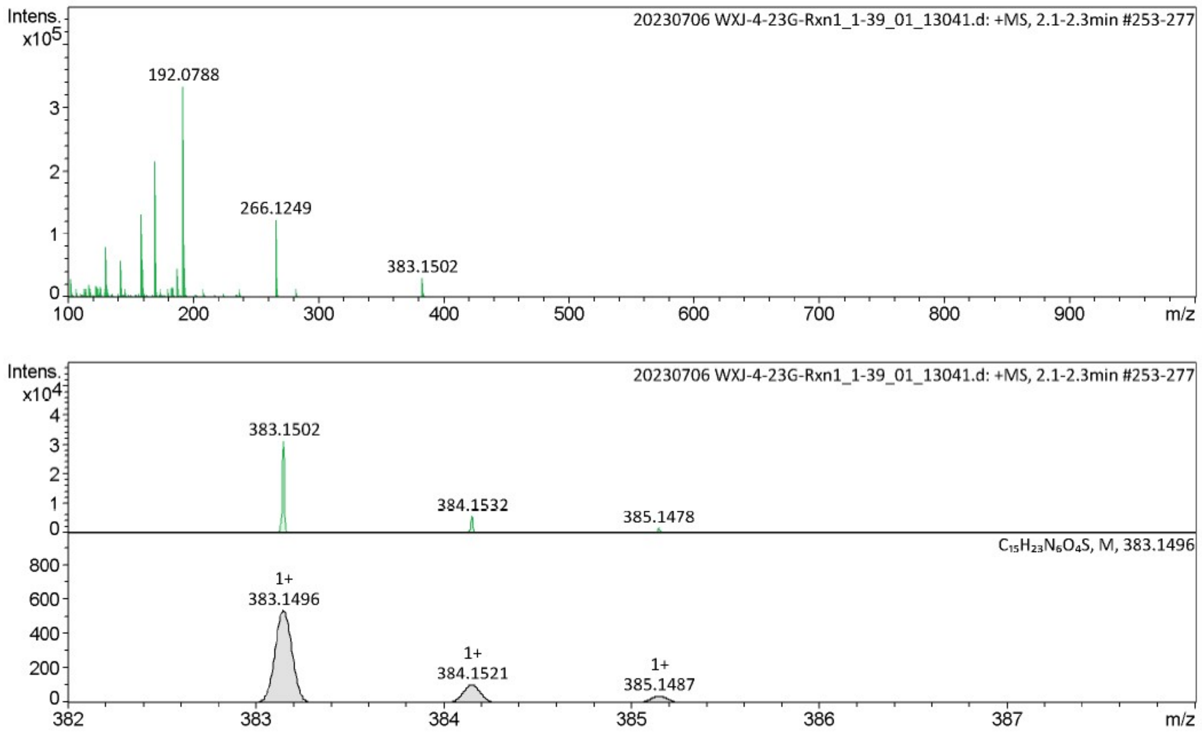

Figure S54. Measured and calculated HR-ESI-MS spectra of SArH.

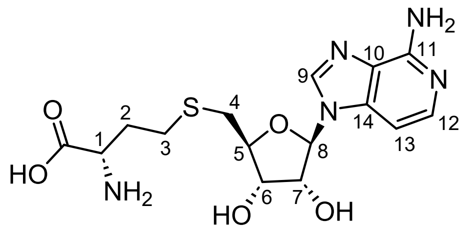

S-3-deazaadenosylhomocysteine ($S^{3\text{-deaza}}AH$)

S-3-Deazaadenosylhomocysteine ($S^{3\text{-deaza}}AH$) from 3-deazaadenosine.[7,8] 20 μM *Pa*SAHH was used.

^{1}H NMR (500 MHz, D_2O) δ 8.32 (s, 1H, H-9), 7.79 (d, J = 6.1 Hz, 1H, H-12), 7.06 (d, J = 6.1 Hz, 1H, H-13), 5.99 (d, J = 5.6 Hz, 1H, H-8), 4.69 (t, J = 5.6 Hz, 1H, H-7), 4.38 (dd, J = 5.5, 4.4 Hz, 1H, H-5), 4.33 (dt, J = 6.5, 4.8 Hz, 1H, H-6), 3.65 (t, J = 6.3 Hz, 1H, H-1), 3.06 (dd, J = 14.2, 5.1 Hz, 1H, H-4a), 2.96 (dd, J = 14.1, 6.5 Hz, 1H, H-4b), 2.68 (dd, J = 8.3, 7.0 Hz, 2H, H-3), 2.08 (dq, J = 14.1, 7.3 Hz, 1H, H-2a), 1.98 (dq, J = 14.5, 7.3 Hz, 1H, H-2b).

^{13}C NMR (126 MHz, D_2O) δ 176.5 ($\underline{C}O_2H$, assigned by 2D NMR), 151.4 (C-11), 141.2 (C-9), 140.4 (C-12), 138.3 (C-14), 126.7 (C-10), 99.2 (C-13), 88.9 (C-8), 83.5 (C-6), 73.2 (C-7), 72.1 (C-5), 54.1 (C-1), 33.6 (C-4), 31.6 (C-2), 28.1 (C-3).

HRMS (ESI) Cald. for $C_{15}H_{22}N_5O_5S^+$ ($M+H^+$) 384.1336, found 384.1342.

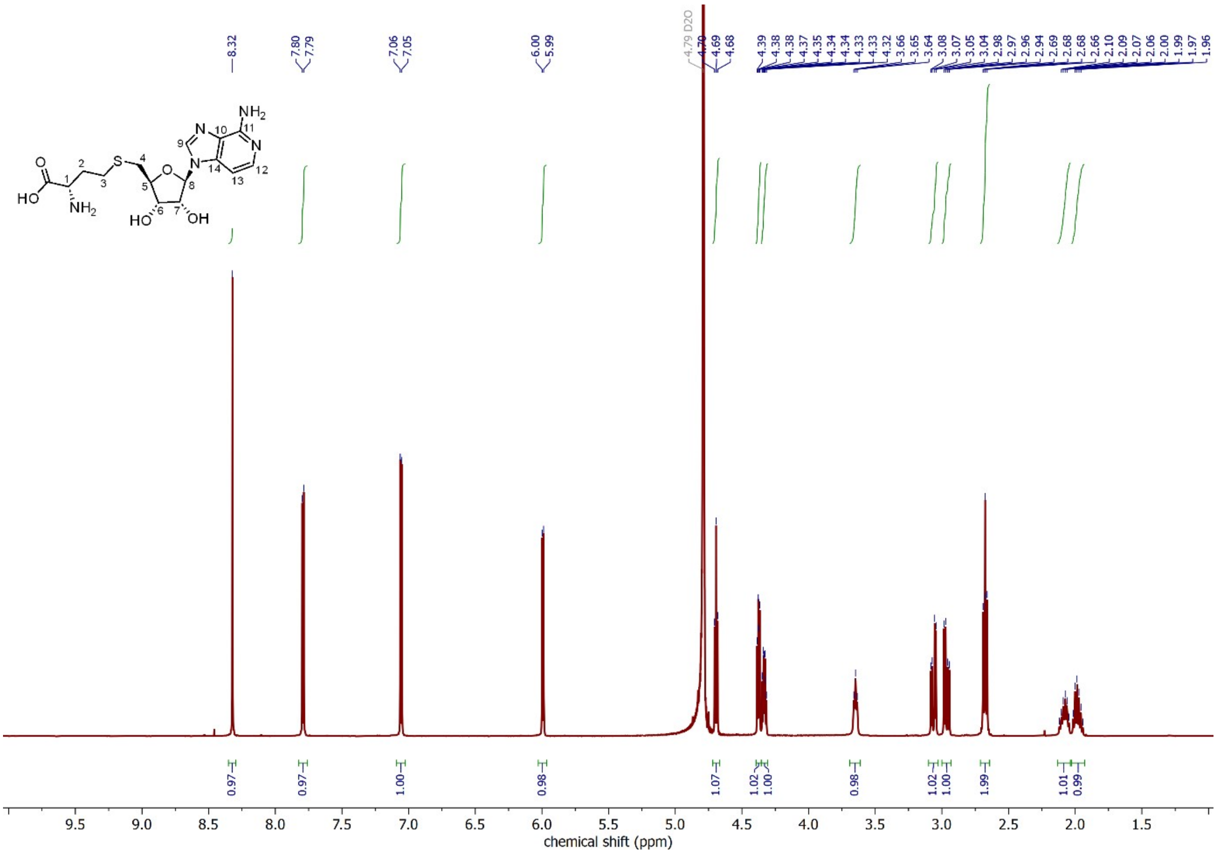

Figure S55. ^{1}H NMR spectrum of $S^{3\text{-deaza}}AH$.

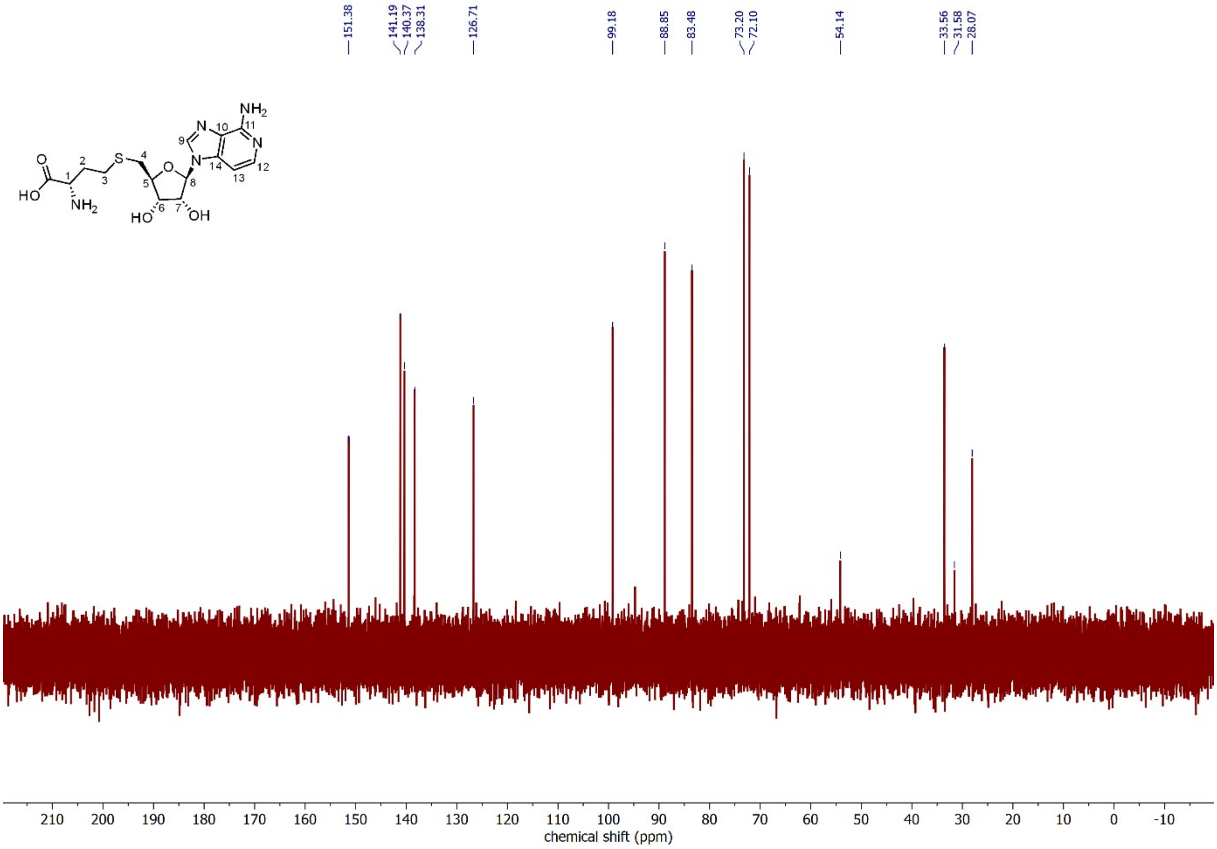

Figure S56. ^{13}C NMR spectrum of S$^{3\text{-deaza}}$AH.

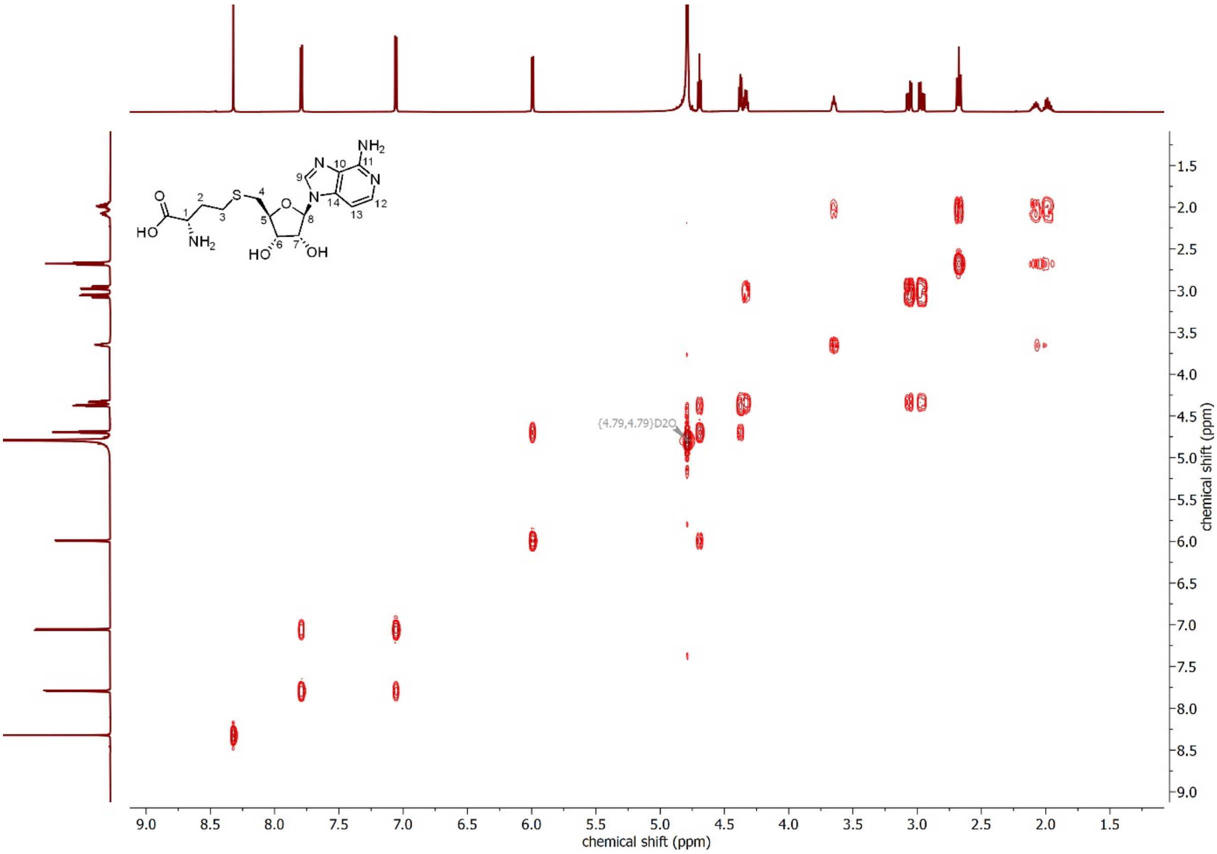

Figure S57. ^{1}H-^{1}H COSY spectrum of S$^{3\text{-deaza}}$AH.

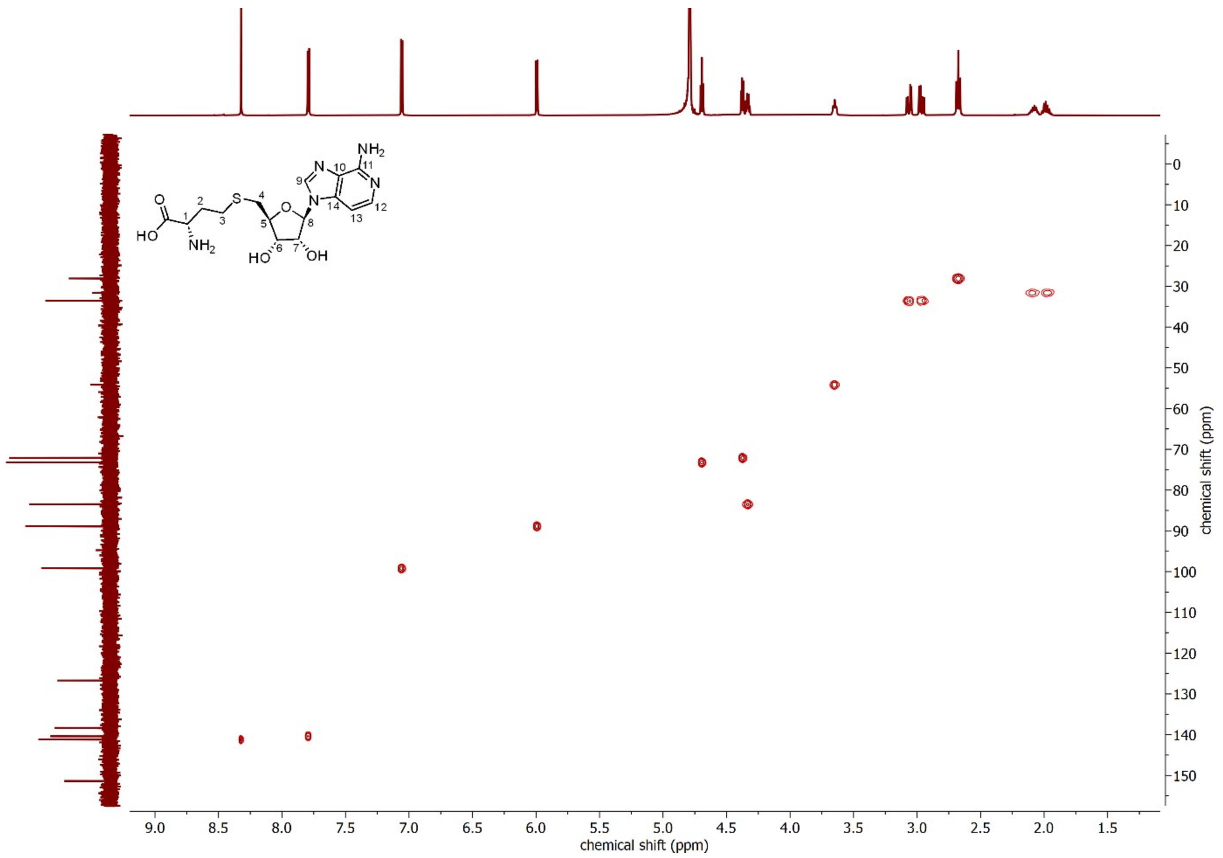

Figure S58. HSQC spectrum of S$^{3\text{-deaza}}$AH.

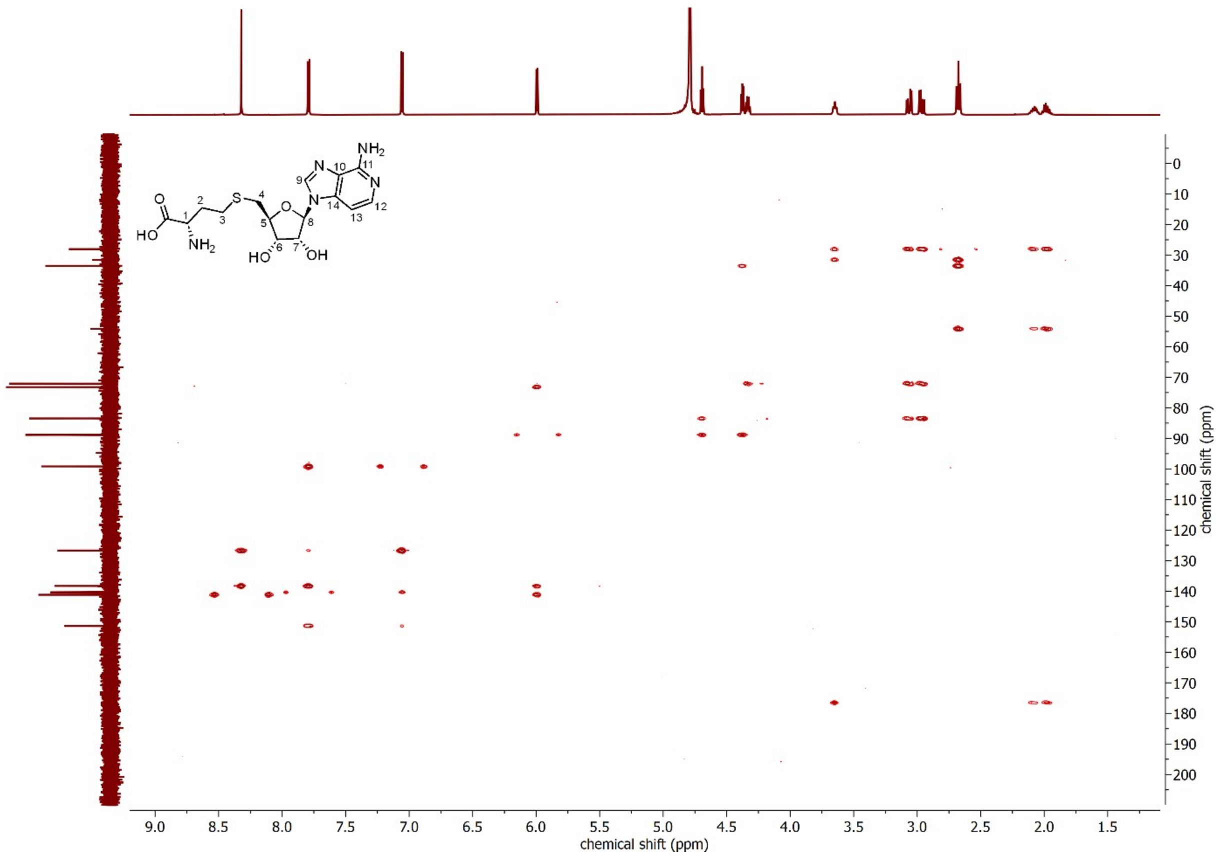

Figure S59. HMBC spectrum of S$^{3\text{-deaza}}$AH.

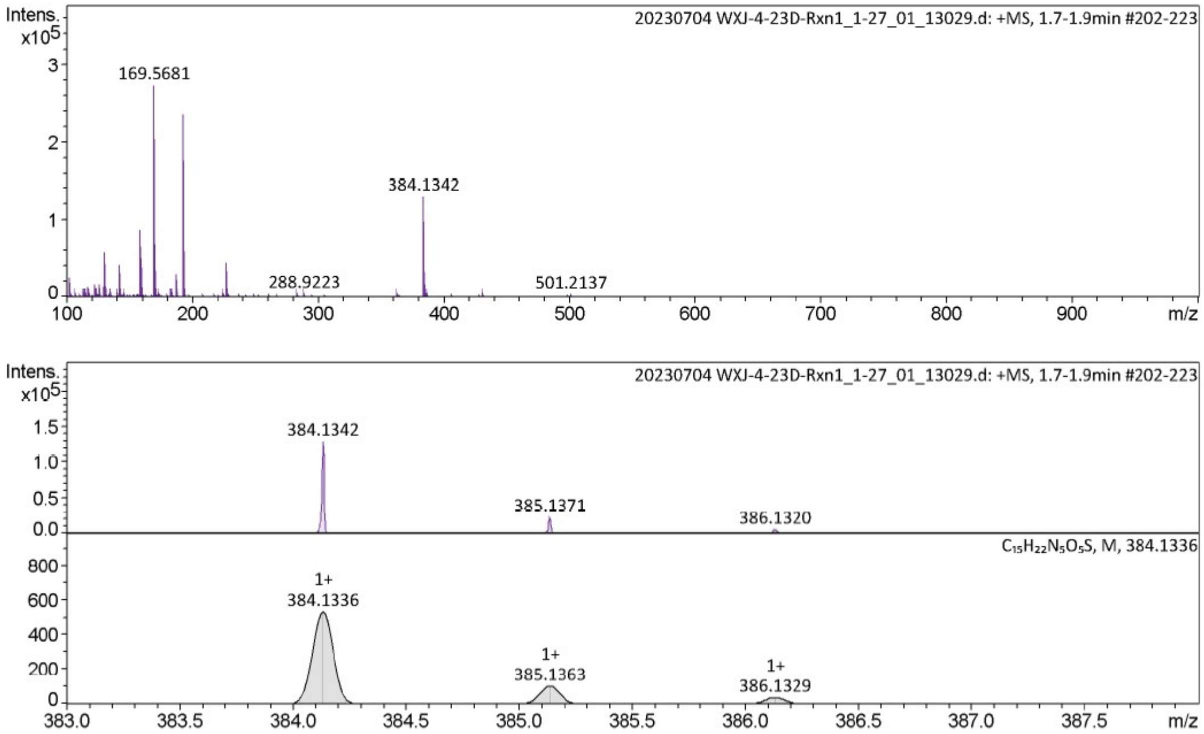

Figure S60. Measured and calculated HR-ESI-MS spectra of S$^{3\text{-deaza}}$AH.

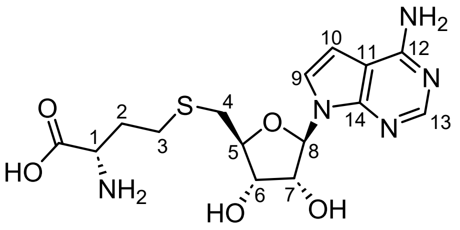

S-tubercidinylhomocysteine (STH)

S-Tubercidinylhomocysteine (STH) from tubercidin.[7] 60 µM *Pa*SAHH was used and the reaction was incubated for 2 days. Unfortunately, the purified sample was not enough to give a clear ^{13}C NMR spectrum, and only ^{1}H NMR and HRMS spectra were analyzed and shown here.

^{1}H NMR (500 MHz, D_2O) δ 8.17 (s, 1H), 7.42 (d, *J* = 3.8 Hz, 1H), 6.71 (d, *J* = 3.8 Hz, 1H), 6.23 (d, *J* = 6.2 Hz, 1H), 4.72 (t, *J* = 5.8 Hz, 1H), 4.38 (dd, *J* = 5.3, 3.8 Hz, 1H), 4.33–4.27 (m, 1H), 3.73–3.67 (m, 1H), 3.03 (dd, *J* = 14.1, 5.2 Hz, 1H), 2.93 (dd, *J* = 14.1, 6.5 Hz, 1H), 2.73–2.63 (m, 2H), 2.15–2.06 (m, 1H), 2.05-1.96 (m, 1H).

HRMS (ESI) Cald. for $C_{15}H_{22}N_5O_5S^+$ (M+H$^+$) 384.1336, found 384.1340.

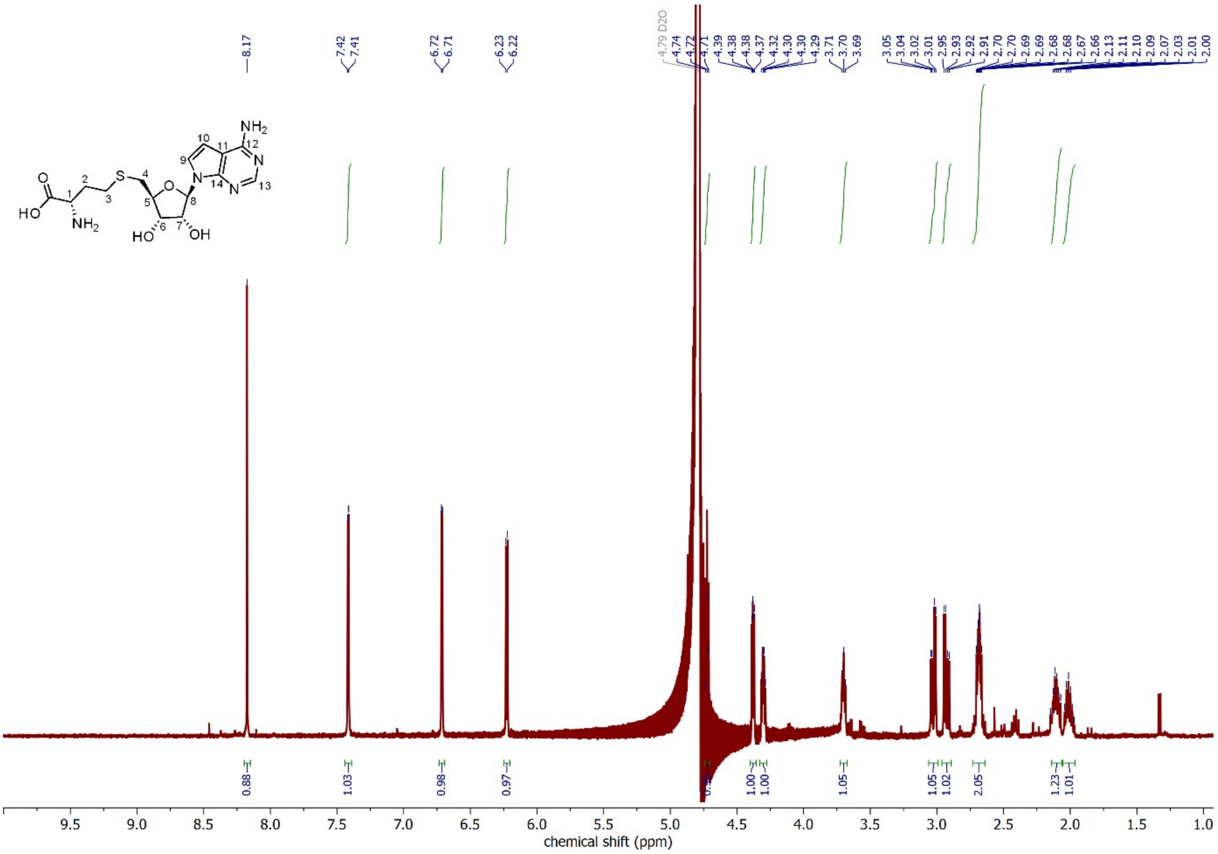

Figure S61. ^{1}H NMR spectrum of STH.

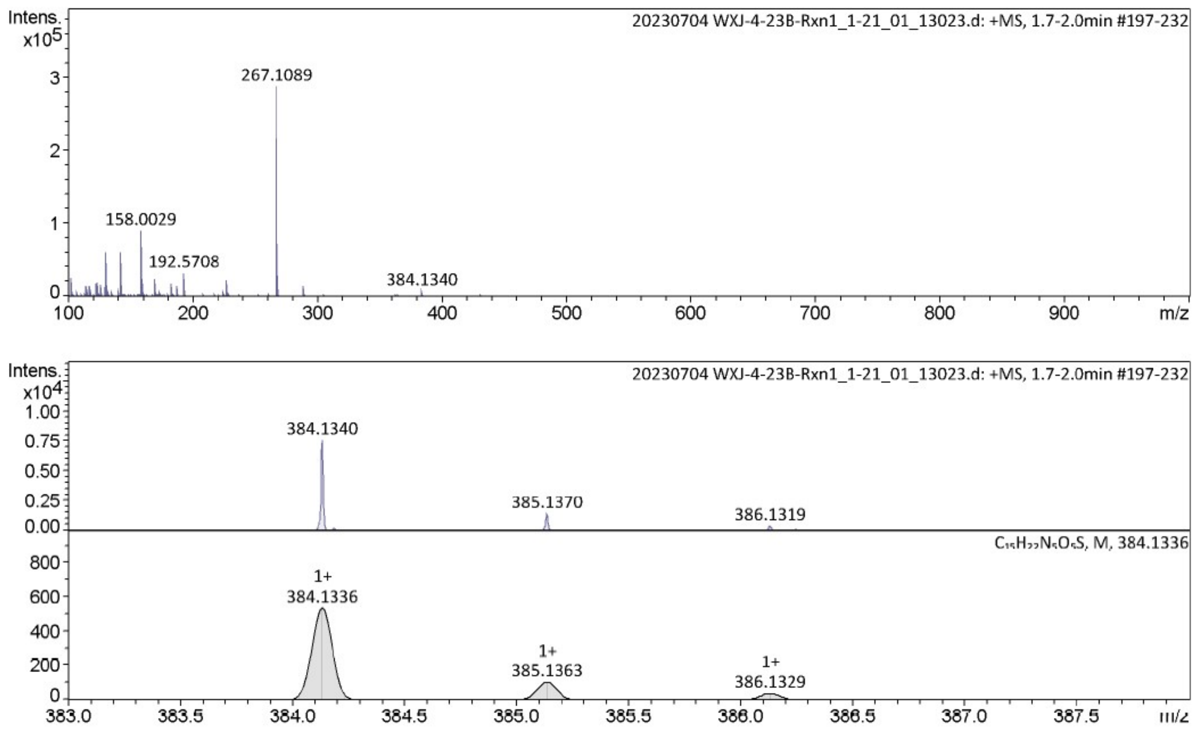

Figure S62. Measured and calculated HR-ESI-MS spectra of STH.

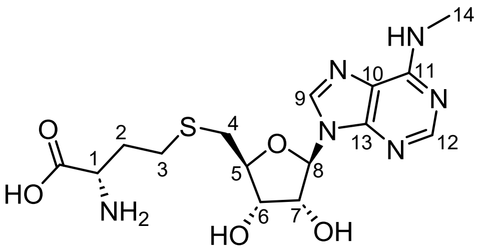

S-N^6-methyladenosylhomocysteine (Sm^{6}AH)

S-*N*^{6}-methyladenosylhomocysteine (Sm^{6}AH) from *N*^{6}-methyladenosine.[9] 20 µM *Mm*SAHH was used.

^{1}H NMR (500 MHz, D_2O) δ 8.49 (s, 1H, H-9), 8.44 (s, 1H, H-12), 6.15 (d, *J* = 4.9 Hz, 1H, H-8), 4.88 (t, *J* = 5.1 Hz, 1H, H-7), 4.46 (t, *J* = 5.1 Hz, 1H, H-5), 4.35 (dt, *J* = 7.1, 4.8 Hz, 1H, H-6), 4.10 (t, *J* = 6.4 Hz, 1H, H-1), 3.23 (s, 3H, H-14), 3.09 (dd, *J* = 14.2, 4.9 Hz, 1H, H-4a), 3.03 (dd, *J* = 14.2, 7.0 Hz, 1H, H-4b), 2.75 (t, *J* = 7.4 Hz, 2H, H-3), 2.30-2.20 (m, 1H, H-2a), 2.19-2.10 (m, 1H, H-2b).

^{13}C NMR (126 MHz, D_2O) δ 172.2 ($\underline{C}O_2H$), 149.4 (C-11, assigned by 2D NMR), 146.7 (C-13), 144.5 (C-12), 142.4 (C-9), 119.4 (C-10, assigned by 2D NMR), 88.4 (C-8), 83.7 (C-6), 73.6 (C-7), 72.3 (C-5), 52.1 (C-1), 33.4 (C-4), 29.8 (C-2), 28.3 (C-14), 27.4 (C-3).

HRMS (ESI) Cald. for $C_{15}H_{23}N_6O_5S^+$ (M+H$^+$) 399.1445, found 399.1447.

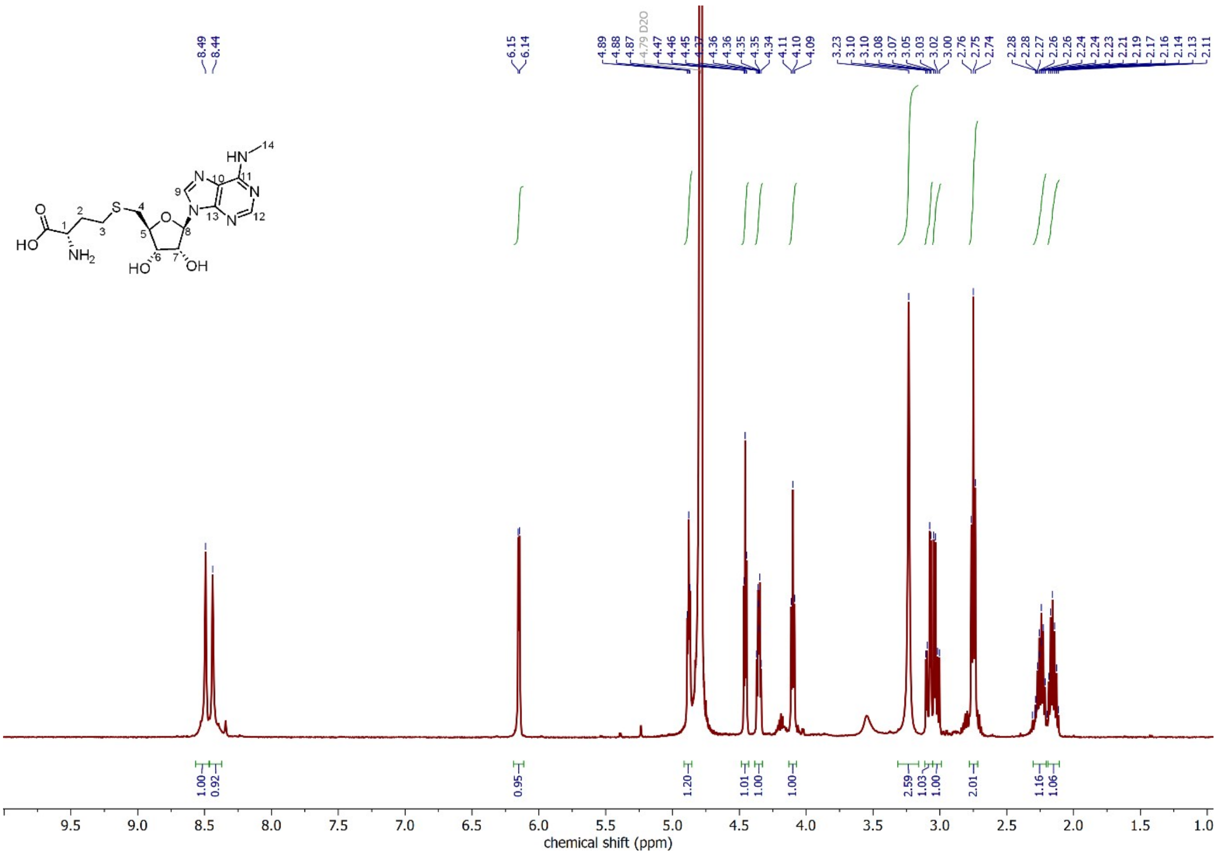

Figure S63. ^{1}H NMR spectrum of Sm^{6}AH.

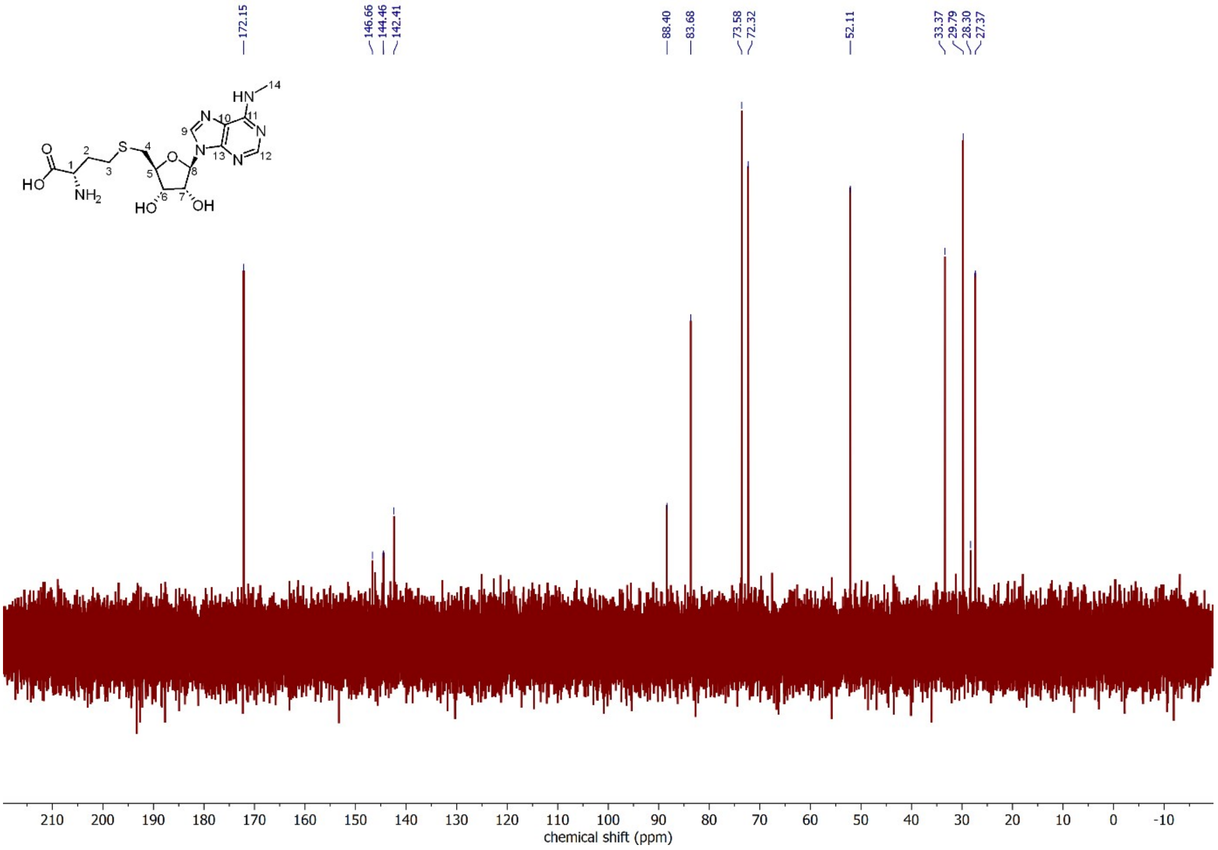

Figure S64. ^{13}C NMR spectrum of Sm[6]AH.

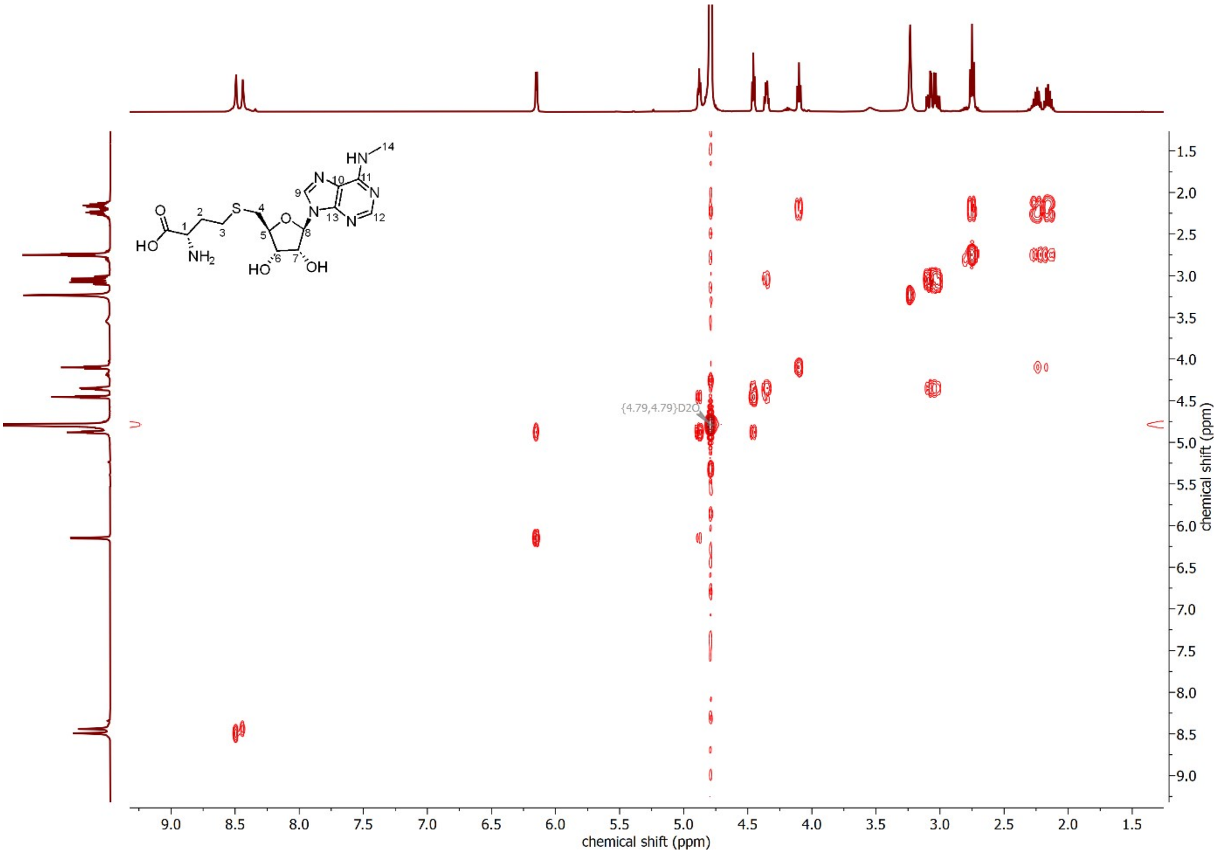

Figure S65. ^{1}H-^{1}H COSY spectrum of Sm[6]AH.

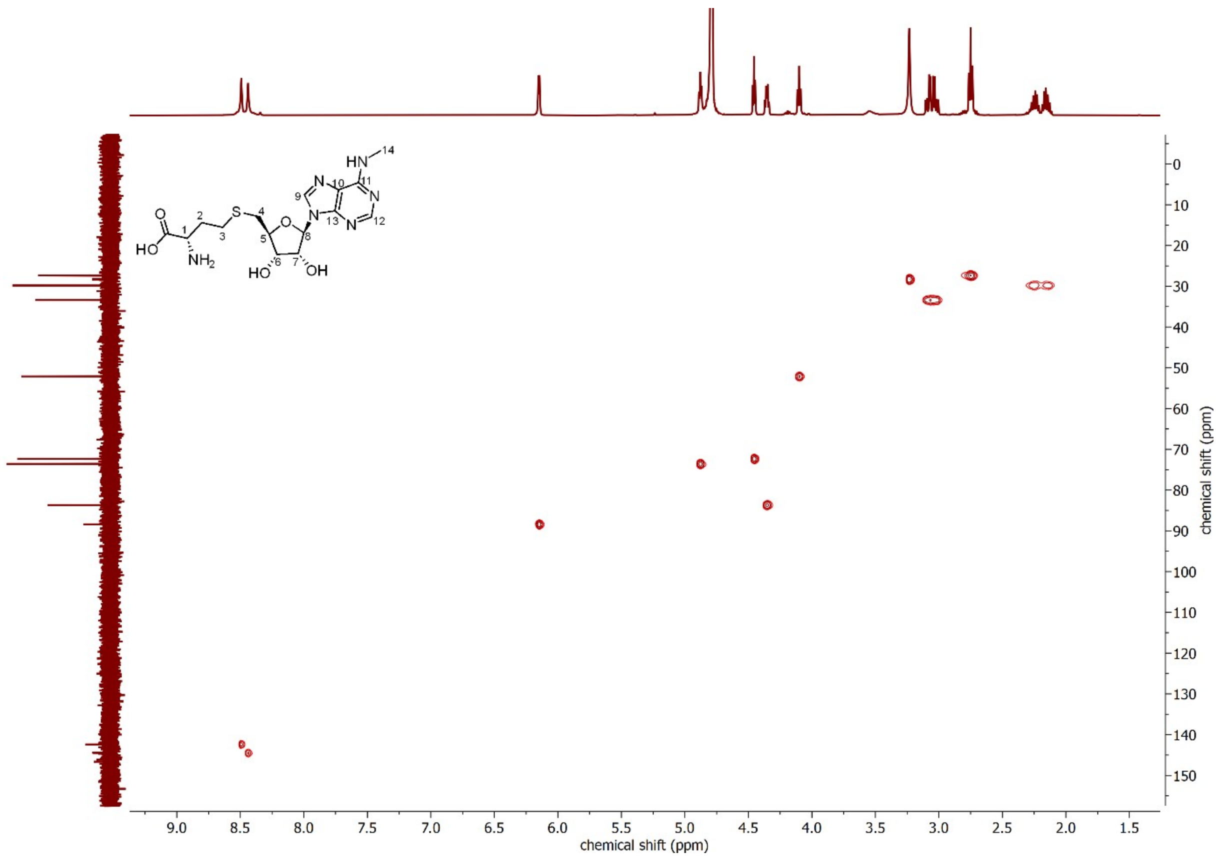

Figure S66. HSQC spectrum of Sm^{6}AH.

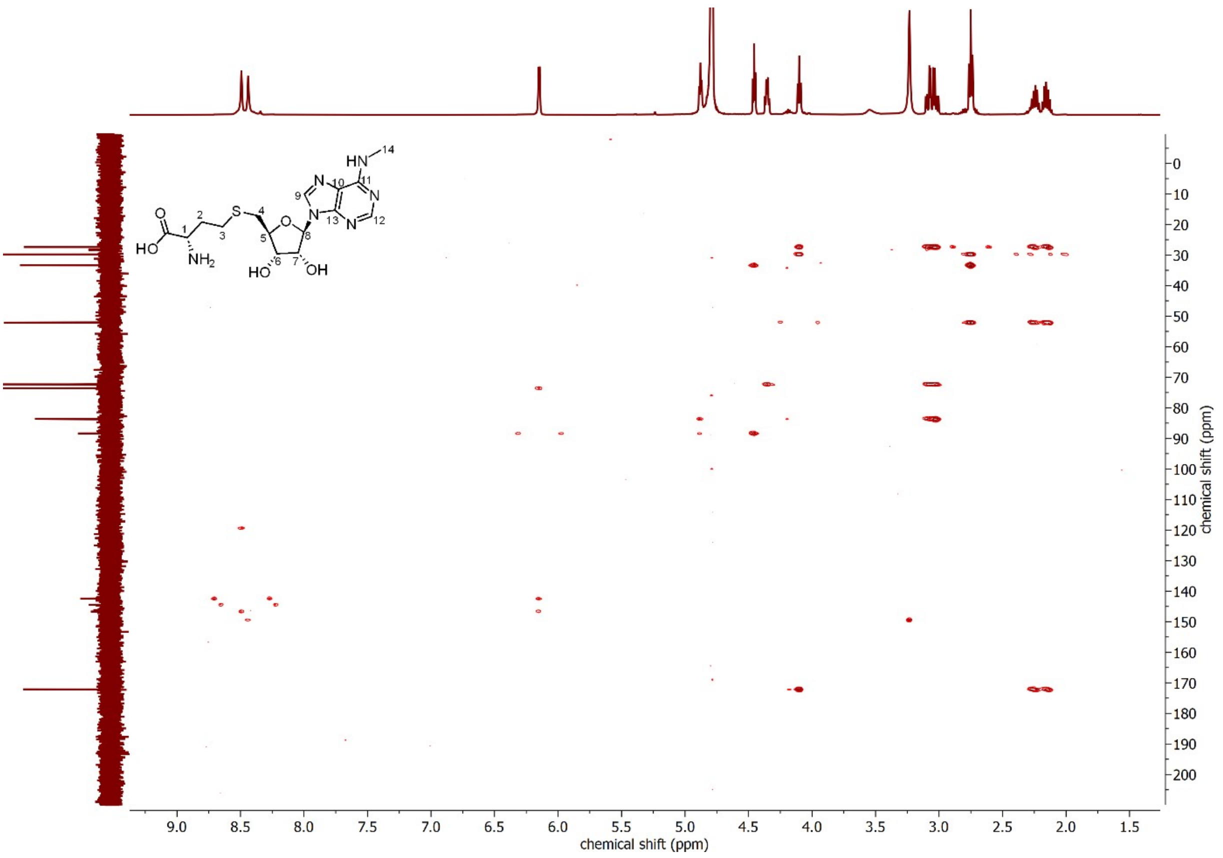

Figure S67. HMBC spectrum of Sm^{6}AH.

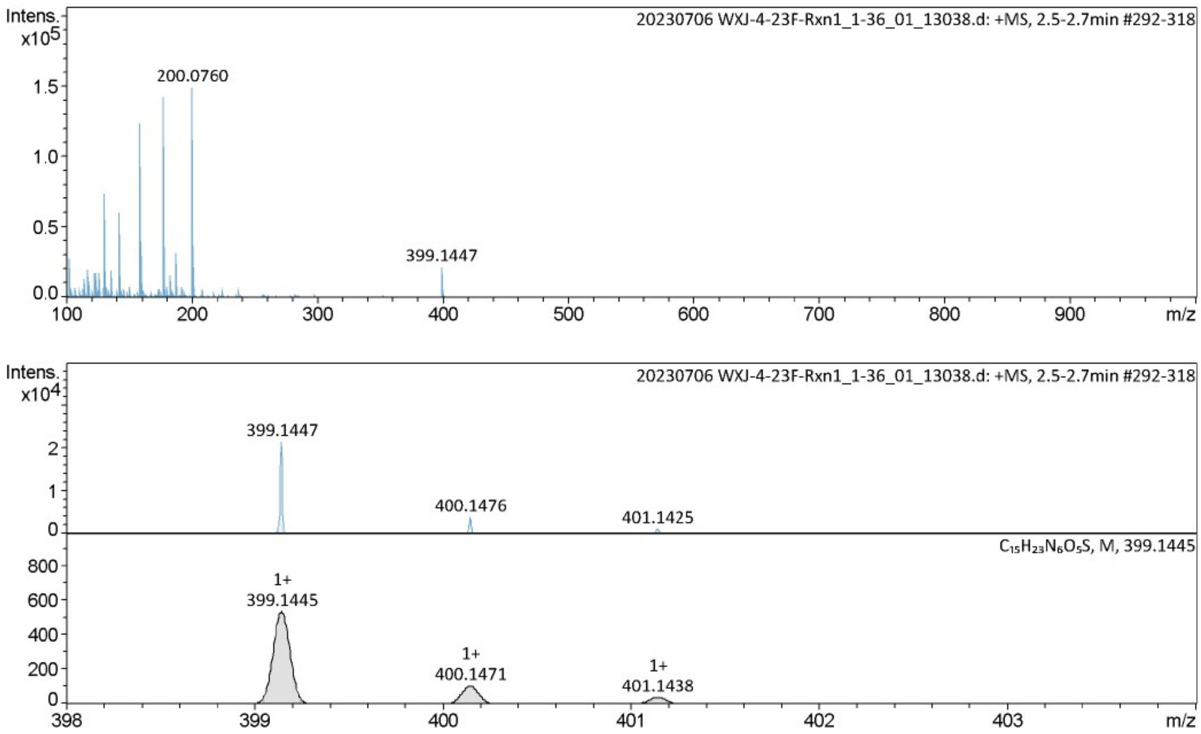

Figure S68. Measured and calculated HR-ESI-MS spectra of Sm^6AH.

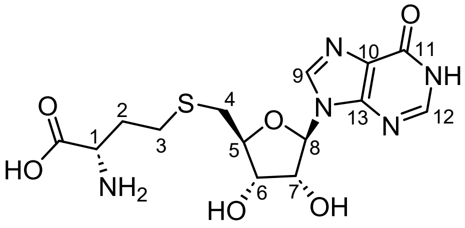

S-inosylhomocysteine (SIH)

S-inosylhomocysteine (SIH) from inosine.[10] 20 µM *Pa*SAHH was used.

^{1}H NMR (500 MHz, D_2O) δ 8.26 (s, 1H, H-9), 8.18 (s, 1H, H-12), 6.07 (d, *J* = 5.2 Hz, 1H, H-8), 4.86 (t, *J* = 5.3 Hz, 1H, H-7), 4.43 (t, *J* = 5.0 Hz, 1H, H-5), 4.33 (dt, *J* = 6.7, 4.9 Hz, 1H, H-6), 3.61 (t, *J* = 6.6 Hz, 1H, H-1), 3.05 (dd, *J* = 14.1, 5.1 Hz, 1H, H-4a), 2.97 (dd, *J* = 14.1, 6.7 Hz, 1H, H-4b), 2.66 (t, *J* = 7.6 Hz, 2H, H-3), 2.06 (dq, *J* = 14.0, 7.3 Hz, 1H, H-2a), 1.95 (dq, *J* = 14.4, 7.3 Hz, 1H, H-2b).

^{13}C NMR (126 MHz, D_2O) δ 177.3 ($\underline{C}O_2H$, assigned by 2D NMR), 163.2 (C-11, assigned by 2D NMR), 150.2 (C-12), 149.3 (C-13), 139.0 (C-9), 123.7 (C-10), 87.5 (C-8), 83.5 (C-6), 73.3 (C-7), 72.3 (C-5), 54.3 (C-1), 33.5 (C-4), 32.0 (C-2), 28.1 (C-3).

HRMS (ESI) Cald. for $C_{15}H_{20}N_5O_6S^+$ ($M+H^+$) 386.1129, found 386.1132.

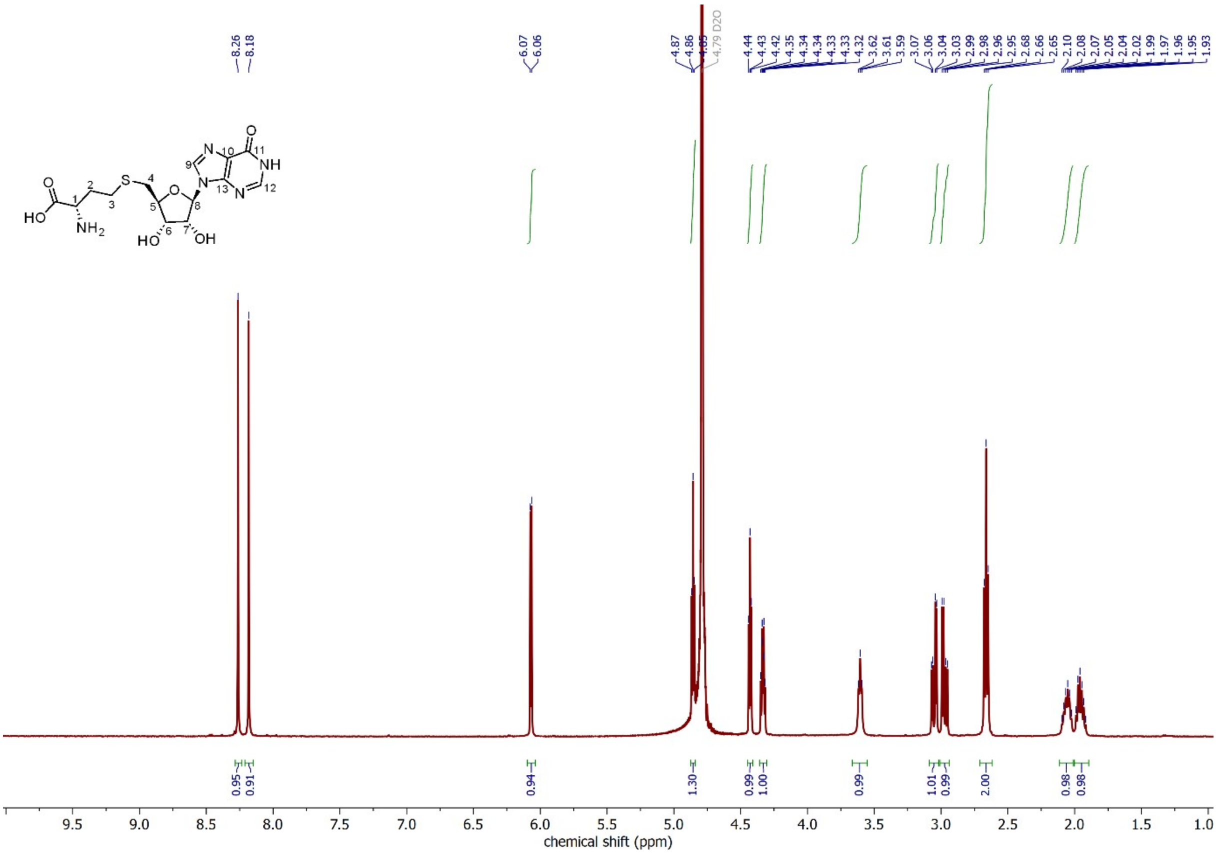

Figure S69. ^{1}H NMR spectrum of SIH.

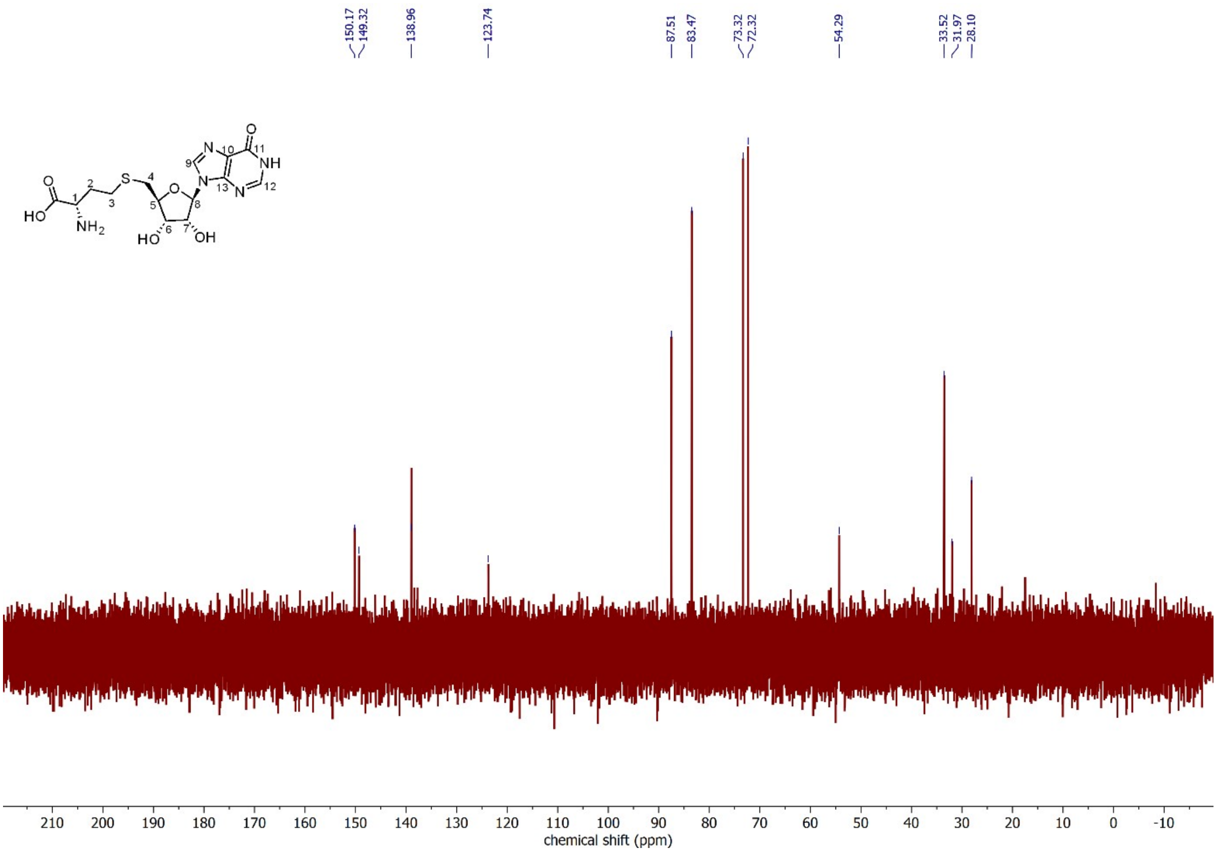

Figure S70. ^{13}C NMR spectrum of SIH.

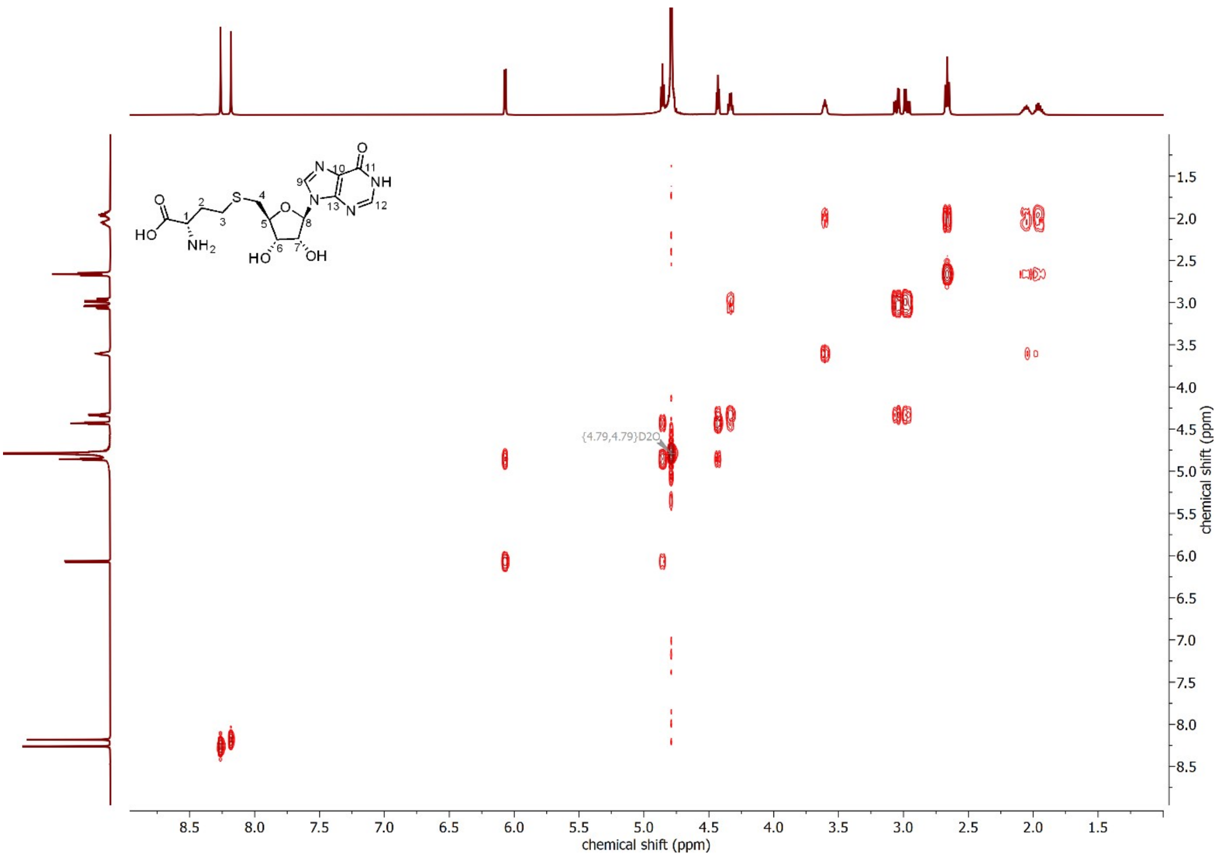

Figure S71. ^{1}H-^{1}H COSY spectrum of SIH.

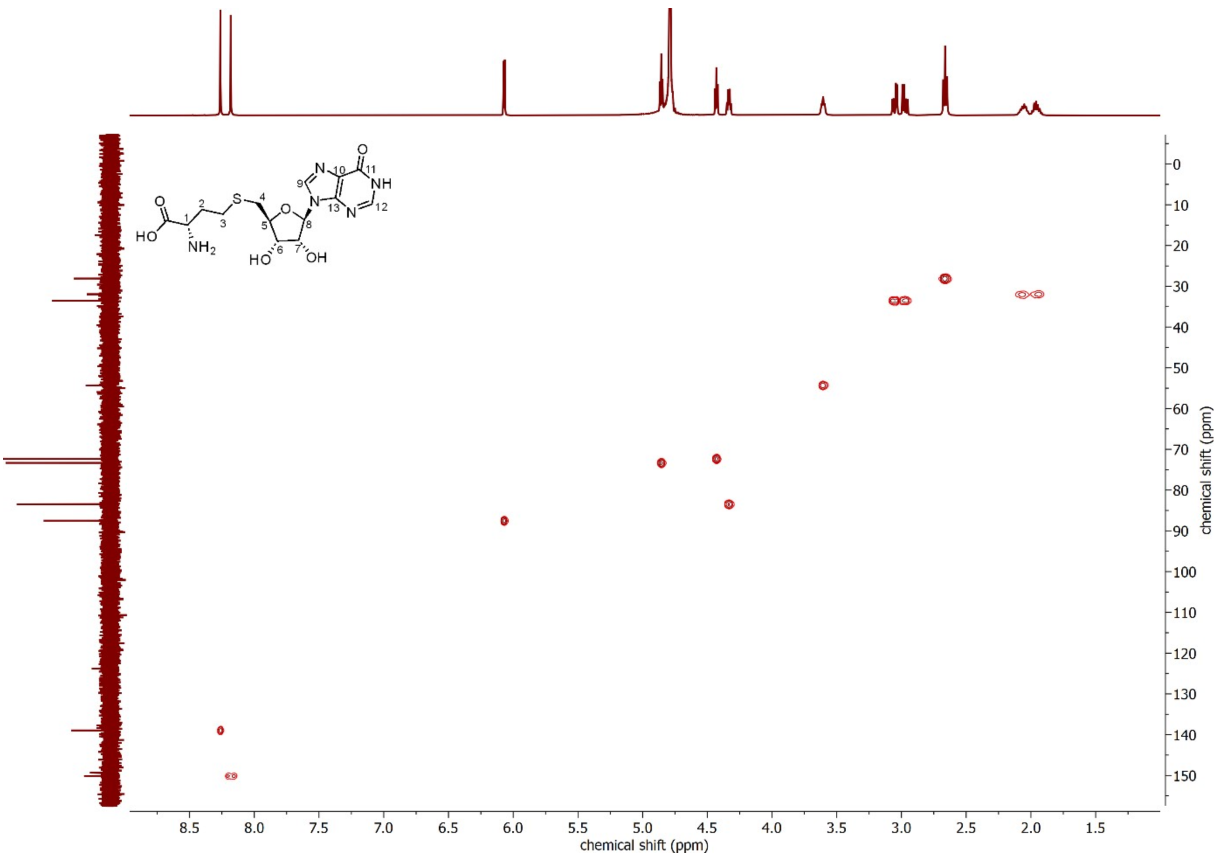

Figure S72. HSQC spectrum of SIH.

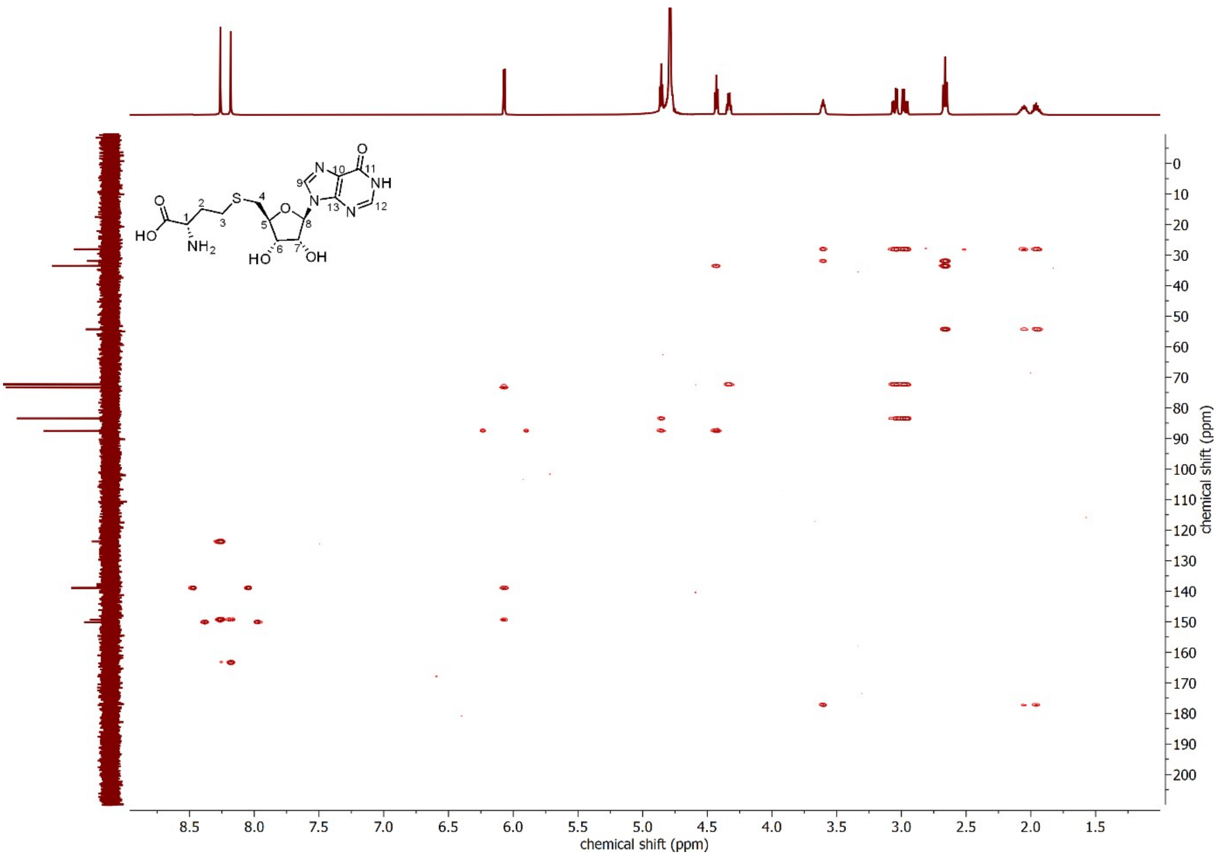

Figure S73. HMBC spectrum of SIH.

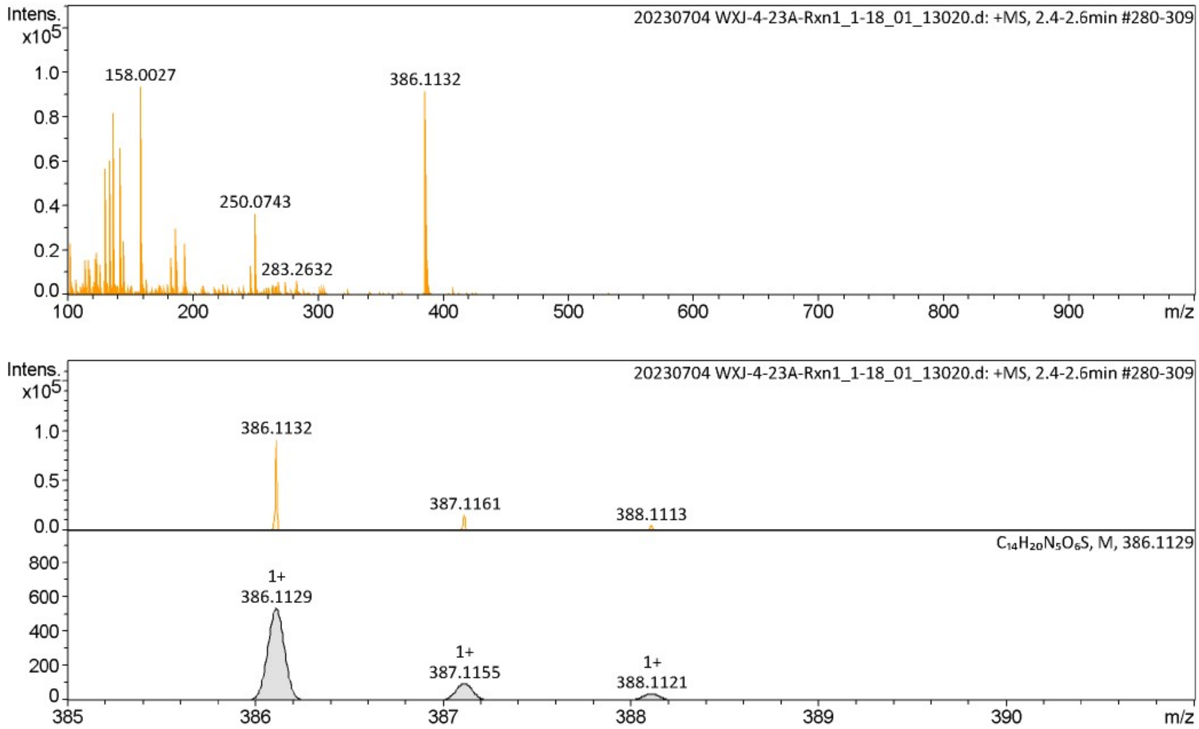

Figure S74. Measured and calculated HR-ESI-MS spectra of SIH.

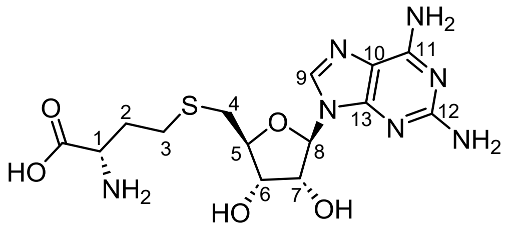

S-2-aminoadenosylhomocysteine ($S^{2\text{-amino}}AH$)

S-2-Aminoadenosylhomocysteine ($S^{2\text{-amino}}AH$) from 2-aminoadenosine.[11] 20 µM *Pa*SAHH was used.

^1H NMR (500 MHz, D_2O) δ 8.02 (s, 1H, H-9), 5.91 (d, *J* = 5.2 Hz, 1H, H-8), 4.83 (t, *J* = 5.3 Hz, 1H, H-7), 4.41 (t, *J* = 4.9 Hz, 1H, H-5), 4.30 (dt, *J* = 6.6, 4.8 Hz, 1H, H-6), 3.66□3.53 (m, 1H, H-1), 3.03 (dd, *J* = 14.2, 5.0 Hz, 1H, H-4a), 2.95 (dd, *J* = 14.2, 6.6 Hz, 1H, H-4b), 2.65 (dd, *J* = 8.1, 7.1 Hz, 2H, H-3), 2.04 (dq, *J* = 13.8, 7.2 Hz, 1H, H-2a), 1.93 (dq, *J* = 14.5, 7.3 Hz, 1H, H-2b).

^{13}C NMR (126 MHz, D_2O) δ 177.4 (<u>C</u>O_2H, assigned by 2D NMR), 160.2 (C-12), 156.1 (C-11), 151.2 (C-13), 137.5 (C-9), 113.2 (C-10), 86.9 (C-8), 83.3 (C-6), 73.0 (C-7), 72.4 (C-5), 54.3 (C-1), 33.5 (C-4), 32.0 (C-2), 28.2 (C-3).

HRMS (ESI) Cald. for $C_{14}H_{22}N_7O_5S^+$ ($M+H^+$) 400.1398, found 400.1405.

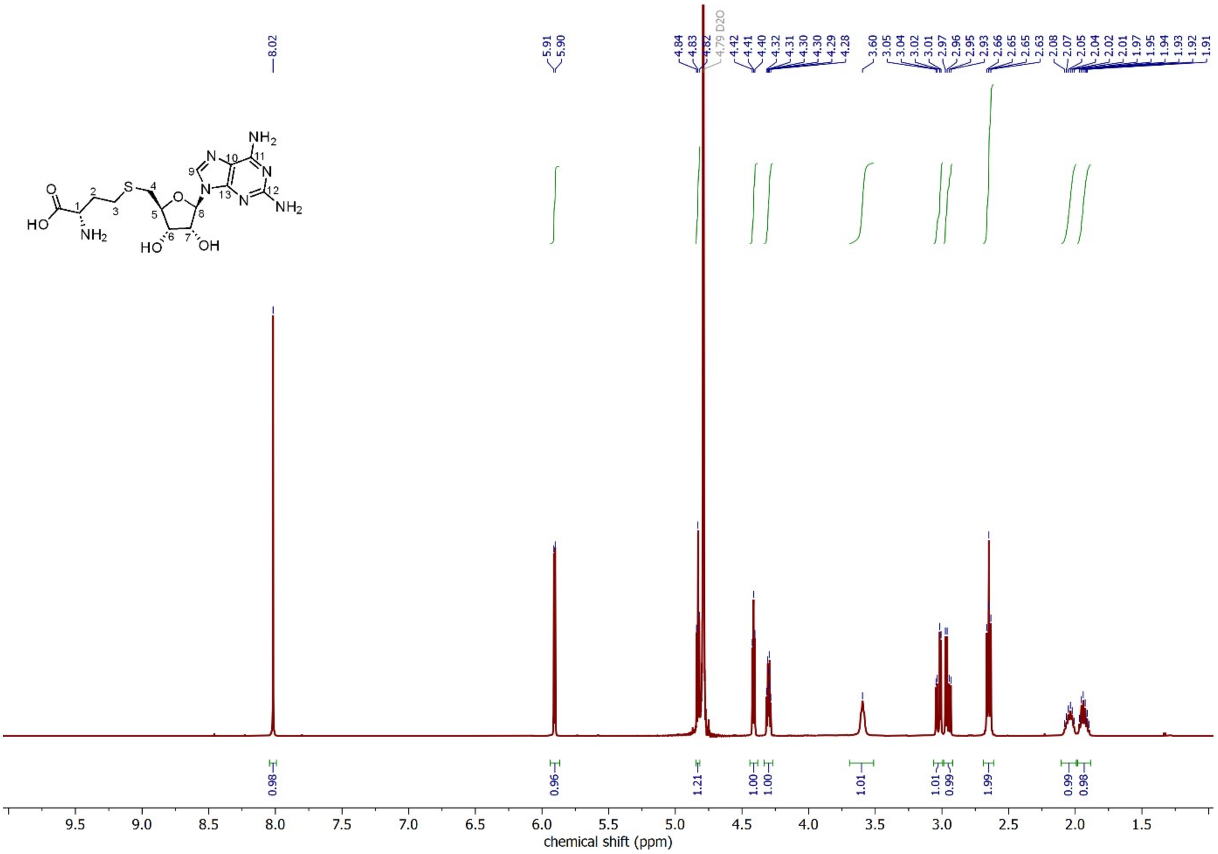

Figure S75. ^1H NMR spectrum of $S^{2\text{-amino}}AH$.

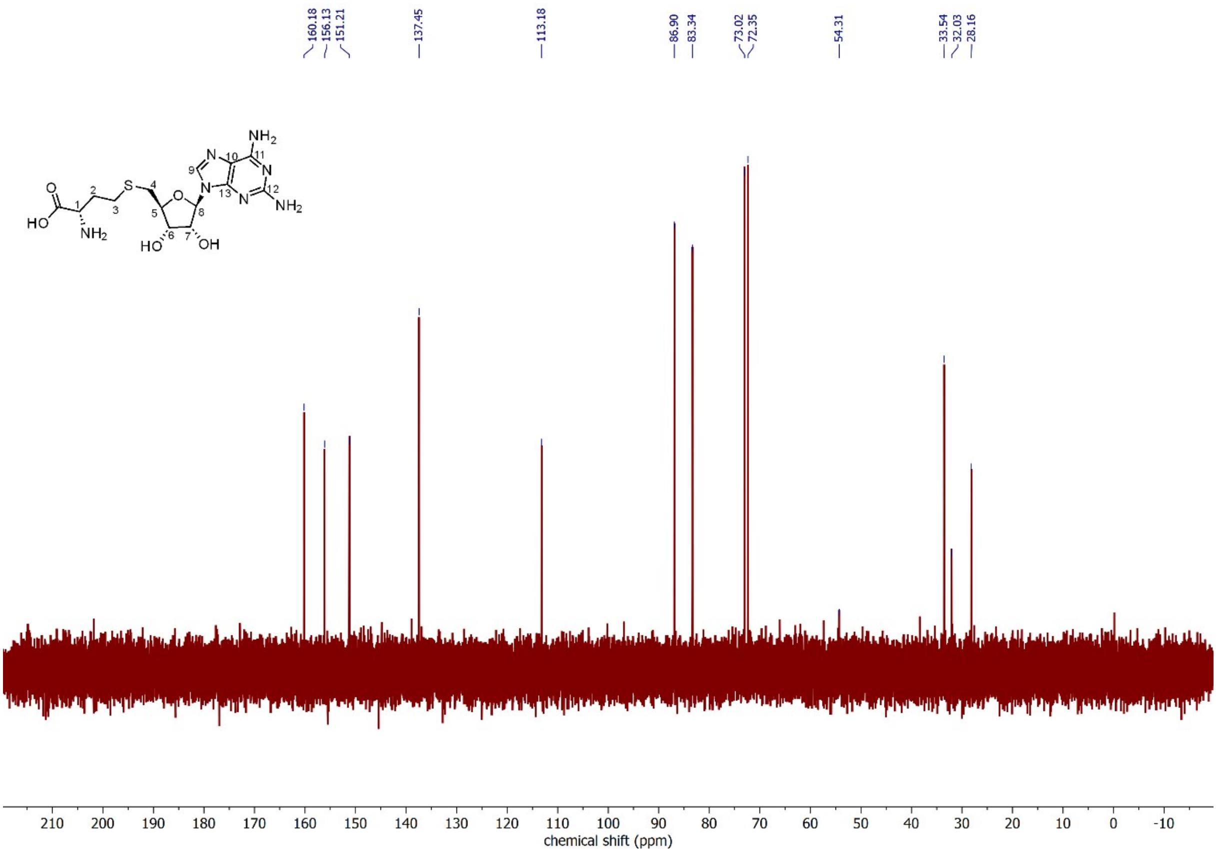

Figure S76. ^{13}C NMR spectrum of S$^{2\text{-amino}}$AH.

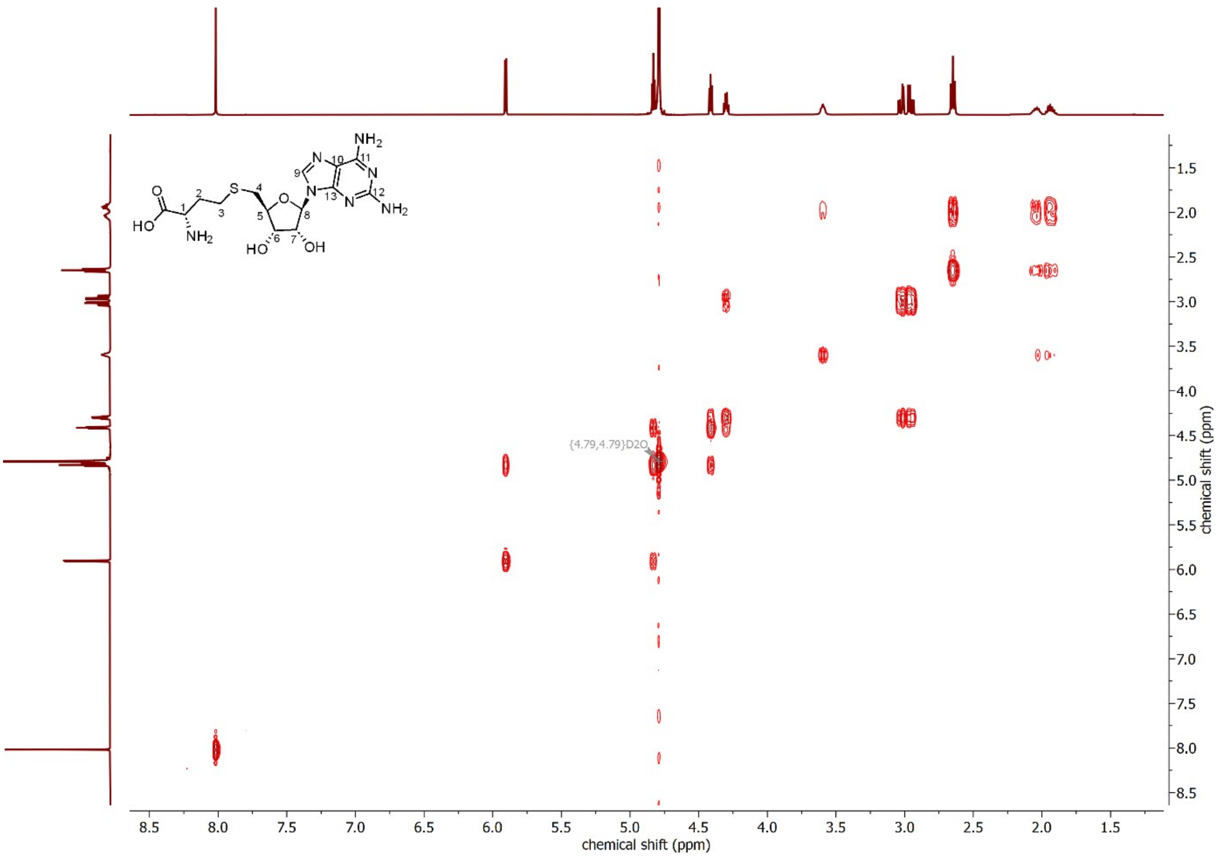

Figure S77. ^{1}H-^{1}H COSY spectrum of S$^{2\text{-amino}}$AH.

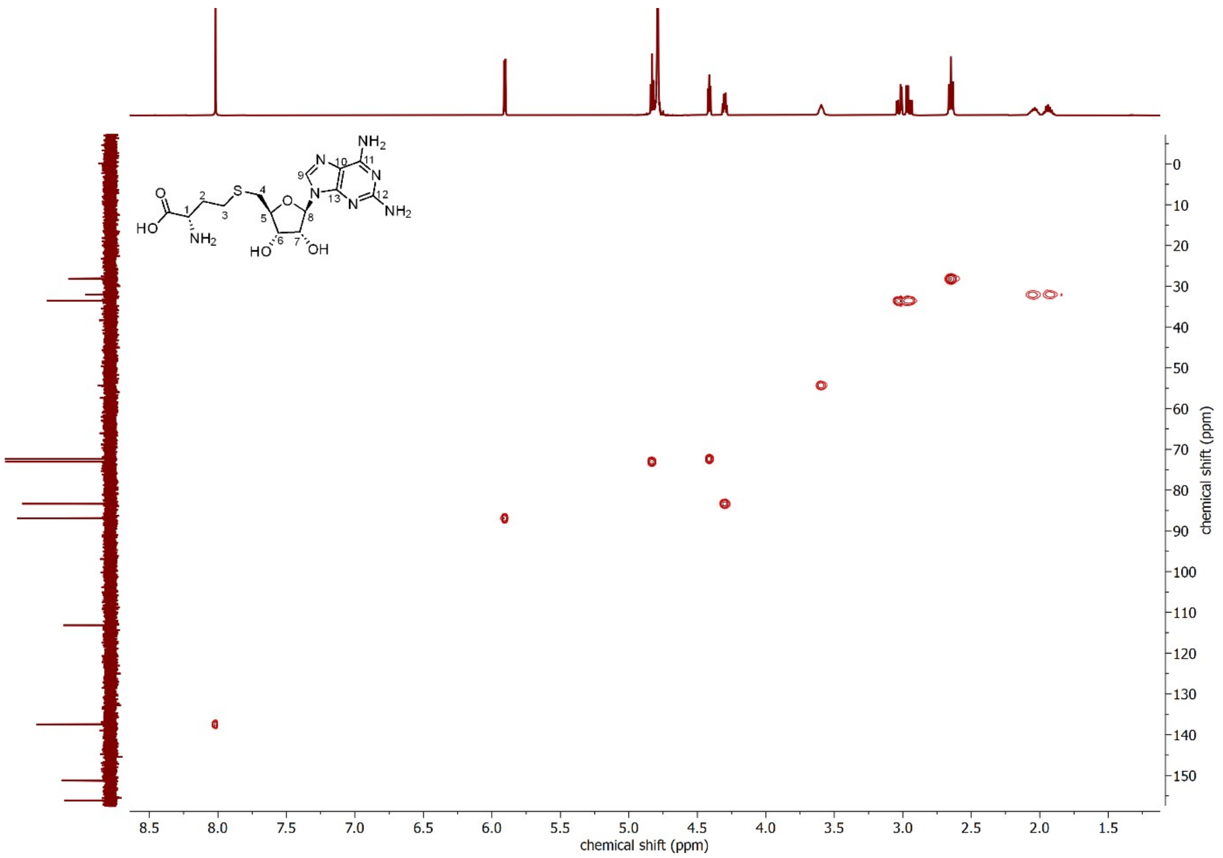

Figure S78. HSQC spectrum of S$^{2\text{-amino}}$AH.

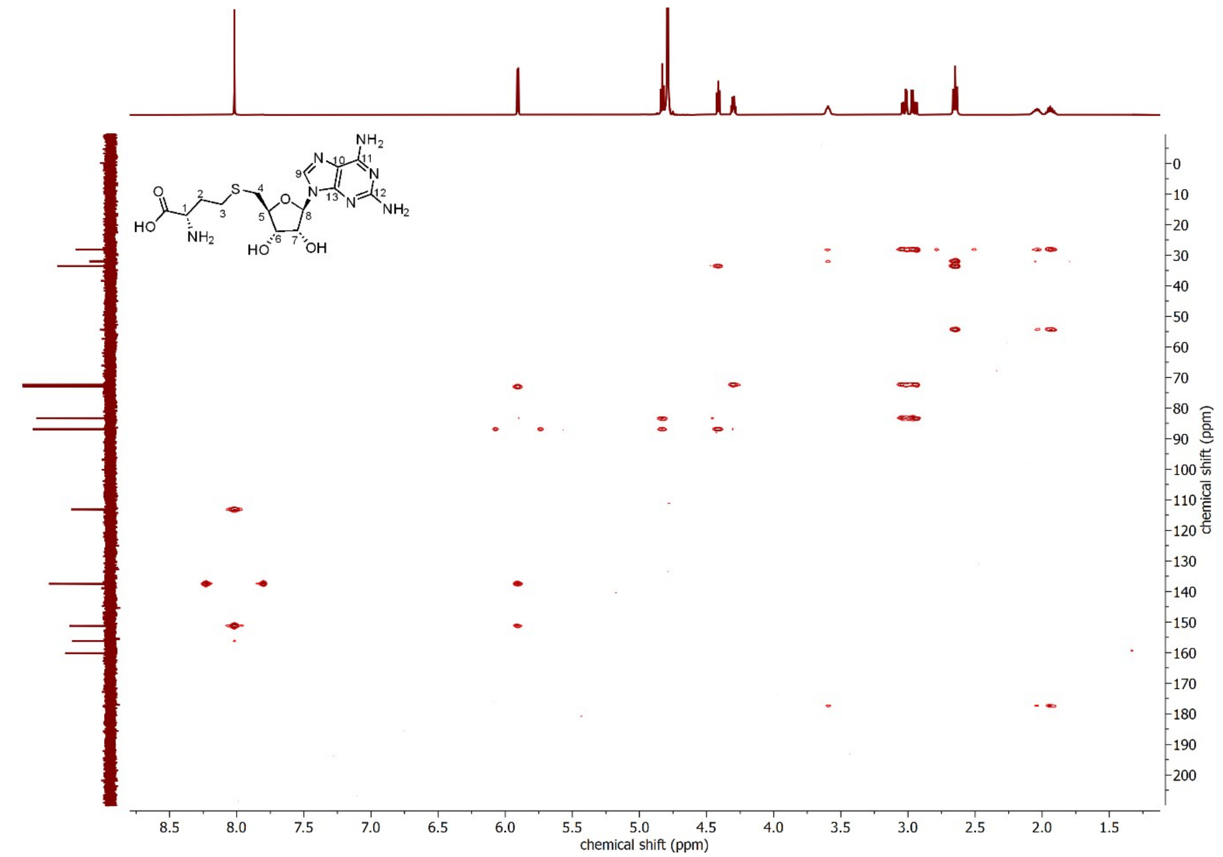

Figure S79. HMBC spectrum of S$^{2\text{-amino}}$AH.

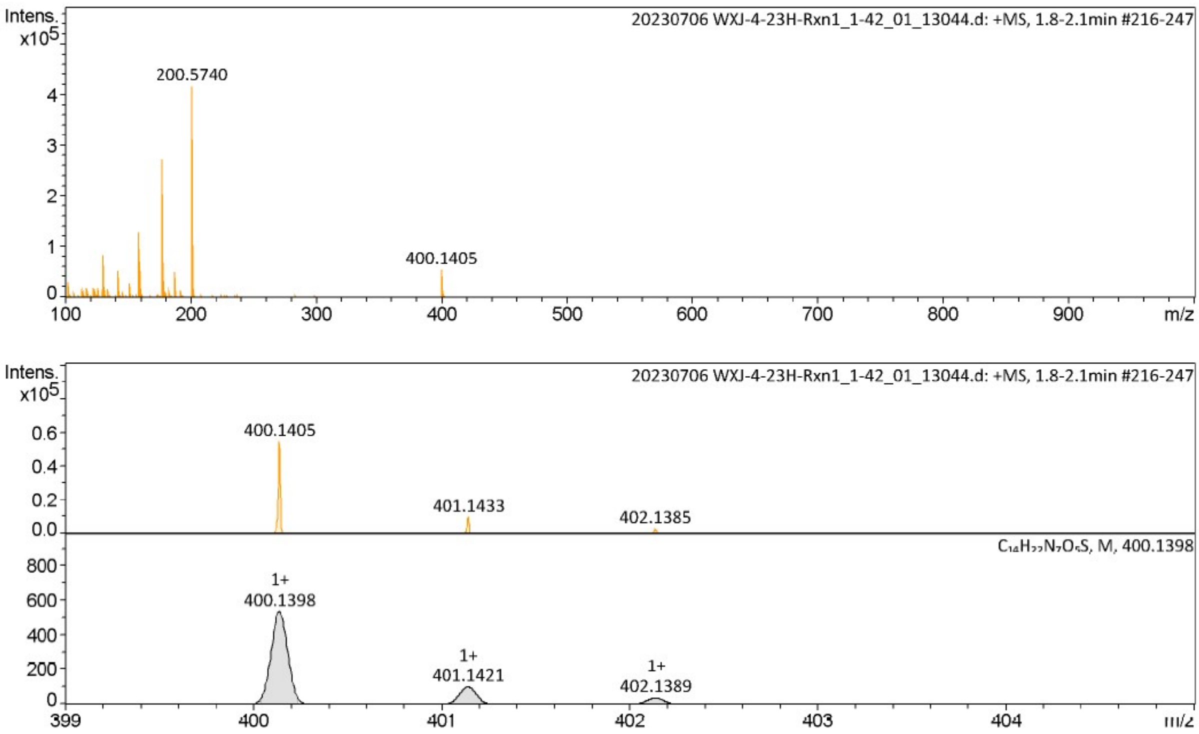

Figure S80. Measured and calculated HR-ESI-MS spectra of S$^{2\text{-amino}}$AH.

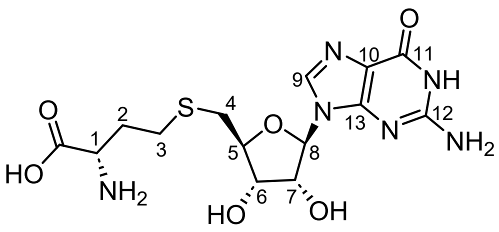

S-guanosylhomocysteine (SGH)

S-Guanosylhomocysteine (SGH) from guanosine.[12] 60 μM *Mm*SAHH was used and the reaction was incubated for 2 days. Unfortunately, the yield of the target product SGH was quite low and most of the substrate guanosine remained. The pure SGH could not be isolated from the reaction mixture by preparative reverse-phase HPLC. Thus, the clean NMR spectra of SGH were not obtained. The reaction mixture was analyzed by HRMS and the molecular weight of SGH was detected.

HRMS (ESI) Cald. for $C_{14}H_{21}N_6O_6S^+$ (M+H$^+$) 401.1238, found 401.1241.

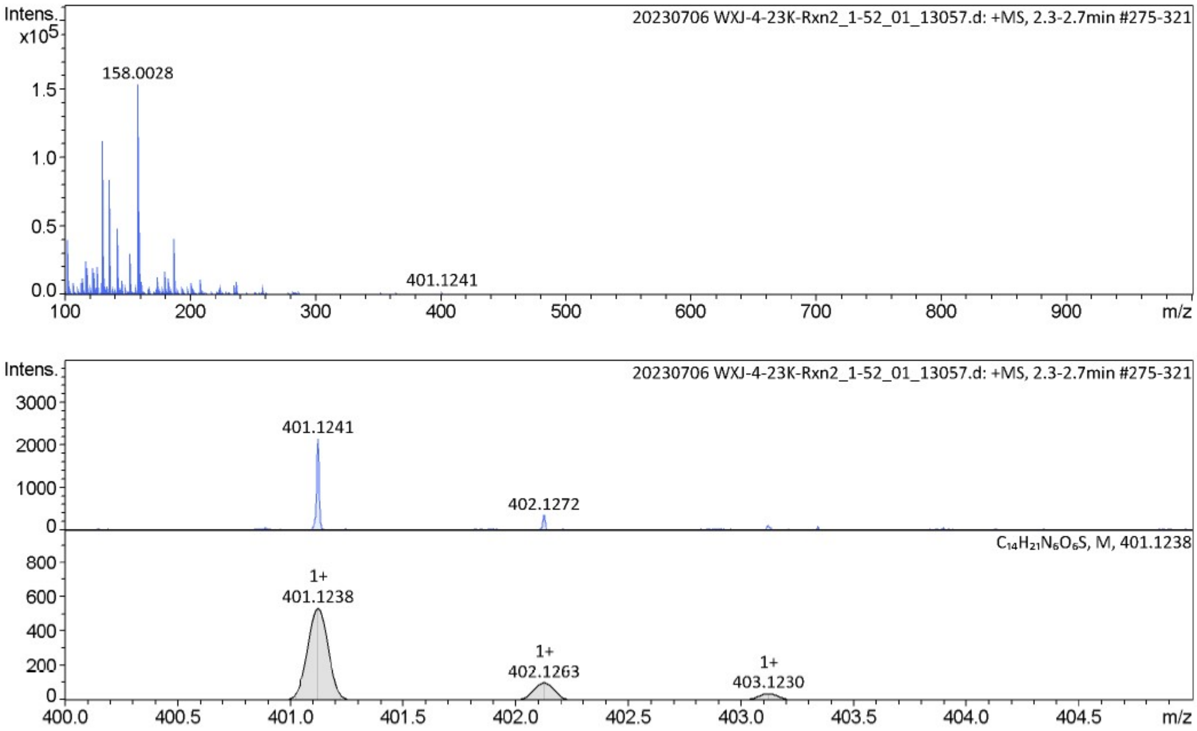

Figure S81. Measured and calculated HR-ESI-MS spectra of SGH.

Measuring observed rate constants of SAH analog production by three-enzyme cascade

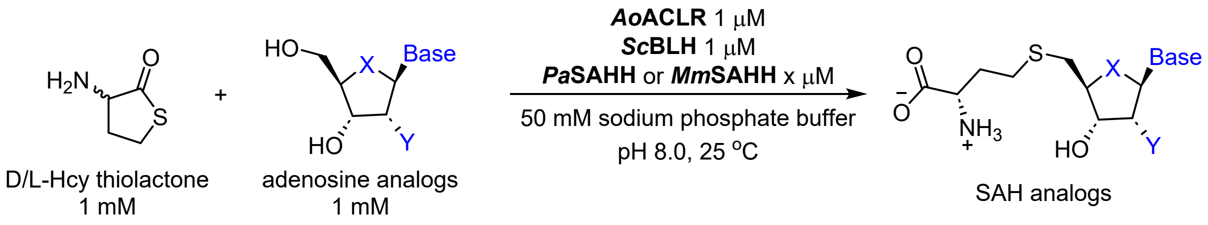

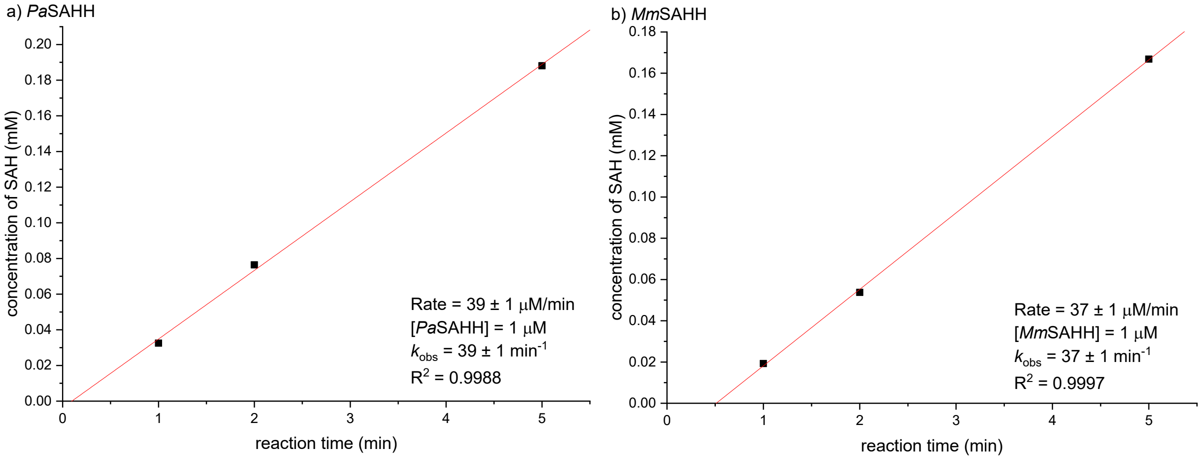

Figure S82. The observed rate constant (k_{obs}) of SAH production by ACLR-BLH-SAHH enzyme cascade. The concentration of SAH was determined by HPLC. k_{obs} is equal to the initial rate of SAH formation divided by the concentration of SAHH. a) with *Pa*SAHH; b) with *Mm*SAHH. Reaction conditions: 200 µL reaction containing 1 mM D/L-Hcy thiolactone, 1 mM adenosine, 1 µM *Ao*ACLR, 1 µM *Sc*BLH, 1 µM *Pa*SAHH or *Mm*SAHH in 50 mM sodium phosphate buffer (pH 8.0) at 25 °C.

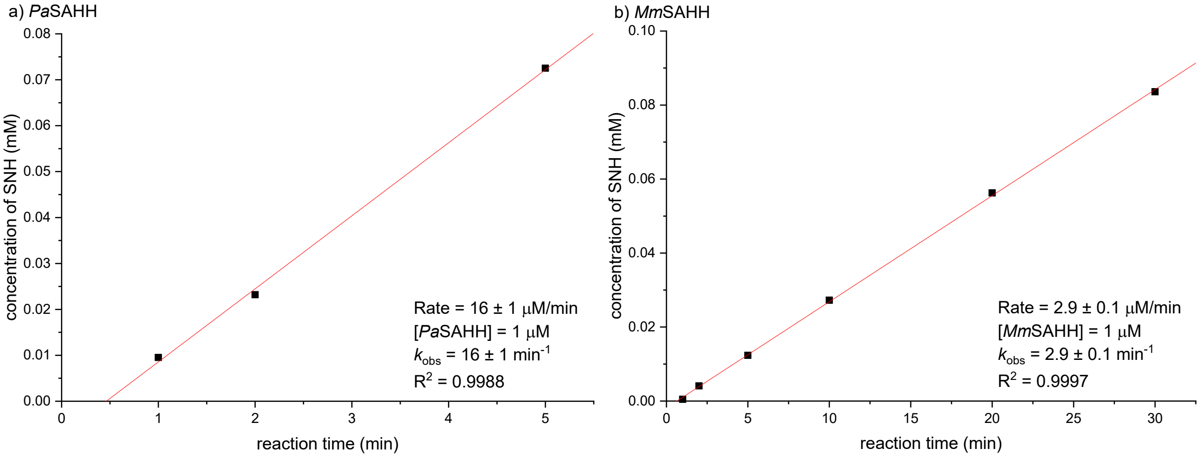

Figure S83. The observed rate constant (k_{obs}) of SNH production by ACLR-BLH-SAHH enzyme cascade. The concentration of SNH was determined by HPLC. k_{obs} is equal to the initial rate of SNH formation divided by the concentration of SAHH. a) with *Pa*SAHH; b) with *Mm*SAHH. Reaction conditions: 200 µL reaction containing 1 mM D/L-Hcy thiolactone, 1 mM nebularine, 1 µM *Ao*ACLR, 1 µM *Sc*BLH, 1 µM *Pa*SAHH or *Mm*SAHH in 50 mM sodium phosphate buffer (pH 8.0) at 25 °C.

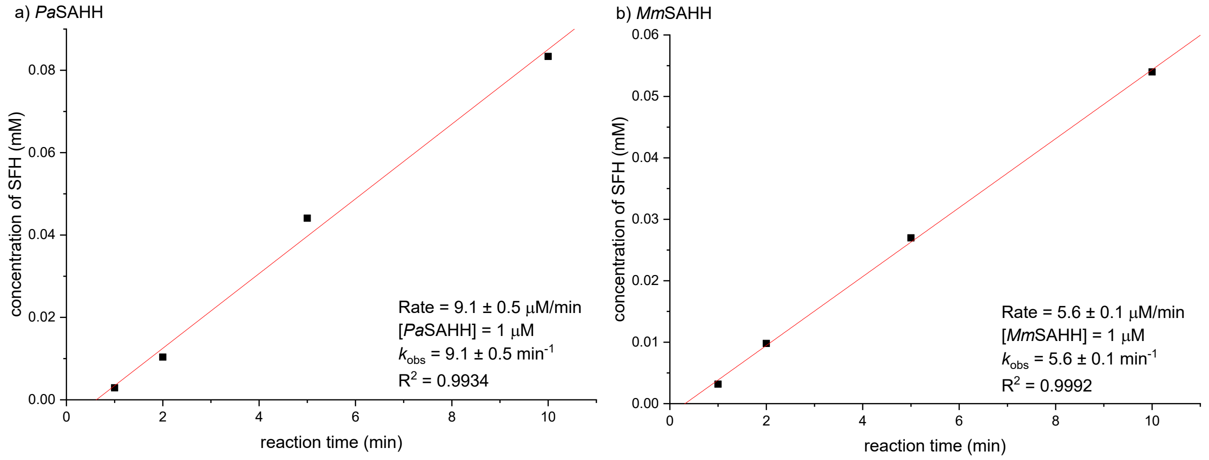

Figure S84. The observed rate constant (k_{obs}) of SFH production by ACLR-BLH-SAHH enzyme cascade. The concentration of SFH was determined by HPLC. k_{obs} is equal to the initial rate of SFH formation divided by the concentration of SAHH. a) with *Pa*SAHH; b) with *Mm*SAHH. Reaction conditions: 200 µL reaction containing 1 mM D/L-Hcy thiolactone, 1 mM formycin A, 1 µM *Ao*ACLR, 1 µM *Sc*BLH, 1 µM *Pa*SAHH or *Mm*SAHH in 50 mM sodium phosphate buffer (pH 8.0) at 25 °C.

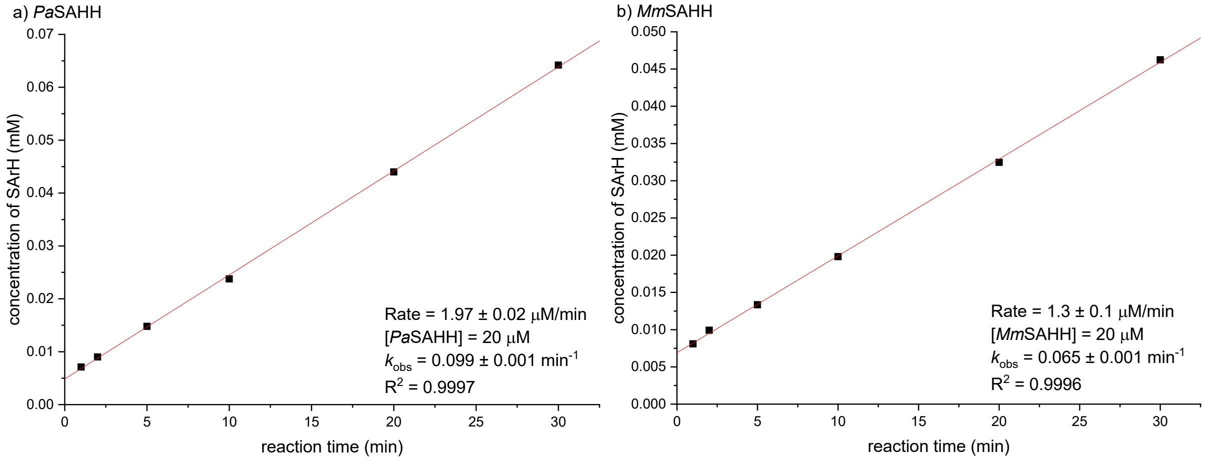

Figure S85. The observed rate constant (k_{obs}) of SArH production by ACLR-BLH-SAHH enzyme cascade. The concentration of SArH was determined by HPLC. k_{obs} is equal to the initial rate of SArH formation divided by the concentration of SAHH. a) with *Pa*SAHH; b) with *Mm*SAHH. Reaction conditions: 200 µL reaction containing 1 mM D/L-Hcy thiolactone, 1 mM aristeromycin, 1 µM *Ao*ACLR, 1 µM *Sc*BLH, 20 µM *Pa*SAHH or *Mm*SAHH in 50 mM sodium phosphate buffer (pH 8.0) at 25 °C.

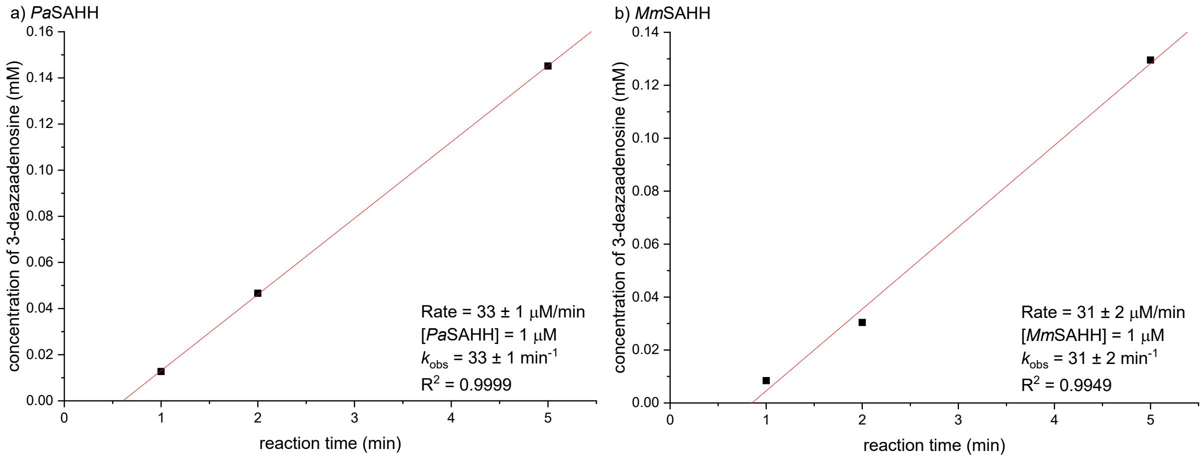

Figure S86. The observed rate constant (k_{obs}) of S$^{3\text{-deaza}}$AH production by ACLR-BLH-SAHH enzyme cascade. The concentration of S$^{3\text{-deaza}}$AH was determined by HPLC. k_{obs} is equal to the initial rate of S$^{3\text{-deaza}}$AH formation divided by the concentration of SAHH. a) with *Pa*SAHH; b) with *Mm*SAHH. Reaction conditions: 200 µL reaction containing 1 mM D/L-Hcy thiolactone, 1 mM 3-deazaadenosine, 1 µM *Ao*ACLR, 1 µM *Sc*BLH, 1 µM *Pa*SAHH or *Mm*SAHH in 50 mM sodium phosphate buffer (pH 8.0) at 25 °C.

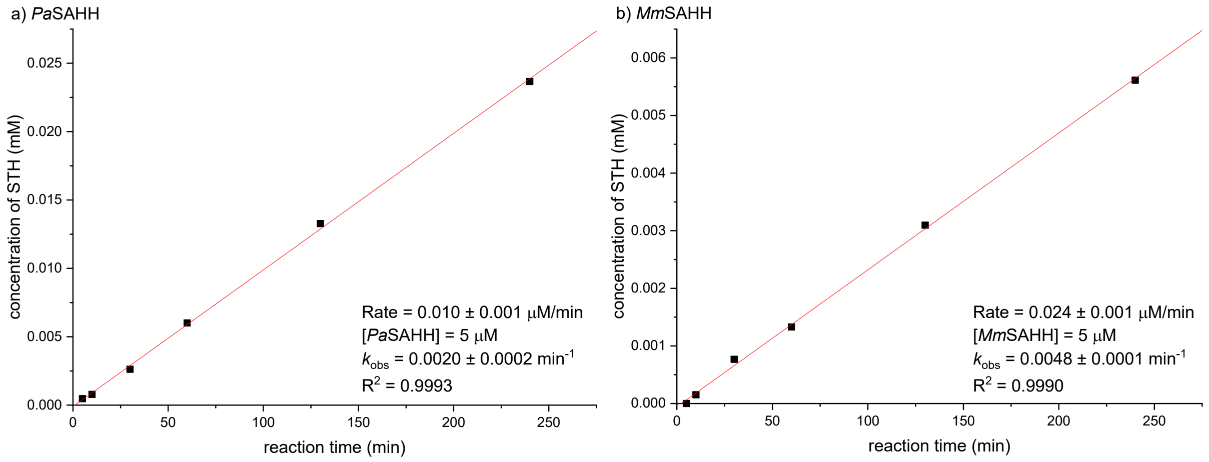

Figure S87. The observed rate constant (k_{obs}) of STH production by ACLR-BLH-SAHH enzyme cascade. The concentration of STH was determined by HPLC. k_{obs} is equal to the initial rate of STH formation divided by the concentration of SAHH. a) with *Pa*SAHH; b) with *Mm*SAHH. Reaction conditions: 200 µL reaction containing 1 mM D/L-Hcy thiolactone, 1 mM tubercidin, 1 µM *Ao*ACLR, 1 µM *Sc*BLH, 5 µM *Pa*SAHH or *Mm*SAHH in 50 mM sodium phosphate buffer (pH 8.0) at 25 °C.

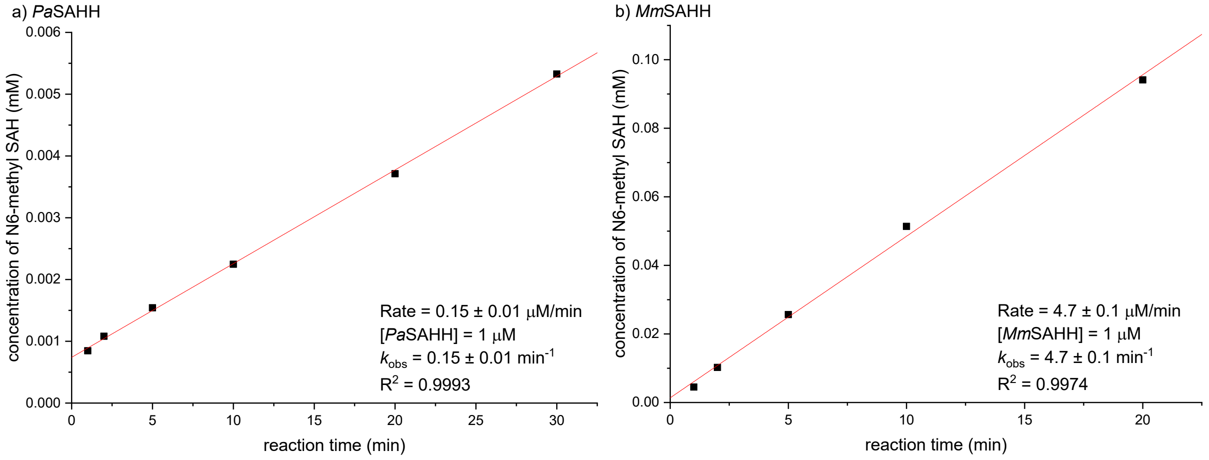

Figure S88. The observed rate constant (k_{obs}) of Sm^6AH production by ACLR-BLH-SAHH enzyme cascade. The concentration of Sm^6AH was determined by HPLC. k_{obs} is equal to the initial rate of Sm^6AH formation divided by the concentration of SAHH. a) with *Pa*SAHH; b) with *Mm*SAHH. Reaction conditions: 200 μL reaction containing 1 mM D/L-Hcy thiolactone, 1 mM N^6-methyladenosine, 1 μM *Ao*ACLR, 1 μM *Sc*BLH, 1 μM *Pa*SAHH or *Mm*SAHH in 50 mM sodium phosphate buffer (pH 8.0) at 25 °C.

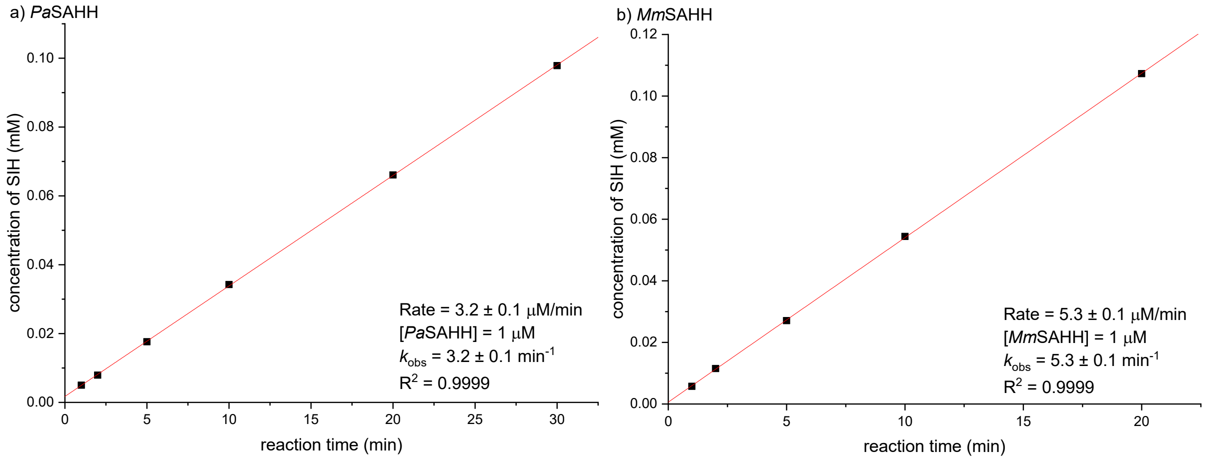

Figure S89. The observed rate constant (k_{obs}) of SIH production by ACLR-BLH-SAHH enzyme cascade. The concentration of SIH was determined by HPLC. k_{obs} is equal to the initial rate of SIH formation divided by the concentration of SAHH. a) with *Pa*SAHH; b) with *Mm*SAHH. Reaction conditions: 200 μL reaction containing 1 mM D/L-Hcy thiolactone, 1 mM inosine, 1 μM *Ao*ACLR, 1 μM *Sc*BLH, 1 μM *Pa*SAHH or *Mm*SAHH in 50 mM sodium phosphate buffer (pH 8.0) at 25 °C.

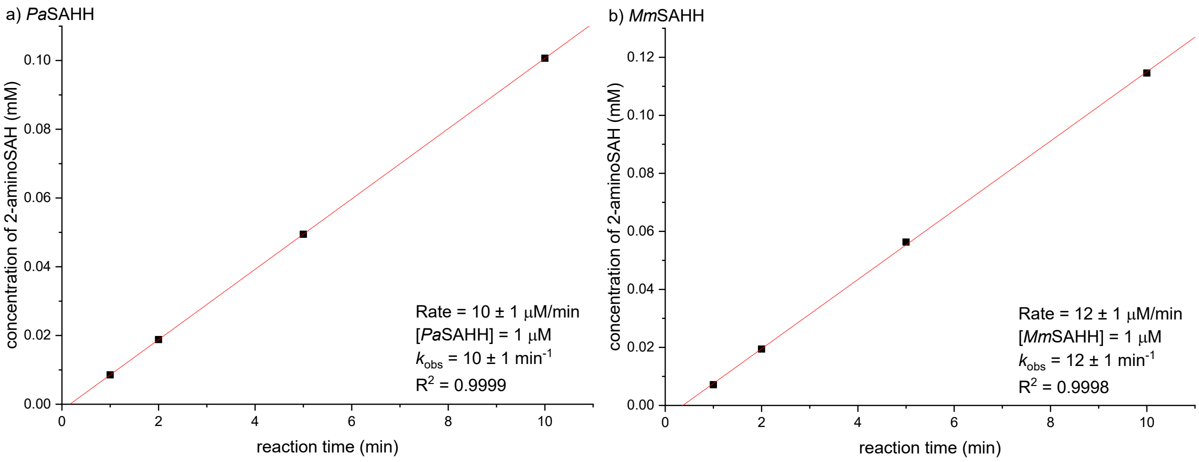

Figure S90. The observed rate constant (k_{obs}) of $S^{2\text{-amino}}AH$ production by ACLR-BLH-SAHH enzyme cascade. The concentration of $S^{2\text{-amino}}AH$ was determined by HPLC. k_{obs} is equal to the initial rate of $S^{2\text{-amino}}AH$ formation divided by the concentration of SAHH. a) with *Pa*SAHH; b) with *Mm*SAHH. Reaction conditions: 200 μL reaction containing 1 mM D/L-Hcy thiolactone, 1 mM 2-aminoadenosine, 1 μM *Ao*ACLR, 1 μM *Sc*BLH, 1 μM *Pa*SAHH or *Mm*SAHH in 50 mM sodium phosphate buffer (pH 8.0) at 25 °C.

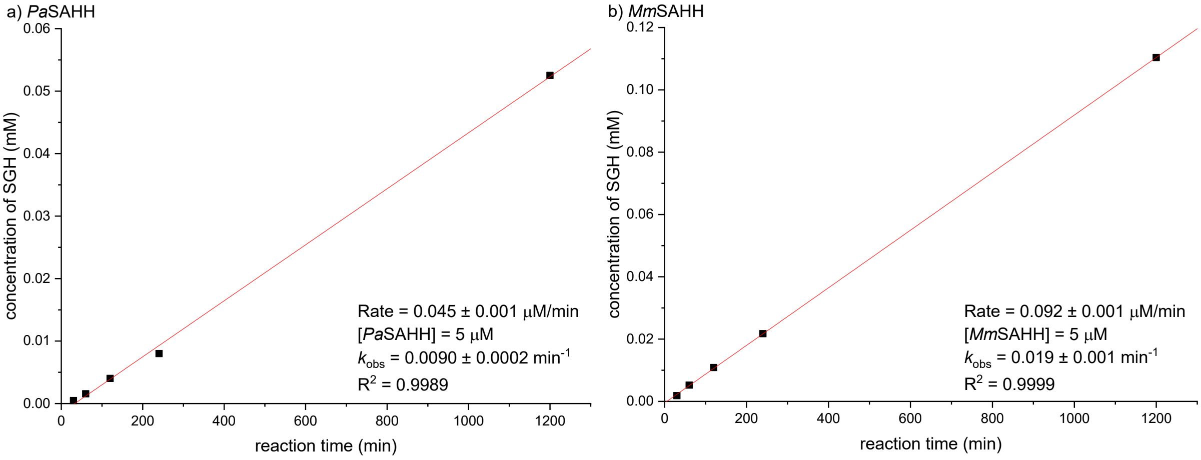

Figure S91. The observed rate constant (k_{obs}) of SGH production by ACLR-BLH-SAHH enzyme cascade. The concentration of SGH was determined by HPLC. k_{obs} is equal to the initial rate of SGH formation divided by the concentration of SAHH. a) with *Pa*SAHH; b) with *Mm*SAHH. Reaction conditions: 200 μL reaction containing 1 mM D/L-Hcy thiolactone, 1 mM guanosine, 1 μM *Ao*ACLR, 1 μM *Sc*BLH, 5 μM *Pa*SAHH or *Mm*SAHH in 50 mM sodium phosphate buffer (pH 8.0) at 25 °C.

Measuring observed rate constants of SAM derivative production from SAH analogs

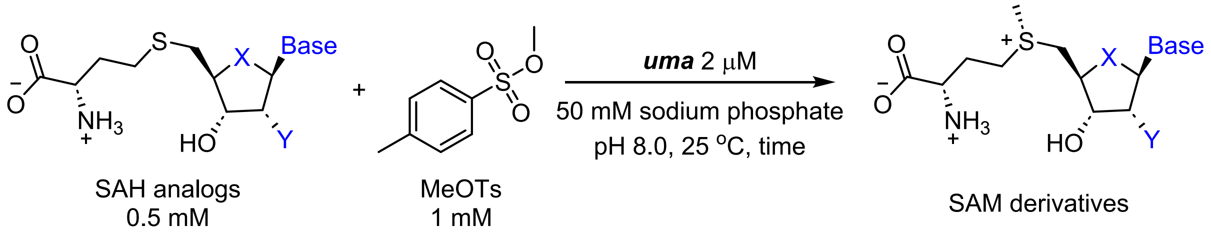

200 μL reaction containing 0.5 mM SAH analogs (diluted from 1 mM SAH analogs stock solution produced by ACLR-BLH-SAHH enzyme cascade), 1 mM MeOTs, 2 μM uma and 50 mM sodium phosphate buffer (pH 8.0) was incubated at 25 °C. To measure the concentration of SAM derivatives, 10 μL of reaction solution was quenched with 10 μL of 1% TFA in water and analyzed by cation-exchange HPLC with 5 μL injection volume. The target products SAM derivatives were confirmed by HR-ESI-MS analysis.

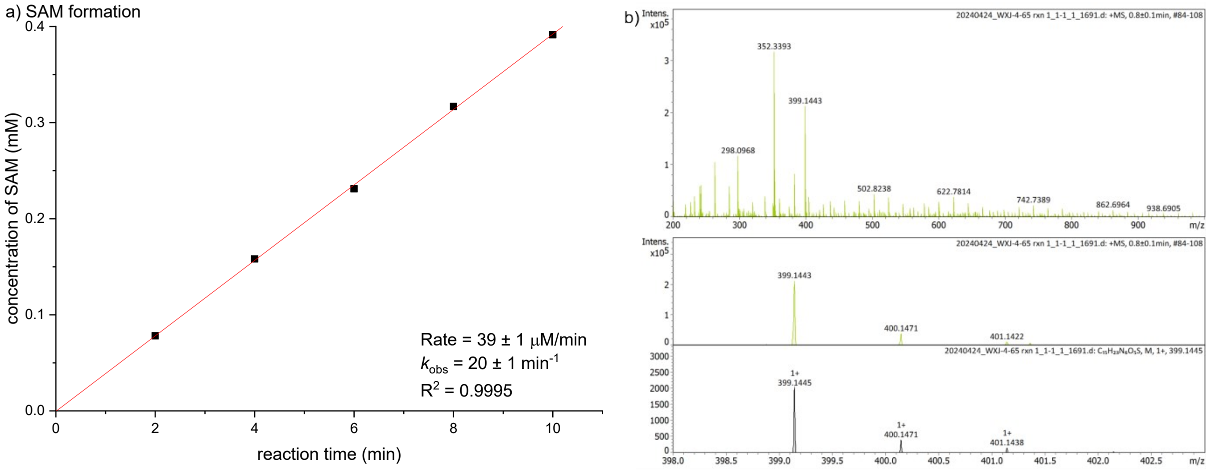

Figure S92. a) The observed rate constant (k_{obs}) of uma-catalyzed SAH methylation using MeOTs as the methyl donor. The concentration of product SAM was determined by HPLC. k_{obs} is equal to the initial rate of SAM formation divided by the concentration of uma (2 μM). b) The HR-ESI-MS spectra of product SAM. Calculated for $C_{15}H_{23}N_6O_5S^+$ (M^+) 399.1445, found 399.1443.

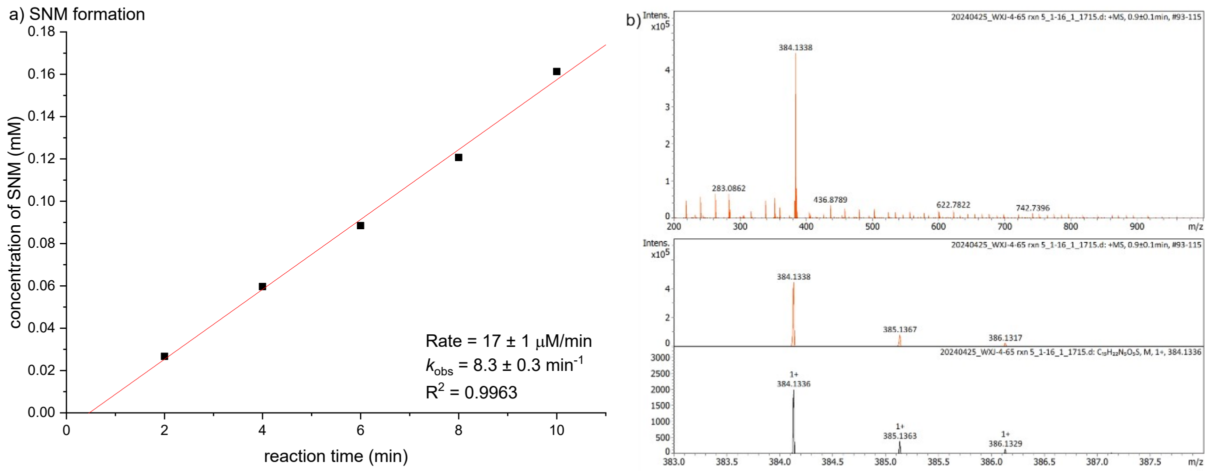

Figure S93. a) The observed rate constant (k_{obs}) of uma-catalyzed SNH methylation using MeOTs as the methyl donor. The concentration of product SNM was determined by HPLC. k_{obs} is equal to the initial rate of SNM formation divided by the concentration of uma (2 μM). b) The HR-ESI-MS spectra of product SNM. Calculated for $C_{15}H_{22}N_5O_5S^+$ (M^+) 384.1336, found 384.1338.

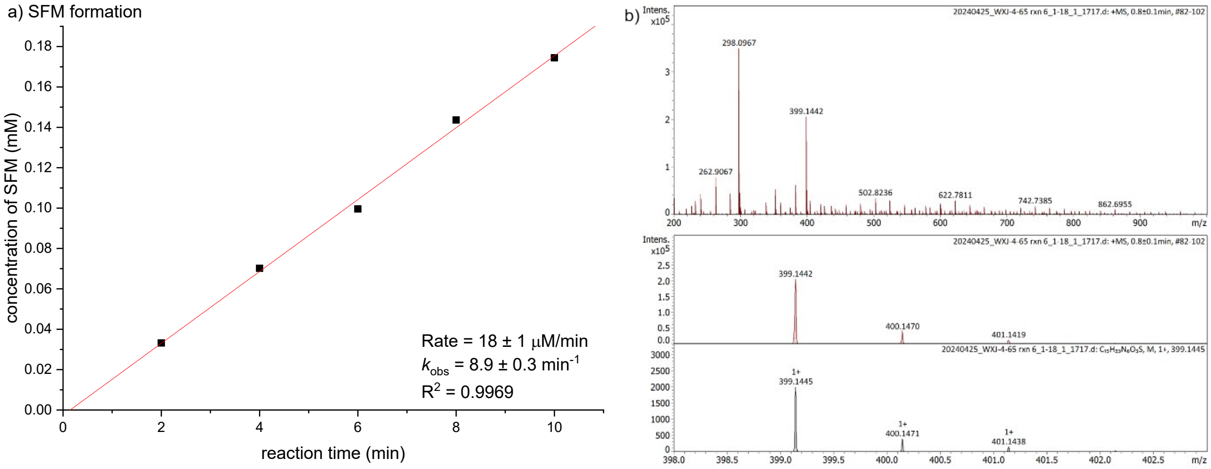

Figure S94. a) The observed rate constant (k_{obs}) of uma-catalyzed SFH methylation using MeOTs as the methyl donor. The concentration of product SFM was determined by HPLC. k_{obs} is equal to the initial rate of SFM formation divided by the concentration of uma (2 μM). b) The HR-ESI-MS spectra of product SFM. Calculated for $C_{15}H_{23}N_6O_5S^+$ (M^+) 399.1445, found 399.1442.

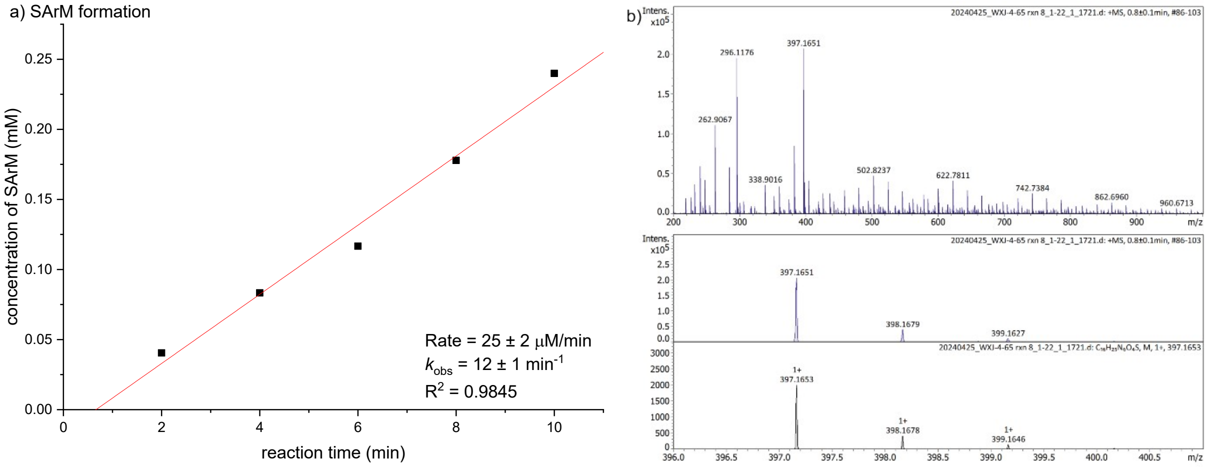

Figure S95. a) The observed rate constant (k_{obs}) of uma-catalyzed SArH methylation using MeOTs as the methyl donor. The concentration of product SArM was determined by HPLC. k_{obs} is equal to the initial rate of SArM formation divided by the concentration of uma (2 μM). b) The HR-ESI-MS spectra of product SArM. Calculated for $C_{16}H_{25}N_6O_4S^+$ (M^+) 397.1653, found 397.1651.

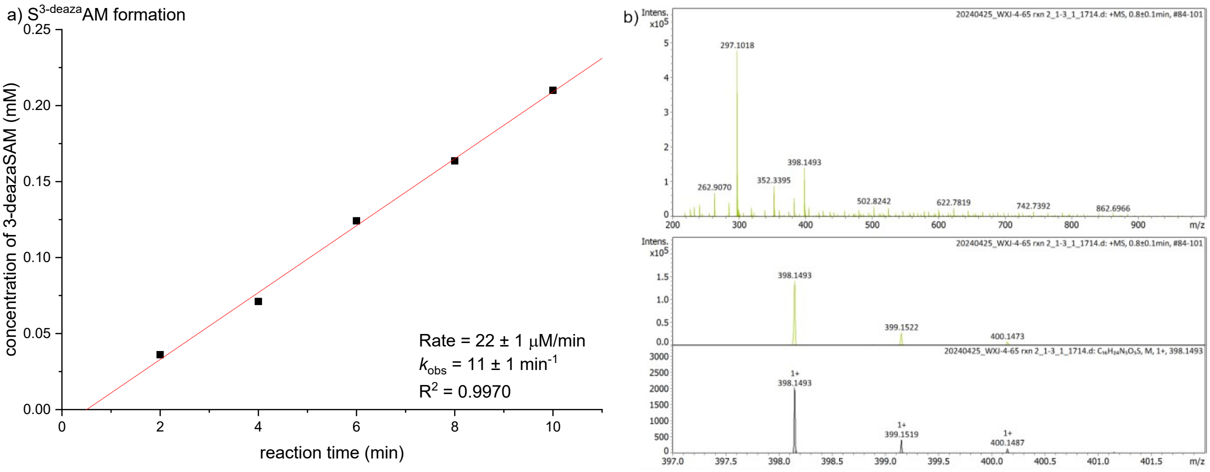

Figure S96. a) The observed rate constant (k_{obs}) of uma-catalyzed S$^{3\text{-deaza}}$AH methylation using MeOTs as the methyl donor. The concentration of product S$^{3\text{-deaza}}$AM was determined by HPLC. k_{obs} is equal to the initial rate of S$^{3\text{-deaza}}$AM formation divided by the concentration of uma (2 μM). b) The HR-ESI-MS spectra of product S$^{3\text{-deaza}}$AM. Calculated for $C_{16}H_{24}N_5O_5S^+$ (M^+) 398.1493, found 398.1493.

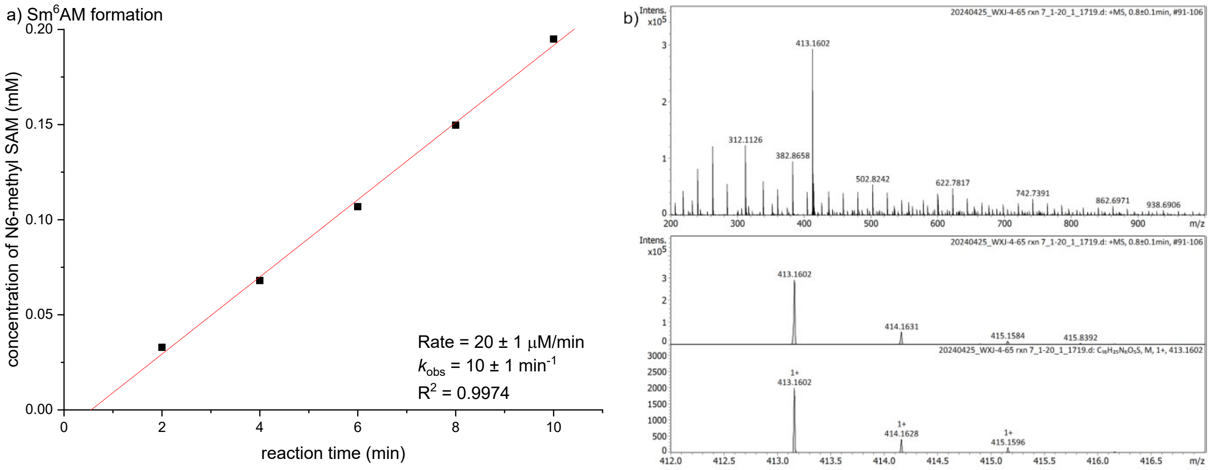

Figure S97. a) The observed rate constant (k_{obs}) of uma-catalyzed Sm^{6}AH methylation using MeOTs as the methyl donor. The concentration of product Sm^{6}AM was determined by HPLC. k_{obs} is equal to the initial rate of Sm^{6}AM formation divided by the concentration of uma (2 μM). b) The HR-ESI-MS spectra of product Sm^{6}AM. Calculated for $C_{16}H_{25}N_6O_5S^+$ (M^+) 413.1602, found 413.1602.

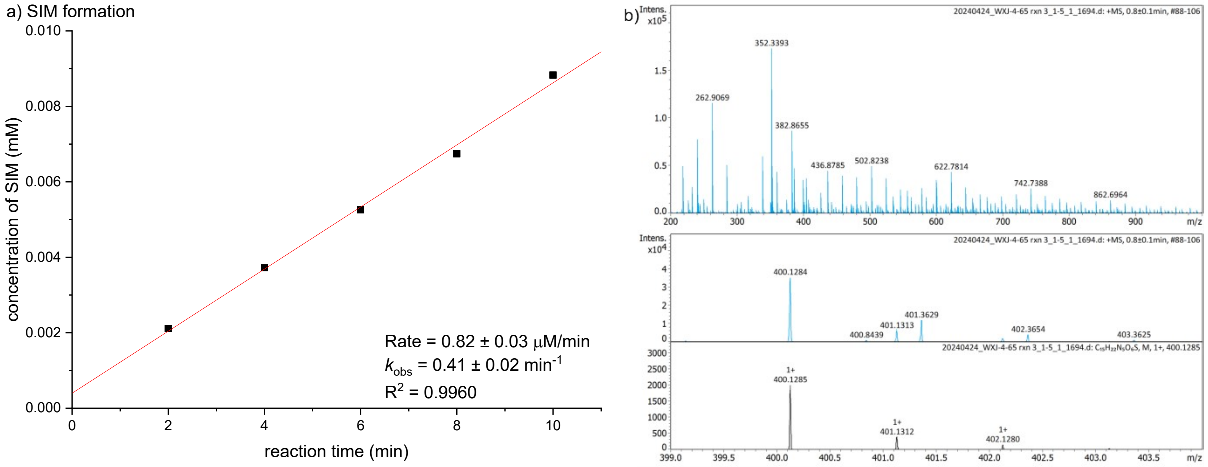

Figure S98. a) The observed rate constant (k_{obs}) of uma-catalyzed SIH methylation using MeOTs as the methyl donor. The concentration of product SIM was determined by HPLC. k_{obs} is equal to the initial rate of SIM formation divided by the concentration of uma (2 μM). b) The HR-ESI-MS spectra of product SIM. Calculated for $C_{15}H_{22}N_5O_6S^+$ (M^+) 400.1285, found 400.1284.

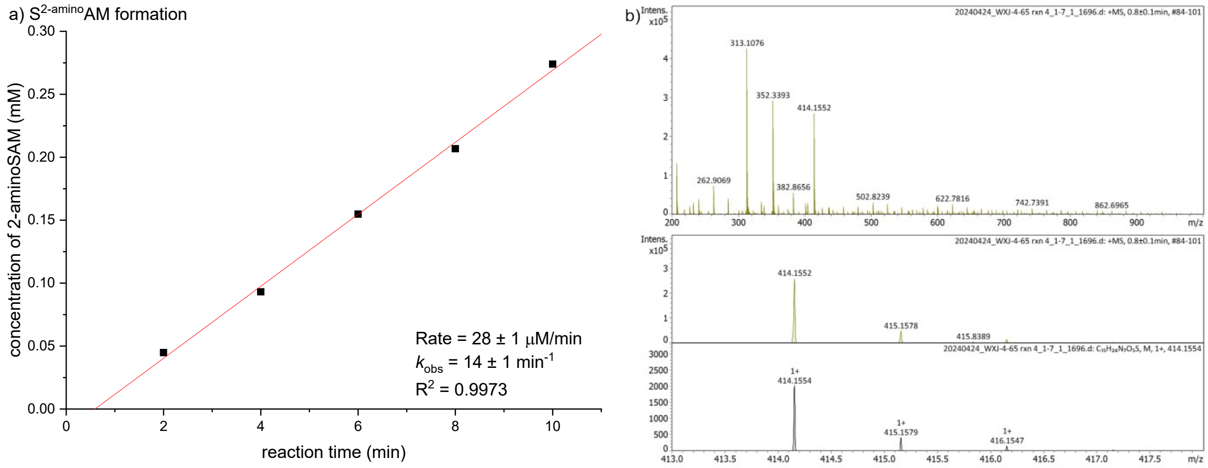

Figure S99. a) The observed rate constant (k_{obs}) of uma-catalyzed $S^{2\text{-amino}}AH$ methylation using MeOTs as the methyl donor. The concentration of product $S^{2\text{-amino}}AM$ was determined by HPLC. k_{obs} is equal to the initial rate of $S^{2\text{-amino}}AM$ formation divided by the concentration of uma (2 μM). b) The HR-ESI-MS spectra of product $S^{2\text{-amino}}AM$. Calculated for $C_{15}H_{24}N_7O_5S^+$ (M^+) 414.1554, found 414.1552.

Stability tests of SAM derivatives

200 μL reaction containing 0.5 mM SAH analogs (diluted from 1 mM SAH analogs stock solution produced by ACLR-BLH-SAHH enzyme cascade), 1 mM MeOTs, 5 μM uma and 50 mM sodium phosphate buffer (pH 8.0) was incubated at 25 °C for 1 h to form SAM derivatives completely. Then the reaction was quenched by adding 200 μL chloroform and vigorous mixing on the vortex. The aqueous layer was isolated by centrifugation and used as a stock solution of 0.5 mM SAM derivatives.

The stock solution of SAM derivatives was incubated at 25 °C and the concentration of SAM derivatives was monitored by HPLC analysis (At different time points, 10 μL of SAM derivative stock solution was added to 10 μL of 1% TFA in water and analyzed by cation-exchange HPLC with 5 μL injection volume). The concentration of SAM derivatives was plotted against time and the graph was fitted with an exponential decay model to calculate the half-life of the SAM derivative.

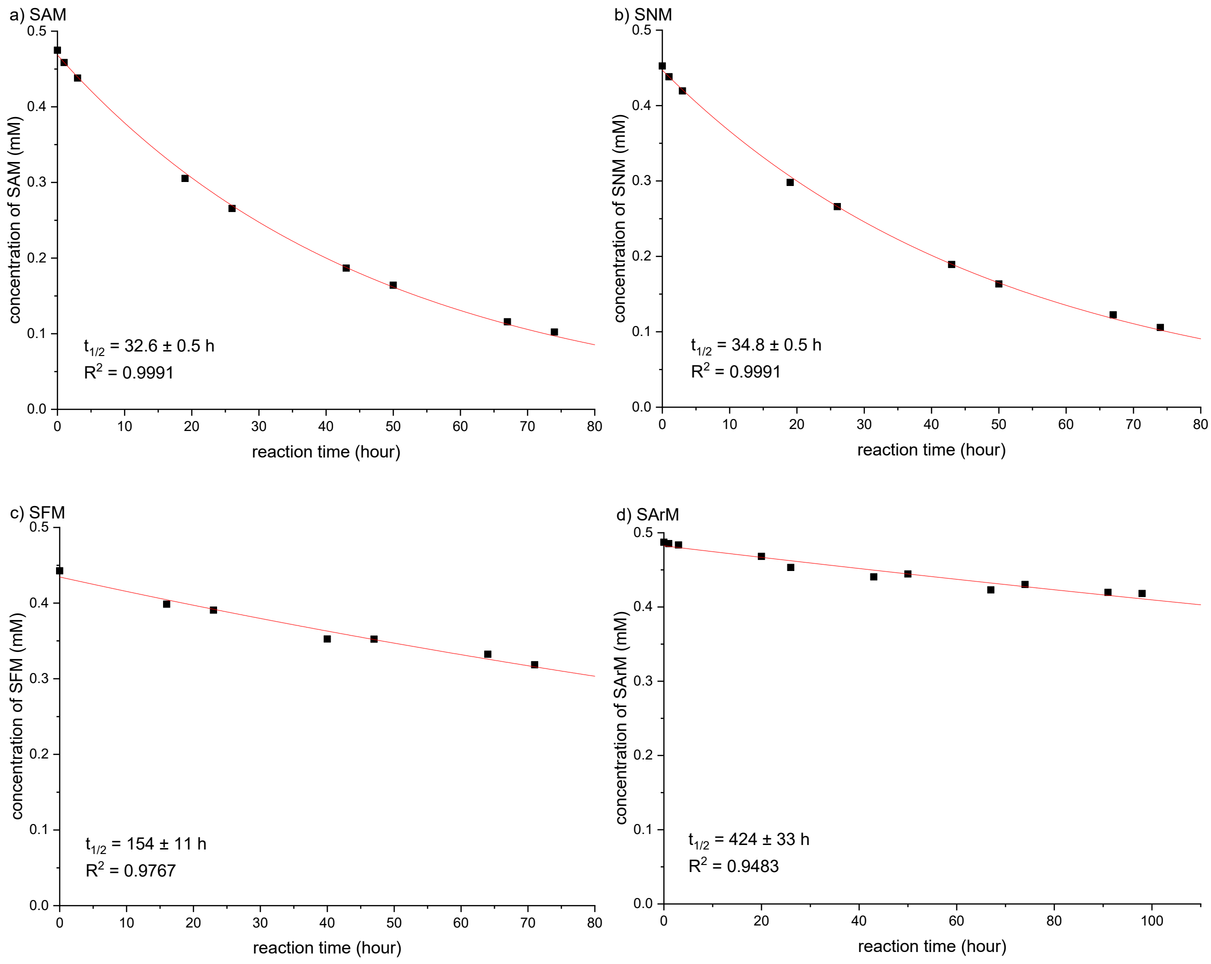

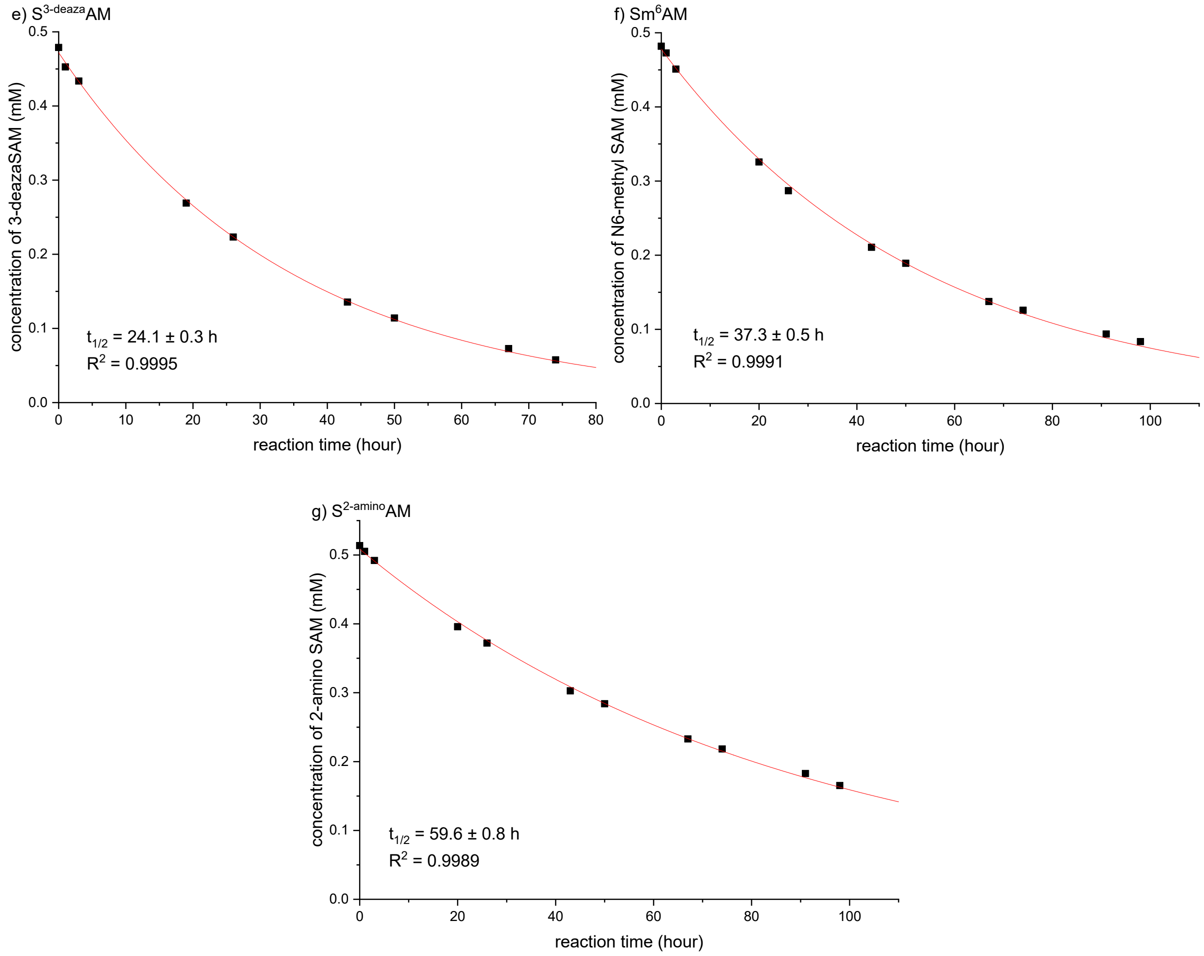

Figure S100. The dot graph of the concentration of SAM in 50 mM sodium phosphate buffer (pH 8.0) over time according to HPLC analysis. a) The calculated half-life of SAM at pH 8.0, 25 °C was (32.6 ± 0.5) hour; b) The calculated half-life of SNM at pH 8.0, 25 °C was (34.8 ± 0.5) hour; c) The calculated half-life of SFM at pH 8.0, 25 °C was (154 ± 11) hour; d) The calculated half-life of SArM at pH 8.0, 25 °C was (424 ± 33) hour; e) The calculated half-life of $S^{3\text{-deaza}}$AM at pH 8.0, 25 °C was (24.1 ± 0.3) hour; f) The calculated half-life of Sm^{6}AM at pH 8.0, 25 °C was (37.3 ± 0.5) hour; g) The calculated half-life of $S^{2\text{-amino}}$AM at pH 8.0, 25 °C was (59.6 ± 0.8) hour.

References

1. K. H. Schülke, F. Ospina, K. Hörnschemeyer, S. Gergel, S. C. Hammer, *ChemBioChem* **2022**, *23*, e202100632.
2. C. Liao, F. P. Seebeck, *Nature Catalysis* **2019**, *2*, 696-701.
3. M. Knipp, O. Braun, M. Vasak, *J. Am. Chem. Soc*. **2005**, *127*, 2372-2373.
4. M. L. Stolowitz, M. J. Minch, *J. Am. Chem. Soc*. **1981**, *103*, 6015-6019.
5. P. Serafinowski, *Synthesis* **1987**, *1987*, 879–883.
6. D. M. Houston, B. Matuszewska, R. T. Borchardt, *J. Med. Chem.* **1985**, *28*, 478–482.
7. P. A Serafinowski, *Synthesis* **1985**, *1985*, 926–928.
8. J. A. Montgomery, H. J. Thomas, M. C. Thorpe, P. K. Chiang, *J. Med. Chem.* **1981**, *24*, 1514–1516.
9. Y. Yao, P. Chen, J. Diao, G. Cheng, L. Deng, J. L. Anglin, B. V. V. Prasad, Y. Song, *J. Am. Chem. Soc.* **2011**, *133*, 16746–16749.
10. H. M. Cohen, A. D. Griffiths, D. S. Tawfik, D. Loakes, *Org. Biomol. Chem.* **2005**, *3*, 152–161.
11. J. Lim, W. C. Winkler, S. Nakamura, V. Scott, R. R. Breaker, *Angew. Chem. Int. Ed.* **2006**, *45*, 964–968.
12. S. S. G. E. van Boom, J. Reedijk, *J. Chem. Soc., Chem. Commun.* **1993**, *18*, 1397–1398.
